# Supplementary material for: A co-anchoring strategy for the synthesis of polar bimodal polyethylene
Source: Nat Commun. 2023 Mar 15;14:1442. doi: 10.1038/s41467-023-37152-1 (PMC10017819; doi:10.1038/s41467-023-37152-1)
Supplement: Supplementary file 1 — Supplementary Information [file 41467_2023_37152_MOESM1_ESM.docx]

**Supplementary Information:**

**A Co-Anchoring Strategy for the Synthesis of Polar Bimodal Polyethylene**

Chen Zou, Quan Wang, Guifu Si*, Changle Chen*

**1. Supplementary Tables and Figures S2**

**2. Supplementary Methods S26**

**3. Supplementary Figures of Characterization of ligands and catalysts S31**

**4. Supplementary Figures of NMR of copolymers S37**

**5. Supplementary Figures of GPC of copolymers.S51**

1. **Supplementary Tables and Figures.**

**Supplementary Table 1. Ethylene polymerization studies with the Ni catalysts.*^a^***

| **Ent** | **Cat.** | **P** | **Yied/g^b^** | **Act. ^b^**  **×10^6^** | **T_m_ ^c^ /^o^C** | ***M*_n_^d^ /10^4^** | ***M*_w_^d^ /10^4^** | **PDI^d^** |
| --- | --- | --- | --- | --- | --- | --- | --- | --- |
| 1 | **Ni1** | 8 | 0.68 | 8.2 | 135.6 | 29.2 | 67.1 | 2.3 |
| 2 | **Ni2** | 8 | 0.55 | 6.6 | 135.5 | 11.6 | 26.7 | 2.3 |
| 3 | **Ni3** | 8 | 0.53 | 3.2 | 123.1 | 1.0 | 1.6 | 1.6 |
| 4 | **Ni1-MgO** | 8 | 0.88 | 52.8 | 138.3 | 241.0 | 556.9 | 2.3 |
| 5 | **Ni2-MgO** | 8 | 0.50 | 31.2 | 136.2 | 86.5 | 177.9 | 2.1 |
| 6 | **Ni3-MgO** | 8 | 0.17 | 10.2 | 126.7 | 1.1 | 2.3 | 2.1 |
| 7 | **Ni1/Ni2-MgO(1:2)** | 8 | 0.70 | 42.0 | 136.2 | 34.8 | 267.8 | 7.7 |
| 8 | **Ni1/Ni2-MgO(1:1)** | 8 | 0.75 | 45.0 | 135.7 | 62.2 | 594.7 | 9.6 |
| 9 | **Ni1/Ni2-MgO(2:1)** | 8 | 0.97 | 58.2 | 136.7 | 91.2 | 449.3 | 4.9 |
| 10 | **Ni1/Ni2-MgO(1:1)** | 30 | 1.55 | 93.0 | 135.0 | 87.8 | 638.4 | 7.2 |
| 11 | **Ni1/Ni3-MgO(1:1)** | 8 | 0.32 | 19.2 | 133.9 | 3.6 | 329.7 | 92.4 |
| 12 | **Ni1/Ni3-MgO(1:2)** | 8 | 0.50 | 12.0 | 132.1 | 5.5 | 232.5 | 42.7 |
| 13 | **Ni1/Ni3-MgO(1:5)** | 8 | 0.72 | 43.2 | 128.8 | 0.7 | 107.7 | 143.1 |
| 14 | **Ni1/Ni3-MgO(1:1)** | 30 | 0.45 | 15.6 | 134.7 | 5.9 | 397.2 | 67.1 |
| 15 | **Ni1/Ni3-MgO(1:5)** | 30 | 1.01 | 59.4 | 130.3 | 2.9 | 164.9 | 57.1 |
| 16 | **Ni2/Ni3-MgO(1:1)** | 8 | 0.43 | 25.8 | 133.6 | 3.0 | 108.9 | 35.8 |
| 17 | **Ni1/Ni3** | 8 | 0.60 | 7.2 | 126.5/136.2 | 3.5 | 31.3 | 8.9 |
| 18 | **Ni2-MgO/Ni3-MgO** | 8 | 0.46 | 27.6 | 125.9/135.8 | 7.0 | 117.1 | 16.7 |

*^a^* Conditions: 1-3, 17, catalyst 0.5 µmol; 4-16, 18, catalyst 0.1 µmol; 5 mL Hep.; t = 10min; T=80^o^C; *^b^* Yields are the average of at least two runs. Activity is in units of 10^6^ g/(mol cat. × h). *^c^* Determined by differential scanning calorimetry (DSC, second heating). *^d^M*_n_: 10^4^ g mol^-1^, *M*_n_ and PDI determined by GPC in trichlorobenzene at 160 ^o^C.

**Supplementary Table 2 Peak fitting data of molecular weight of polar bimodal copolymers in Table 1.^a^**

| **Ent.** | **Cat.** | **Mon./mol/L** | ***M*_n_**  **/10^4^** | ***M*_w_ ^e^/10^4^** | **PDI ^e^** | ***M*_n1_ ^e^/10^4^** | ***M*_w1_ ^e^/10^4^** | **PDI _1_^e^** | ***M*_n2_ ^e^/10^4^** | ***M*_w2_ ^e^/10^4^** | **PDI _2_ ^e^** |
| --- | --- | --- | --- | --- | --- | --- | --- | --- | --- | --- | --- |
| 1 | **Ni1-MgO** | *t*BA/0.1 | 14.6 | 28.2 | 1.9 |  |  |  |  |  |  |
| 2 | **Ni2-MgO** | *t*BA/0.1 | 24.7 | 92.4 | 3.7 |  |  |  |  |  |  |
| 3 | **Ni3-MgO** | *t*BA/0.1 | 0.5 | 1.5 | 2.9 |  |  |  |  |  |  |
| 4 | **Ni1-MgO** | UAE/0.5 | 30.7 | 81.8 | 2.7 |  |  |  |  |  |  |
| 5 | **Ni2-MgO** | UAE/0.5 | 58.9 | 161.5 | 2.7 |  |  |  |  |  |  |
| 6 | **Ni3-MgO** | UAE/0.5 | 0.6 | 1.7 | 2.8 |  |  |  |  |  |  |
| 7^f^ | **Ni2-MgO** | *t*BA/0.1 | 3.9 | 11.0 | 2.8 |  |  |  |  |  |  |
| 8 | **Ni1/Ni3-MgO(1:1)** | *t*BA/0.1 | 2.0 | 23.1 | 11.5 | 20.7 | 36.5 | 1.8 | 0.8 | 2.4 | 3.1 |
| 9 | **Ni2/Ni3-MgO(1:1)** | *t*BA/0.1 | 1.6 | 54.4 | 34.4 | 23.3 | 81.5 | 3.5 | 0.4 | 1.6 | 3.6 |
| 10^b^ | **Ni1/Ni3-MgO(1:1)** | *t*BA/0.1 | 13.3 | 244.8 | 18.4 | 213.6 | 484.8 | 2.3 | 1.7 | 3.6 | 2.0 |
| 11 | **Ni1/Ni3-MgO(1:5)** | *t*BA/0.1 | 0.9 | 25.1 | 28.5 | 9.0 | 30.5 | 3.4 | 0.5 | 1.6 | 3.1 |
| 12 | **Ni2/Ni3-MgO(1:1)** | UAE/0.5 | 6.8 | 78.9 | 11.7 | 46.2 | 143.7 | 3.1 | 0.4 | 1.2 | 2.9 |
| 13 | **Ni2/Ni3-MgO(1:5)** | UAE/0.5 | 4.0 | 52.6 | 13.3 | 40.6 | 106.7 | 2.6 | 1.4 | 2.7 | 1.9 |
| 14^c^ | **Ni2/Ni3-MgO(1:1)** | UAE/0.5 | 4.7 | 17.4 | 3.7 | 12.3 | 22.6 | 1.8 | 1.2 | 1.5 | 1.3 |
| 15^d^ | **Ni2/Ni3-MgO(1:1)** | UAE/0.5 | 8.8 | 117.2 | 13.3 | 95.7 | 179.1 | 1.9 | 2.6 | 5.4 | 2.1 |
| 16 | **Ni1/Ni2/Ni3-MgO(1:1:1)** | UAE/0.5 | 1.7 | 93.4 | 53.9 | - ^f^ | -^f^ | - ^f^ | - ^f^ | - ^f^ | - ^f^ |
| 17 | **Ni2/Ni3-TiO_2_(1:1)** | UAE/0.5 | 4.5 | 50.1 | 11.4 | 17.9 | 71.3 | 4.0 | 1.7 | 4.3 | 2.6 |
| 18 | **Ni2/Ni3-GF (1:1)** | UAE/0.5 | 7.8 | 92.0 | 11.8 | 25.2 | 112.1 | 4.4 | 2.2 | 5.6 | 2.6 |
| 19 | **Ni2/Ni3-APP(1:1)** | UAE/0.5 | 4.0 | 36.1 | 9.0 | 7.1 | 41.4 | 5.9 | 1.1 | 1.7 | 1.5 |
| 20 | **Ni2/Ni3-lignin(1:1)** | UAE/0.5 | 4.0 | 40.5 | 10.2 | 32.3 | 67.2 | 2.1 | 1.8 | 4.7 | 2.6 |
| 21^e^ | **Ni2-MgO/Ni3-MgO** | *t*BA/0.1 | 1.6 | 56.0 | 34.2 | 20.1 | 79.7 | 4.0 | 0.4 | 1.3 | 3.4 |
| 22^e^ | **Ni2-MgO/Ni3-MgO** | UAE/0.5 | 2.1 | 72.7 | 34.4 | 35.4 | 115.2 | 3.3 | 0.4 | 1.3 | 3.3 |

^a^ Conditions: 1-3, 7-11, and 21, cat. 5 µmol (Ni); 4-6, 12-20, 22, cat. 1 µmol (Ni); 5 mL Hep.; *t* = 30 min; *T* = 80 ^o^C; 8 atm. ^e^ *M*_n_: 10^4^ g mol^-1^, *M*_n_, *M*_w_, and *M*_w_/*M*_n_ were determined by gel permeation chromatography in 1,2,4-trichlorobenzene at 160 ^o^C. *M*_n1_, *M*_w1_, PDI_1_, *M*_n2_, *M*_w2_, and PDI_2_ were calculated by Gauss formula and fitting the copolymers generated by two nickel catalysts respectively. GF: glass fiber; APP: ammonium polyphosphate. ^b^ *T* = 120 ^o^C. ^c^ *P* = 30 atm. ^d^ *T* = 120 ^o^C, 30 atm. *^e^* Molar ratio of heterogeneous catalysts **Ni2-MgO** and **Ni3-MgO** was 1: 1. ^f^ The sample was generated by three kinds of nickel catalysts and cannot be accurately sealed and fitted.

***Gauss formula***

**
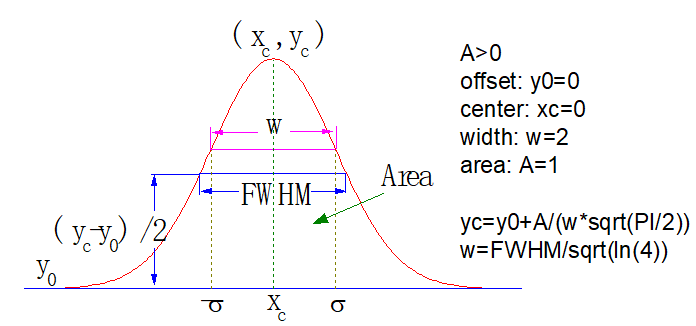

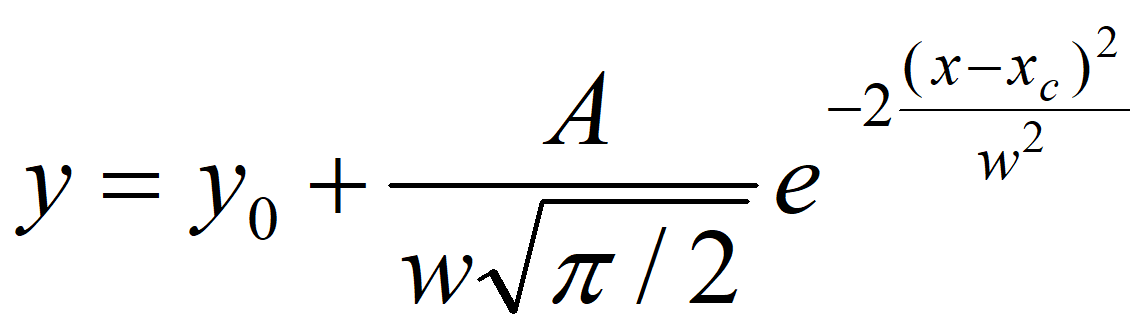
**

***
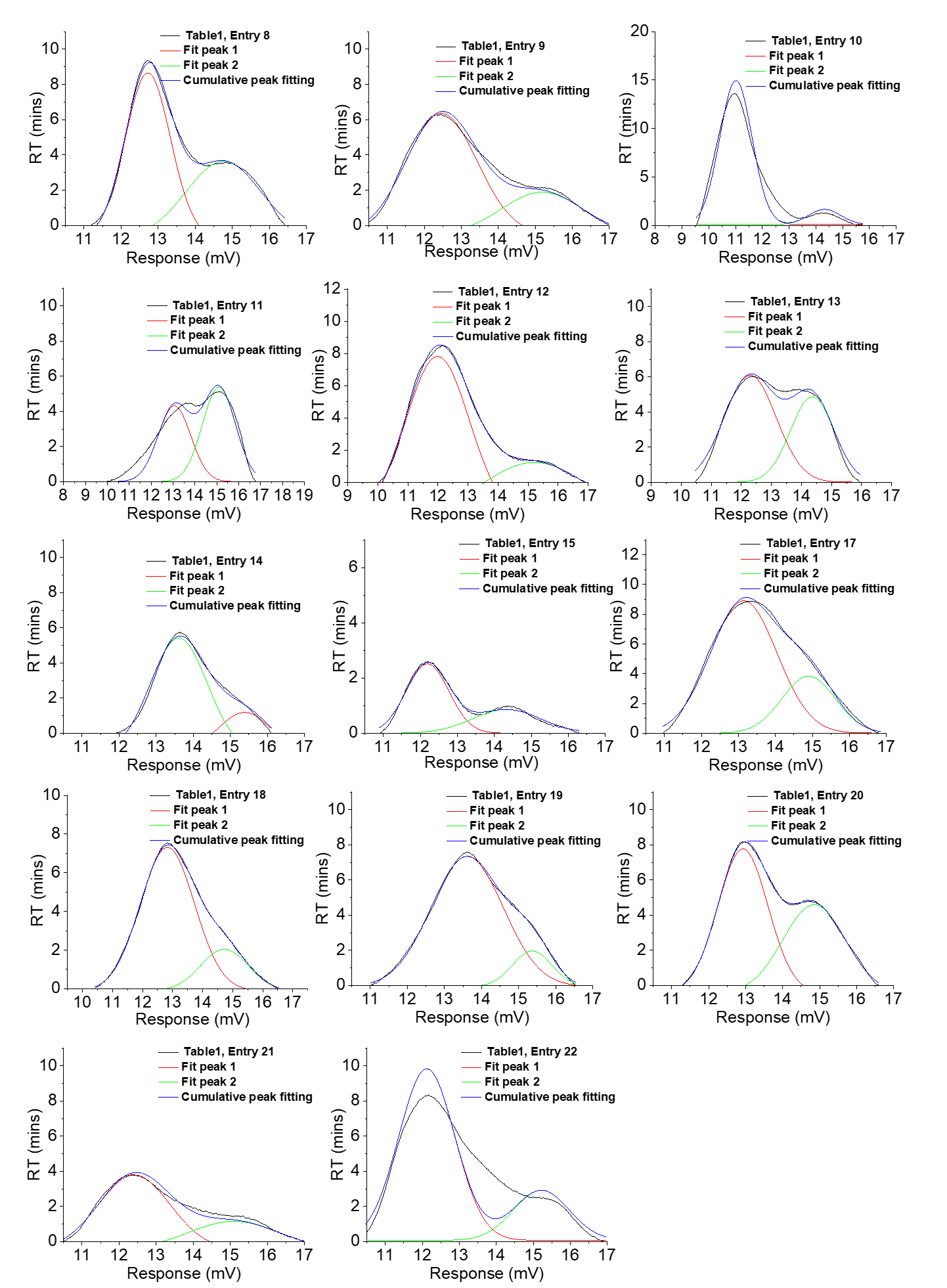
***

**Supplementary Figure 1.** Peak fitting curves of molecular weight of polar bimodal copolymers in Table 1.

**Supplementary Table 3. Ethylene copolymerization with Ni catalysts.*^a^***

| ***Ent*** | ***Cat.*** | **Mon.**  **(mol/L)** | **Yield**  **/g *^b^*** | **Act. *^b^ (10^5^)*** | ***Incorp.* %*^c^*** | ***T*_m_*^d^*/ ^o^C** | ***M_n_^e^/10^4^*** | ***M_w_^e^/10^4^*** | **PDI*^e^*** |
| --- | --- | --- | --- | --- | --- | --- | --- | --- | --- |
| 1 | **Ni1** | UAE0.5 | 0.77 | 6.2 | 0.5 | 132.9 | 8.1 | 24.3 | 3.0 |
| 2 | **Ni2** | UAE0.5 | 0.61 | 4.9 | 0.5 | 127.8 | 6.6 | 14.5 | 2.2 |
| 3 | **Ni3** | UAE0.5 | 0.38 | 3.0 | 0.7 | 122.0 | 0.9 | 2.6 | 2.9 |
| 4 | **Ni1/Ni3** | UAE0.5 | 0.58 | 4.6 | 0.6 | 125.0/132.9 | 2.4 | 15.4 | 6.4 |

*^a^* Conditions: 2.5 µmol; 5 mL Hep.; t = 30 min; T = 80 ^o^C; 8 atm. *^b^* Yields are the average of at least two runs. Activity is in units of 10^5^ g/(mol cat. × h).*^c^* Incorporation ratios of comonomers were determined from ^1^H NMR spectra. *^d^* Determined by differential scanning calorimetry (DSC, second heating) *^e^ M*_n_: 10^4^ g mol^-1^, *M*_n_, *M*_w_ and PDI were determined by gel permeation chromatography (GPC) in 1,2,4-trichlorobenzene at 160 ^o^C.

**Supplementary Table 4. Synthesis of a series of polar bimodal polyethylene. ^a^**

| **Ent.** | **Cat.** | **Mon.**  **mol/L** | **Yield/g^b^** | **Act. ^b^(10^5^)** | **X_M_^c^**  **(%)** | ***T*_m_^d^/^o^C** | ***M*_n_ ^e^/10^4^** | ***M*_w_ ^e^/10^4^** | **PDI^e^** |
| --- | --- | --- | --- | --- | --- | --- | --- | --- | --- |
| 1 | **Ni2/Ni3-MgO(1:1)** | - | 0.43 | 258.0 | 0 | 133.6 | 3.0 | 108.9 | 35.8 |
| 2 | **Ni2/Ni3-MgO(1:1)** | 0.5 | 0.62 | 12.4 | 0.5 | 132.2 | 6.8 | 78.9 | 11.7 |
| 3 | **Ni2/Ni3-MgO(1:5)** | 0.5 | 0.33 | 6.6 | 0.9 | 129.1 | 4.0 | 52.6 | 13.3 |
| 4*^f^* | **Ni2/Ni3-MgO(1:1)** | 0.5 | 0.58 | 11.6 | 1.3 | 126.4 | 4.7 | 17.4 | 3.7 |
| 5 | **Ni2/Ni3-MgO(1:5)** | 1.0 | 0.39 | 7.8 | 1.7 | 121.5 | 0.7 | 7.3 | 10.0 |
| 6 | **Ni2-MgO/Ni3-MgO**  **(1:1)** | - | 0.43 | 276.0 | 0 | 125.9/  135.8 | 7.0 | 117.1 | 16.7 |
| 7 | **Ni2-MgO/Ni3-MgO**  **(1:1)** | 0.5 | 0.52 | 1.0 | 0.5 | 125.6/  131.1 | 2.1 | 72.7 | 32.4 |
| 8 | **Ni2-MgO/Ni3-MgO**  **(1:5)** | 0.5 | 0.35 | 7.0 | 0.9 | 122.8/126.6 | 1.0 | 57.5 | 55.2 |
| 9 *^f^* | **Ni2-MgO/Ni3-MgO**  **(1:1)** | 0.5 | 0.47 | 9.4 | 1.3 | 116.5/128.2 | 1.28 | 14.5 | 11.3 |
| 10 | **Ni2-MgO/Ni3-MgO**  **(1:5)** | 1.0 | 0.36 | 7.2 | 1.7 | 109.3/122.91 | 0.8 | 7.6 | 9.9 |

^a^ Conditions: cat. 1 µmol (Ni); 5 mL Hep.; *t* = 30 min; *T* = 80 ^o^C; P_ethylene_ = 8 atm. Comonomer: methyl 10-undecenoate. ^b^ Yields are the average of at least two runs. Activity is in units of 10^5^ g/(mol cat. × h).^c^ Incorporation ratios of comonomers were determined from ^1^H NMR spectra. ^d^ Determined by differential scanning calorimetry (DSC, second heating) ^e^ *M*_n_: 10^4^ g mol^-1^, *M*_n_, *M*_w_, and PDI were determined by gel permeation chromatography in 1,2,4-trichlorobenzene at 160 ^o^C. ^f^ *T* = 120 ^o^C, 8 atm.


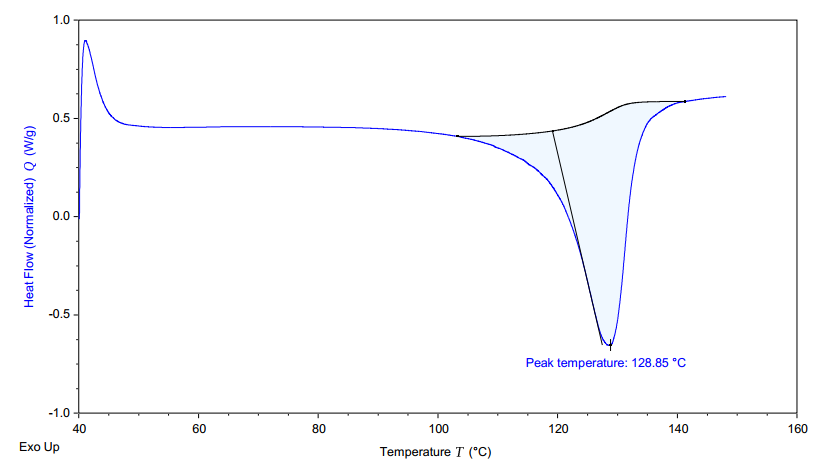


**Supplementary Figure 2.** DSC of the polymer from Table 1, Entry 1.


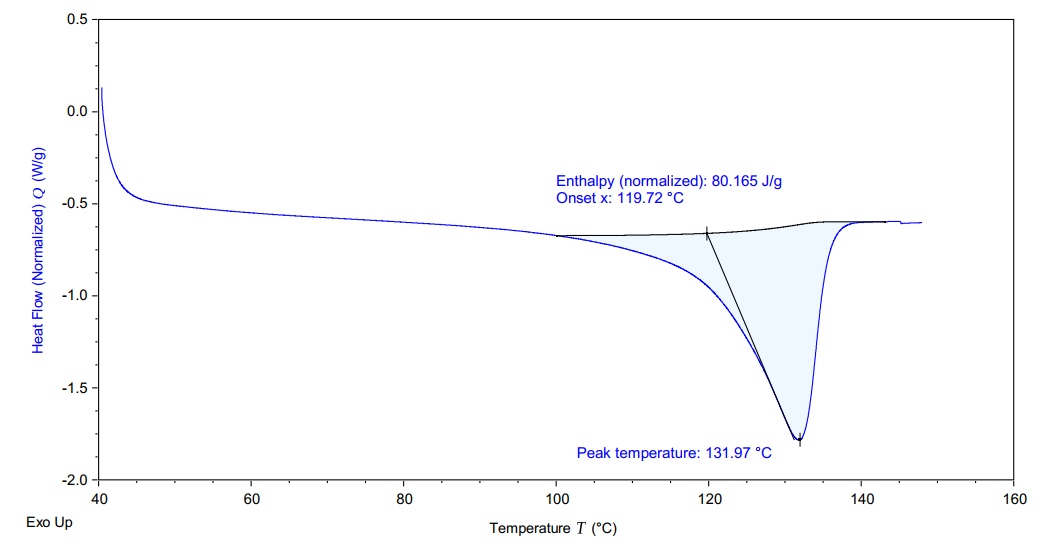


**Supplementary Figure 3.** DSC of the polymer from Table 1, Entry 2.


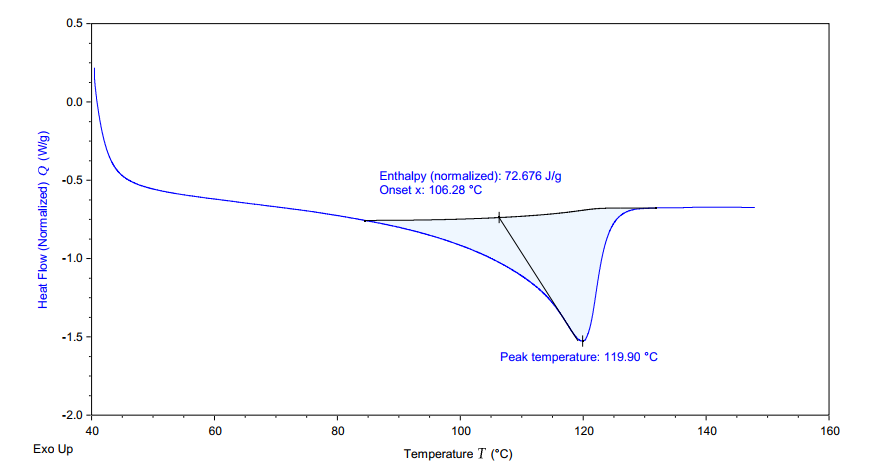


**Supplementary Figure 4.** DSC of the polymer from Table 1, Entry3.


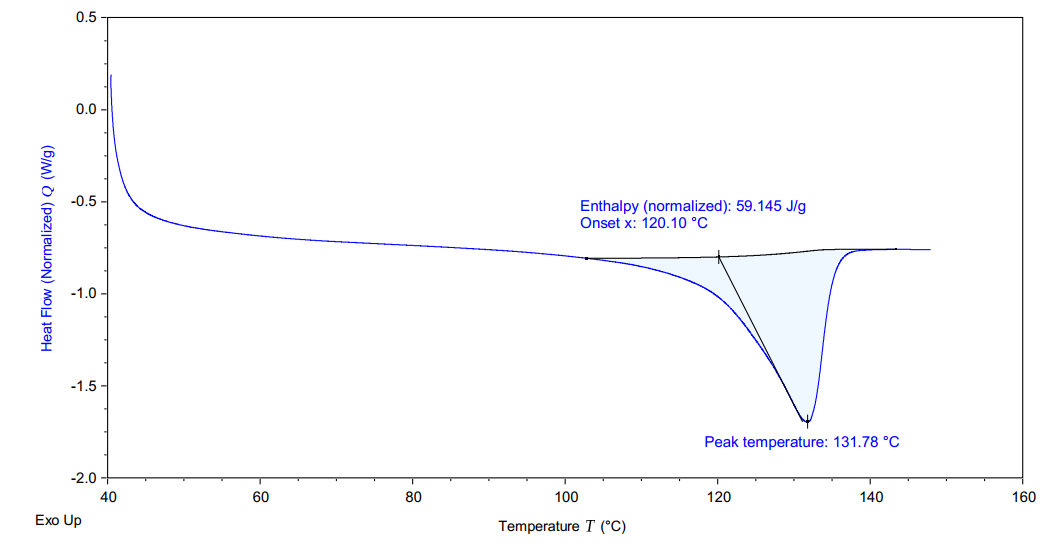


**Supplementary Figure 5.** DSC of the polymer from Table 1, Entry 4.


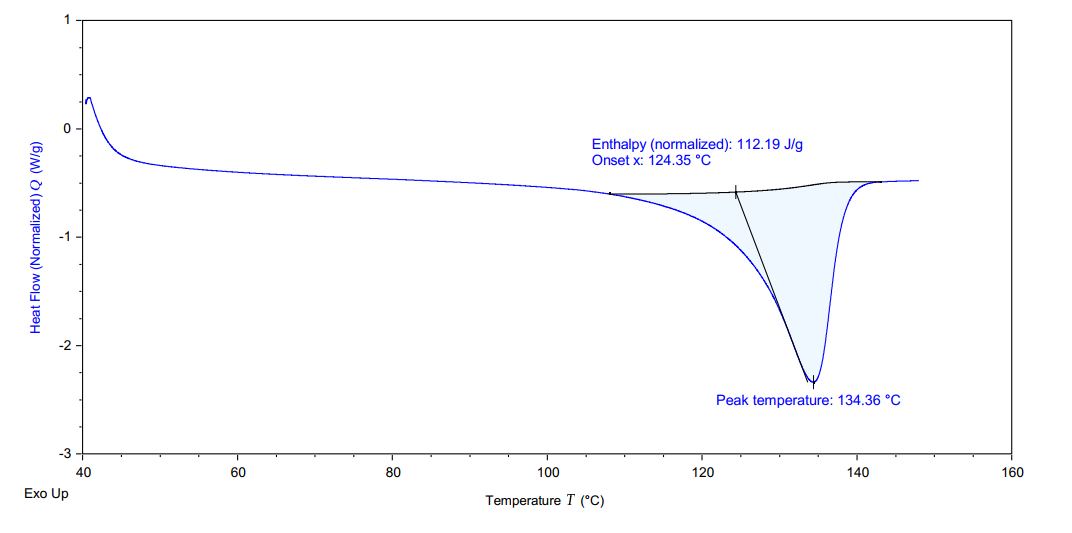


**Supplementary Figure 6.** DSC of the polymer from Table 1, Entry 5.


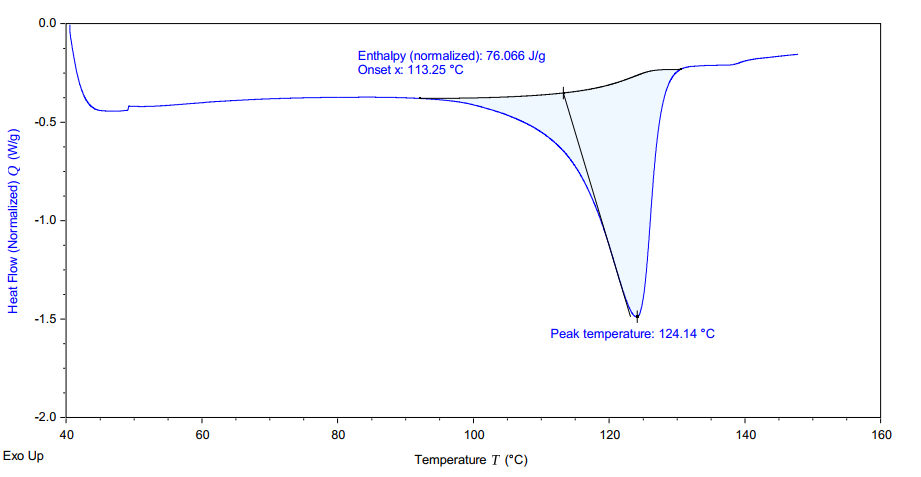


**Supplementary Figure 7.** DSC of the polymer from Table 1, Entry 6.


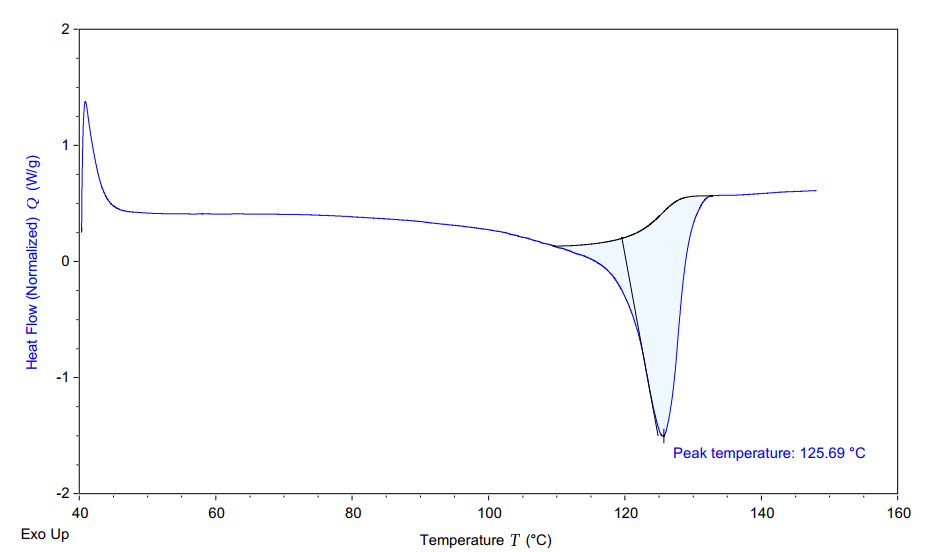


**Supplementary Figure 8.** DSC of the polymer from Table 1, Entry 7.


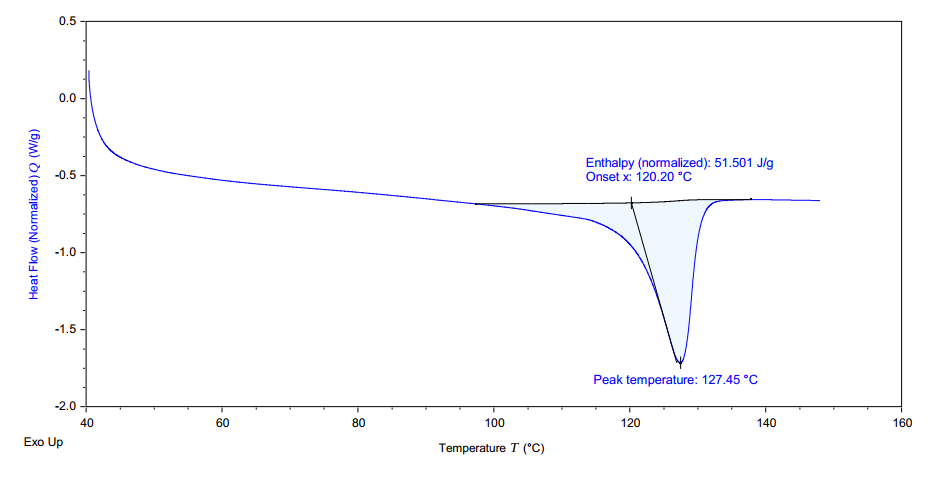


**Supplementary Figure 9.** DSC of the polymer from Table 1, Entry 8.


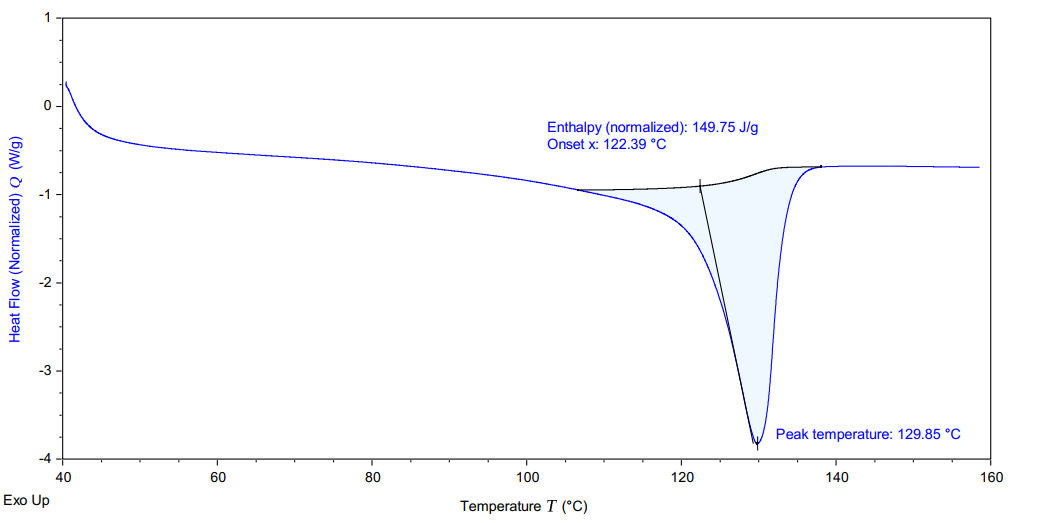


**Supplementary Figure 10.** DSC of the polymer from Table 1, Entry 9.


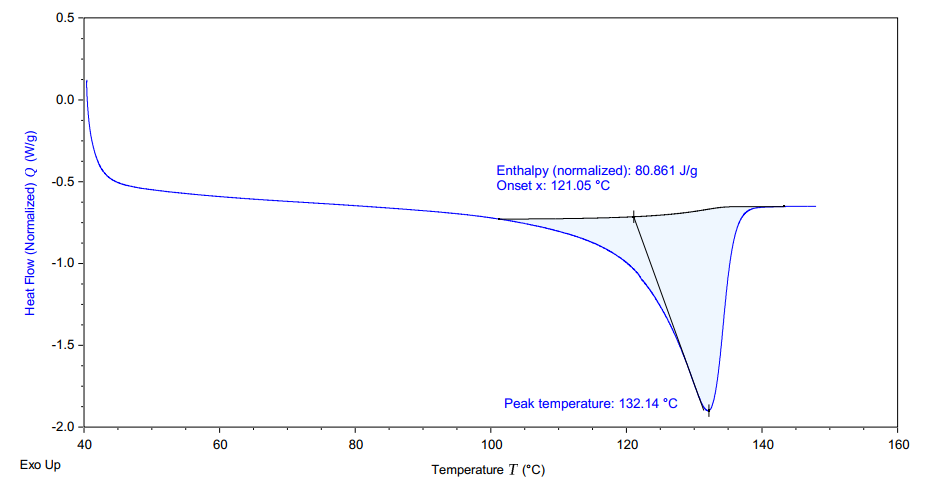


**Supplementary Figure 11.** DSC of the polymer from Table 1, Entry 10.


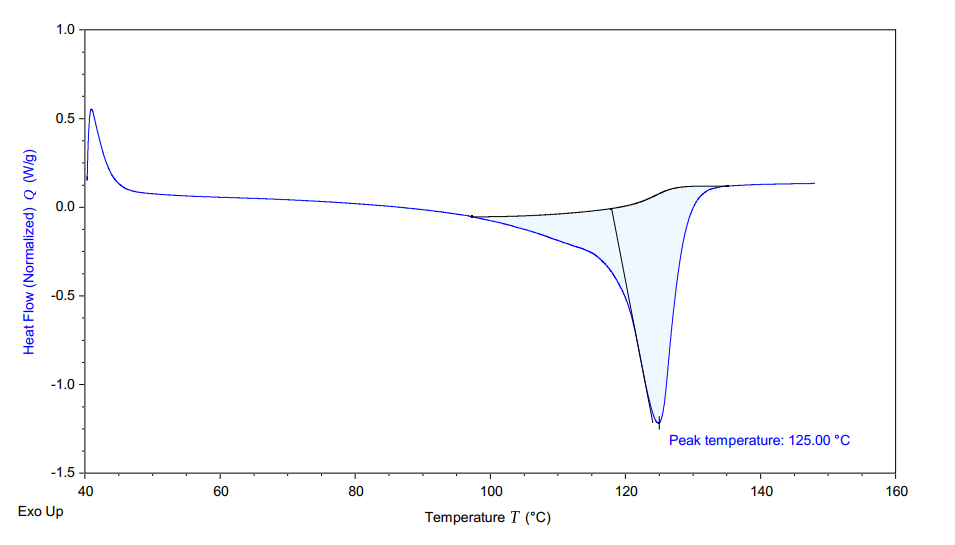


**Supplementary Figure 12.** DSC of the polymer from Table 1, Entry 11.


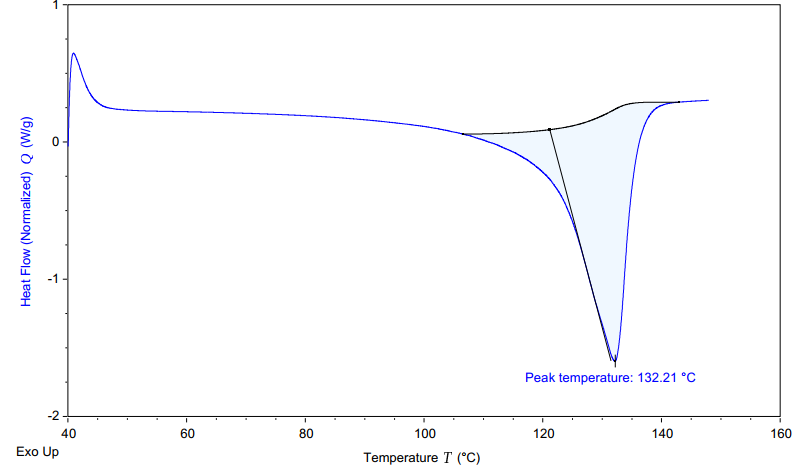


**Supplementary Figure 13.** DSC of the polymer from Table 1, Entry 12.


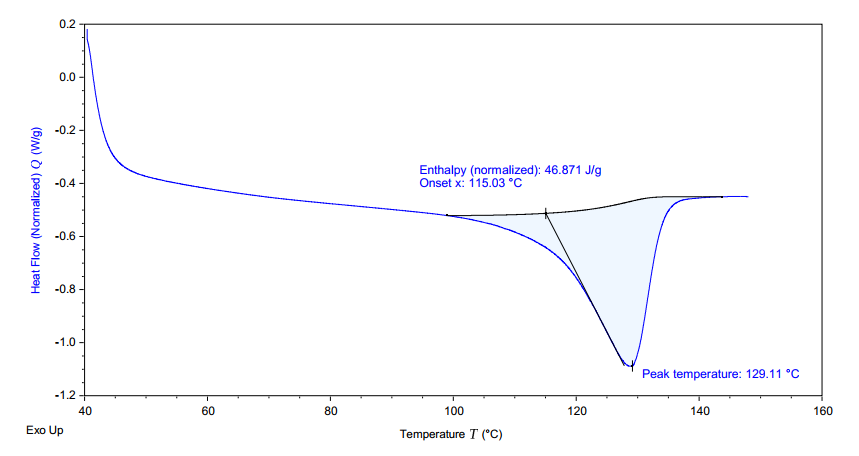


**Supplementary Figure 14.** DSC of the polymer from Table 1, Entry 13.


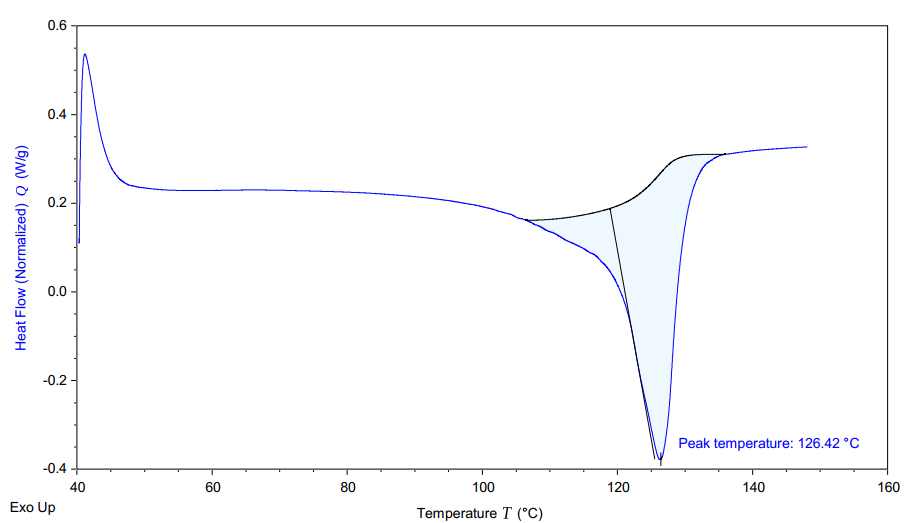


**Supplementary Figure 15.** DSC of the polymer from Table 1, Entry 14.


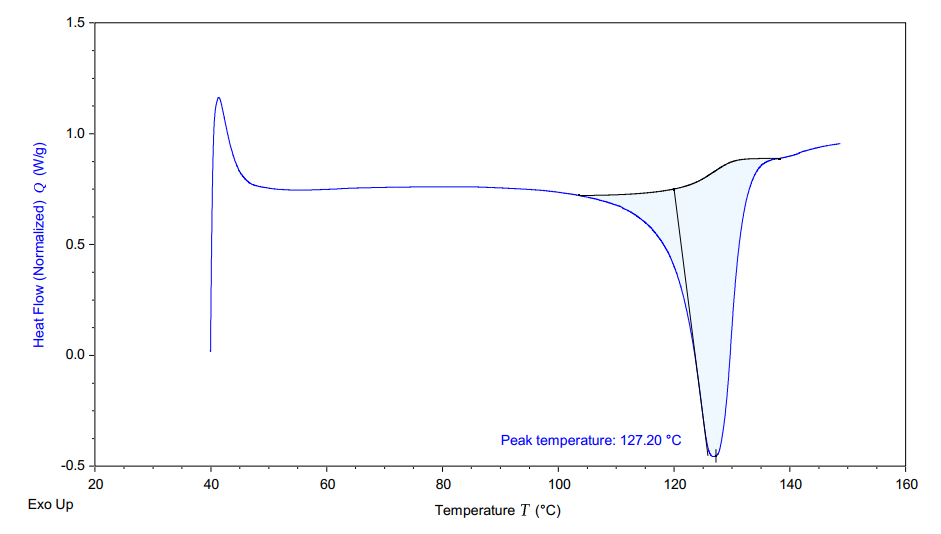


**Supplementary Figure 16.** DSC of the polymer from Table 1, Entry 15.


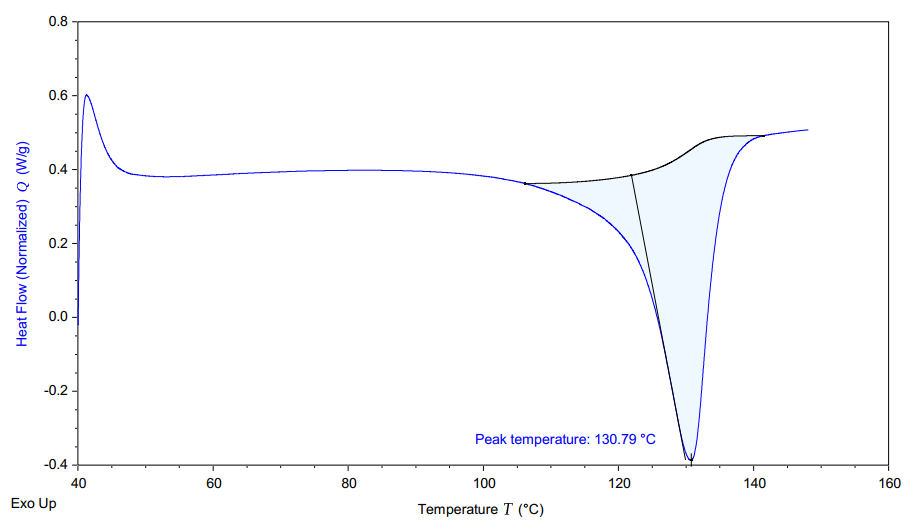


**Supplementary Figure 17.** DSC of the polymer from Table 1, Entry 16.


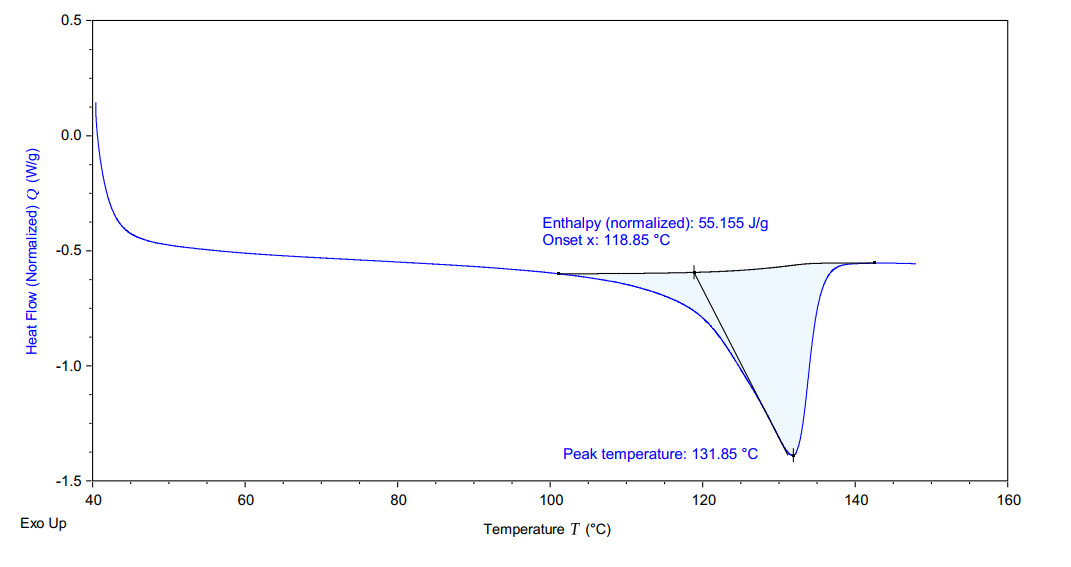


**Supplementary Figure 18.** DSC of the polymer from Table 1, Entry 17.


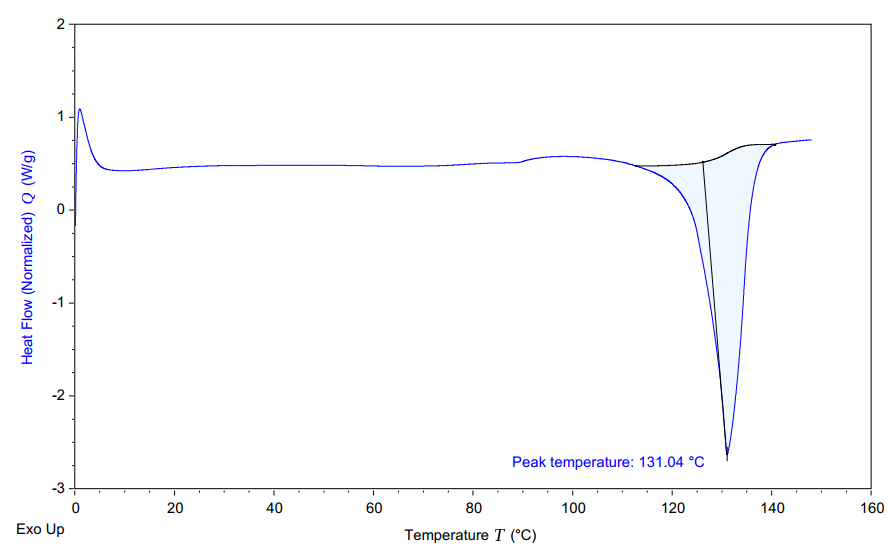


**Supplementary Figure 19.** DSC of the polymer from Table 1, Entry 18.


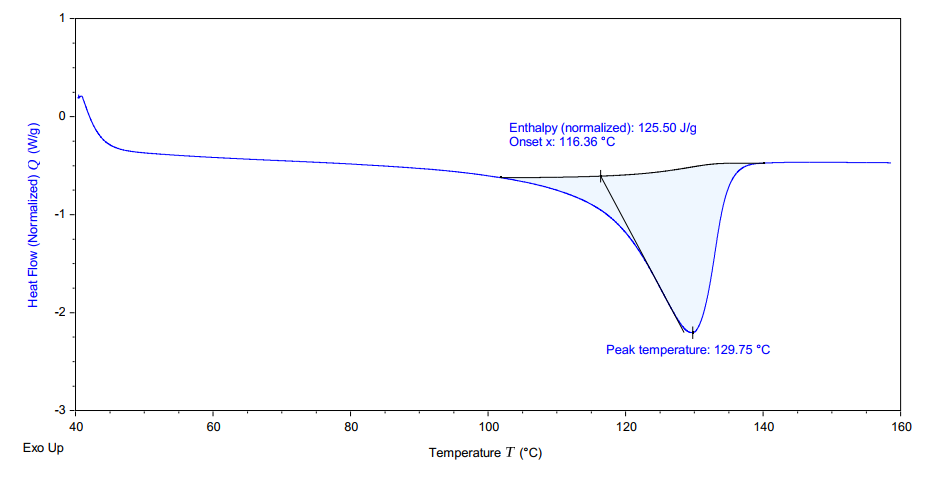


**Supplementary Figure 20.** DSC of the polymer from Table 1, Entry 19.


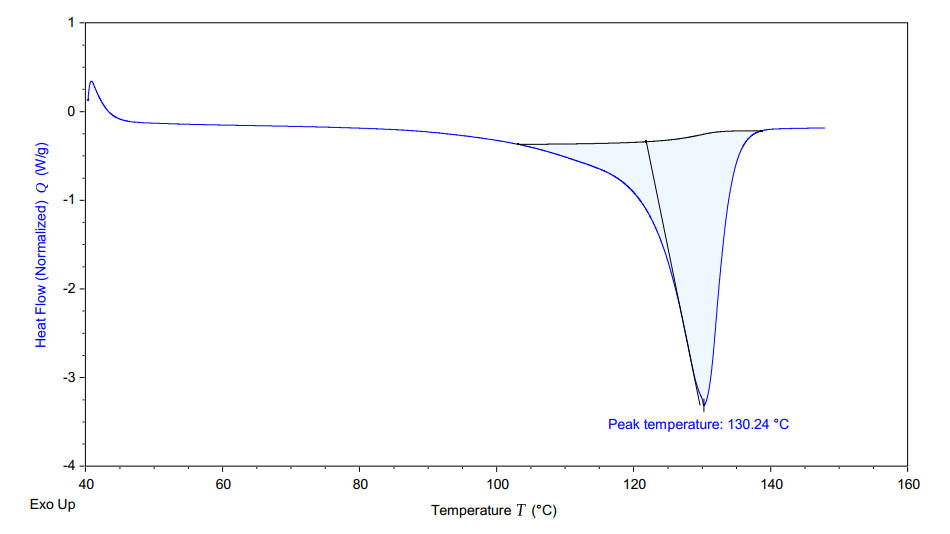


**Supplementary Figure 21.** DSC of the polymer from Table 1, Entry 20.


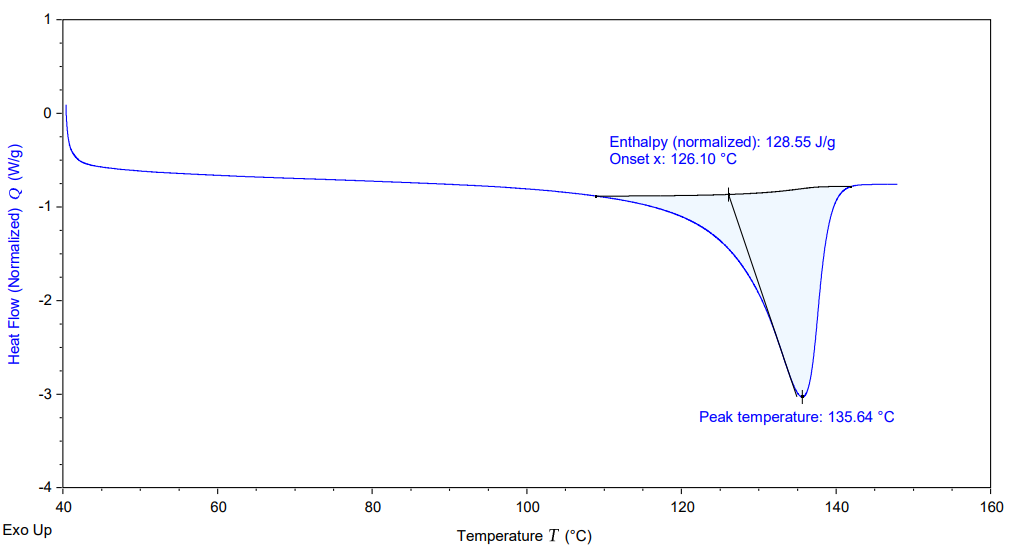


**Supplementary Figure 22.** DSC of the polymer from Supplementary Table 1, Entry 1.


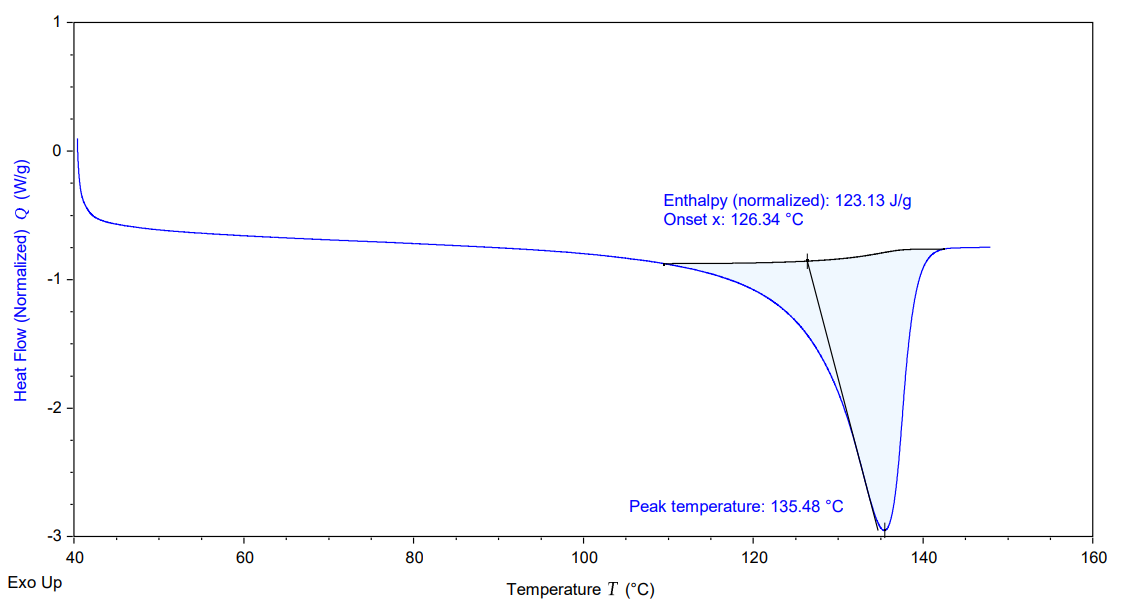


**Supplementary Figure 23.** DSC of the polymer from Supplementary Table 1, Entry 2.


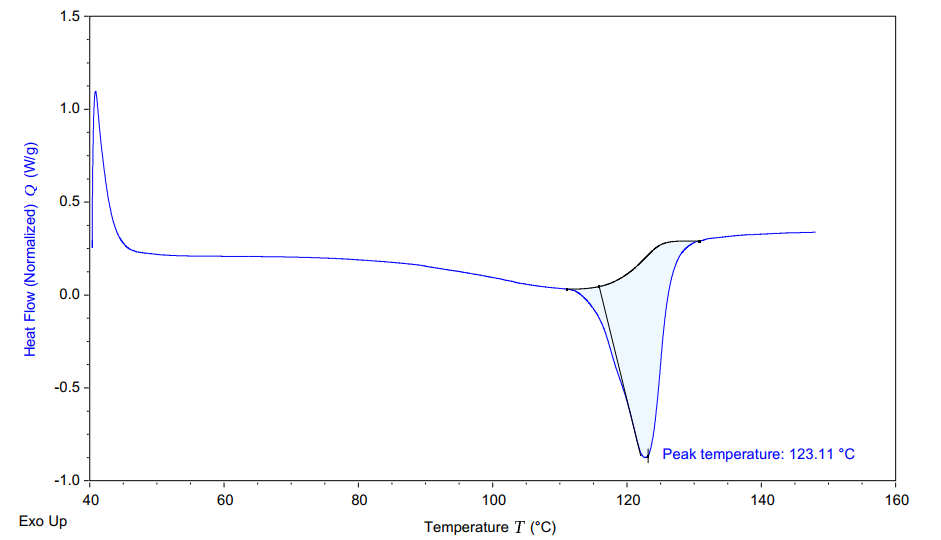


**Supplementary Figure 24.** DSC of the polymer from Supplementary Table 1, Entry 3.


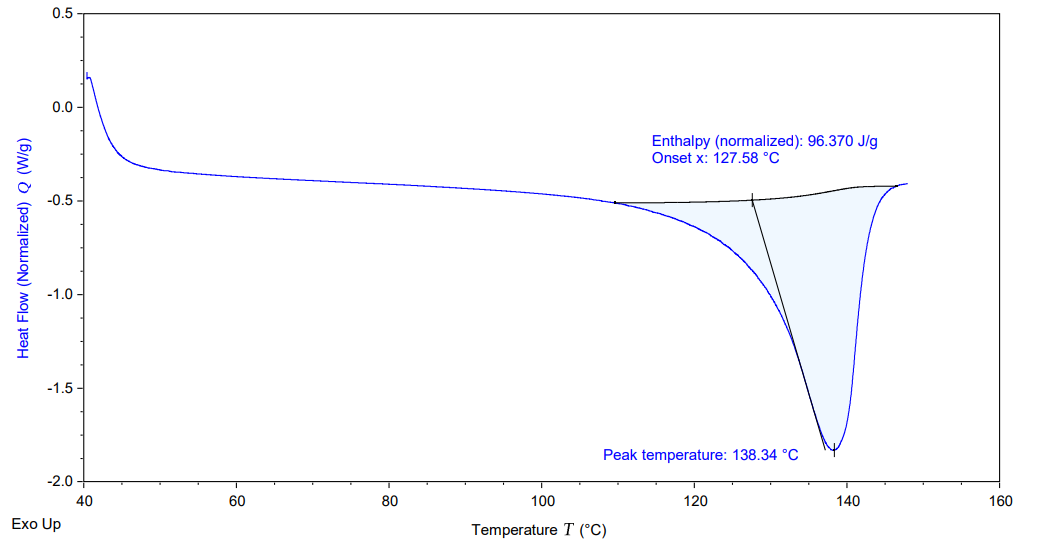


**Supplementary Figure 25.** DSC of the polymer from Supplementary Table 1, Entry 4.


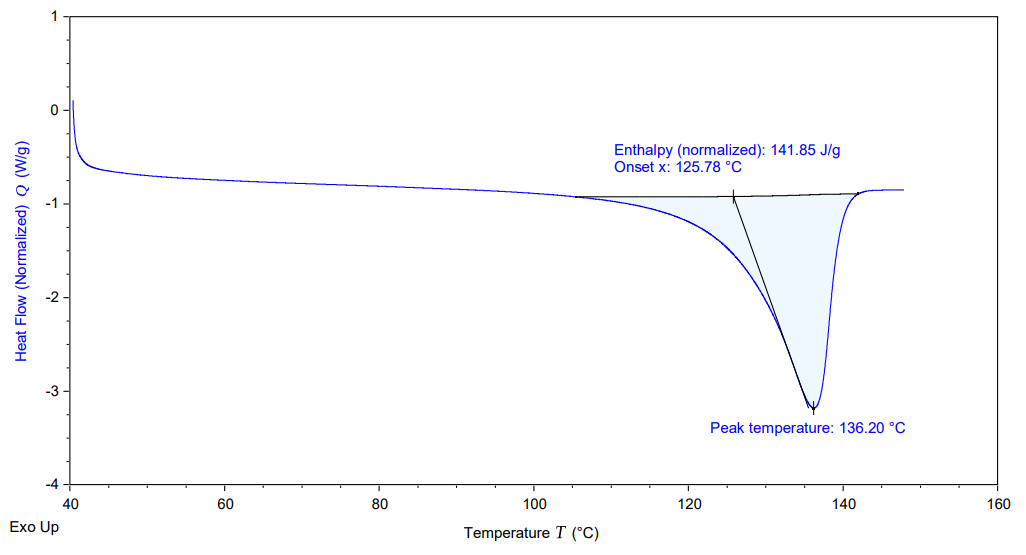


**Supplementary Figure 26.** DSC of the polymer from Supplementary Table 1, Entry 5.


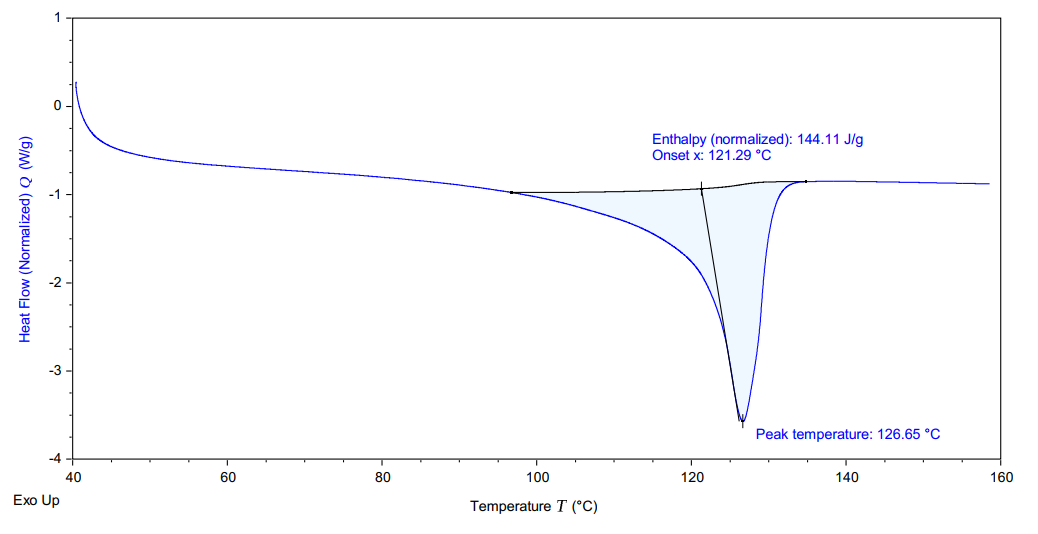


**Supplementary Figure 27.** DSC of the polymer from Supplementary Table 1, Entry6.


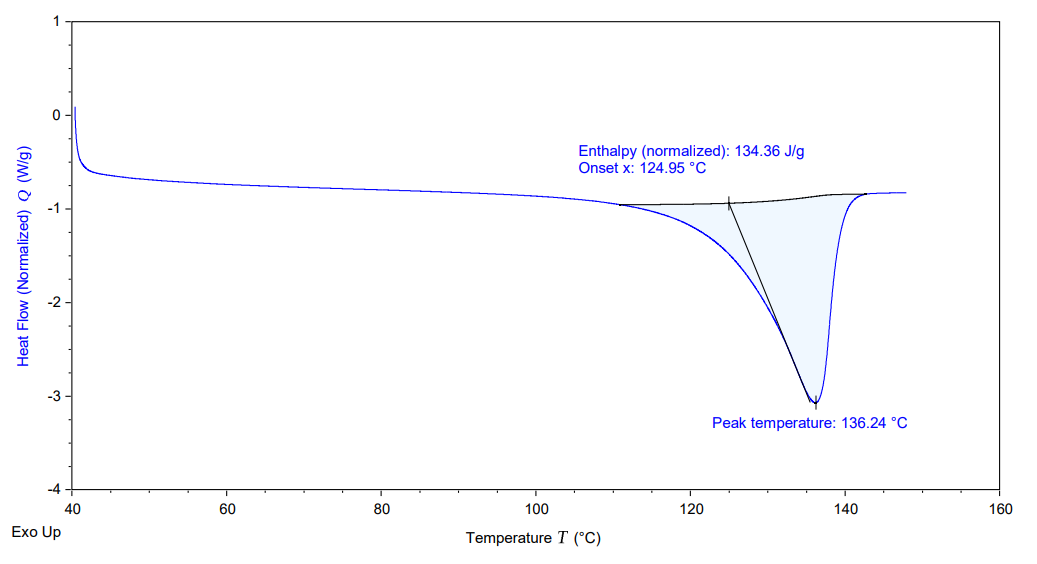


**Supplementary Figure 28.** DSC of the polymer from Supplementary Table 1, Entry 7.


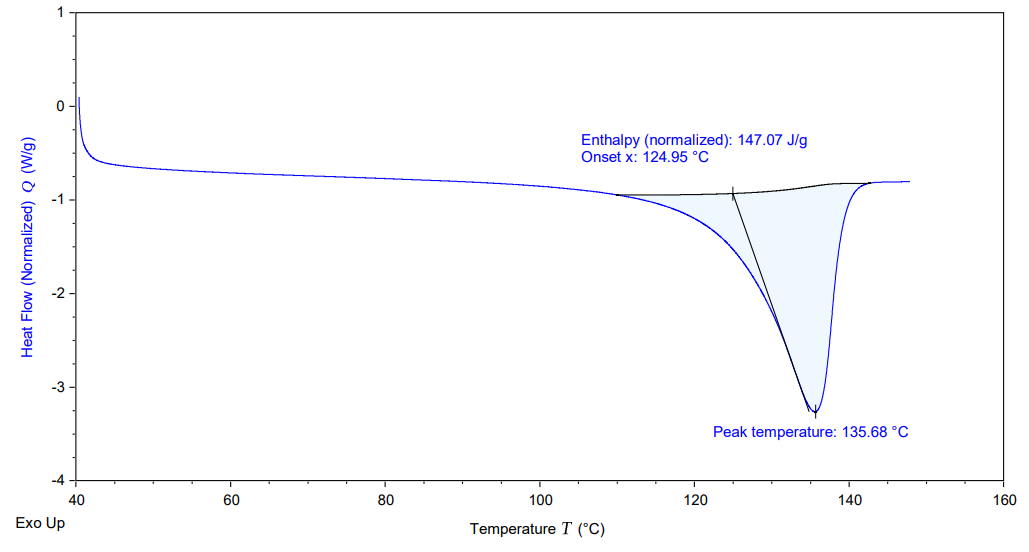


**Supplementary Figure 29.** DSC of the polymer from Supplementary Table 1, Entry 8.


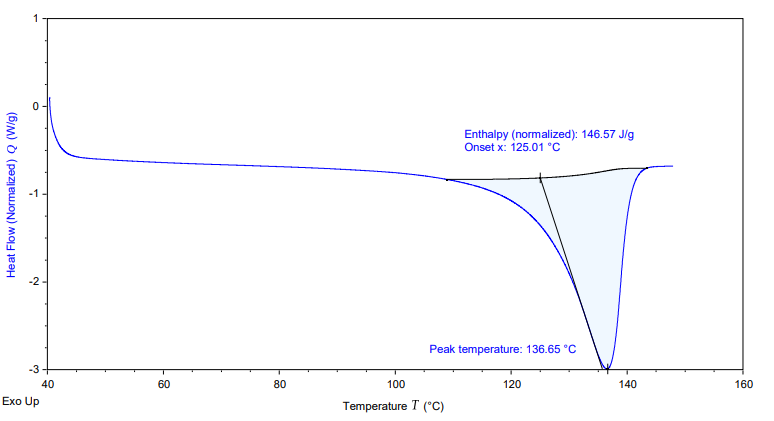


**Supplementary Figure 30.** DSC of the polymer from Supplementary Table 1, Entry 9.


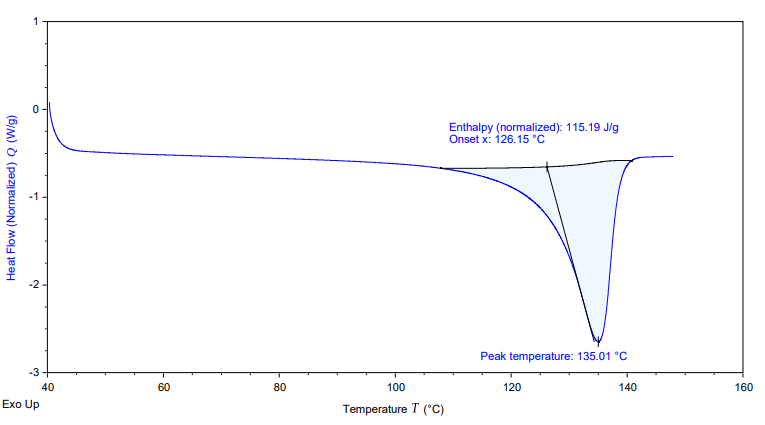


**Supplementary Figure 31.** DSC of the polymer from Supplementary Table 1, Entry 10.


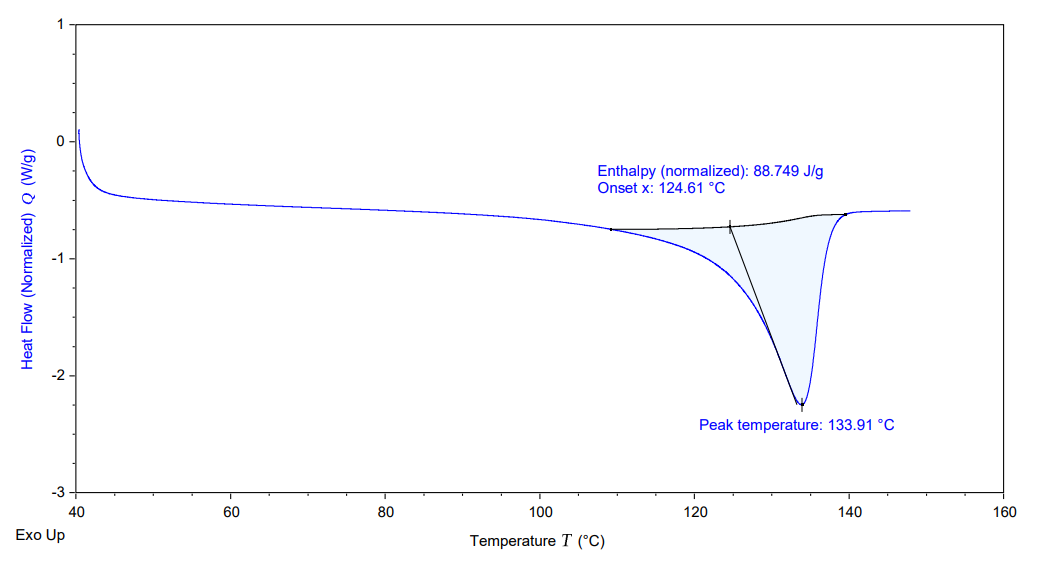


**Supplementary Figure 32.** DSC of the polymer from Supplementary Table 1, Entry 11.


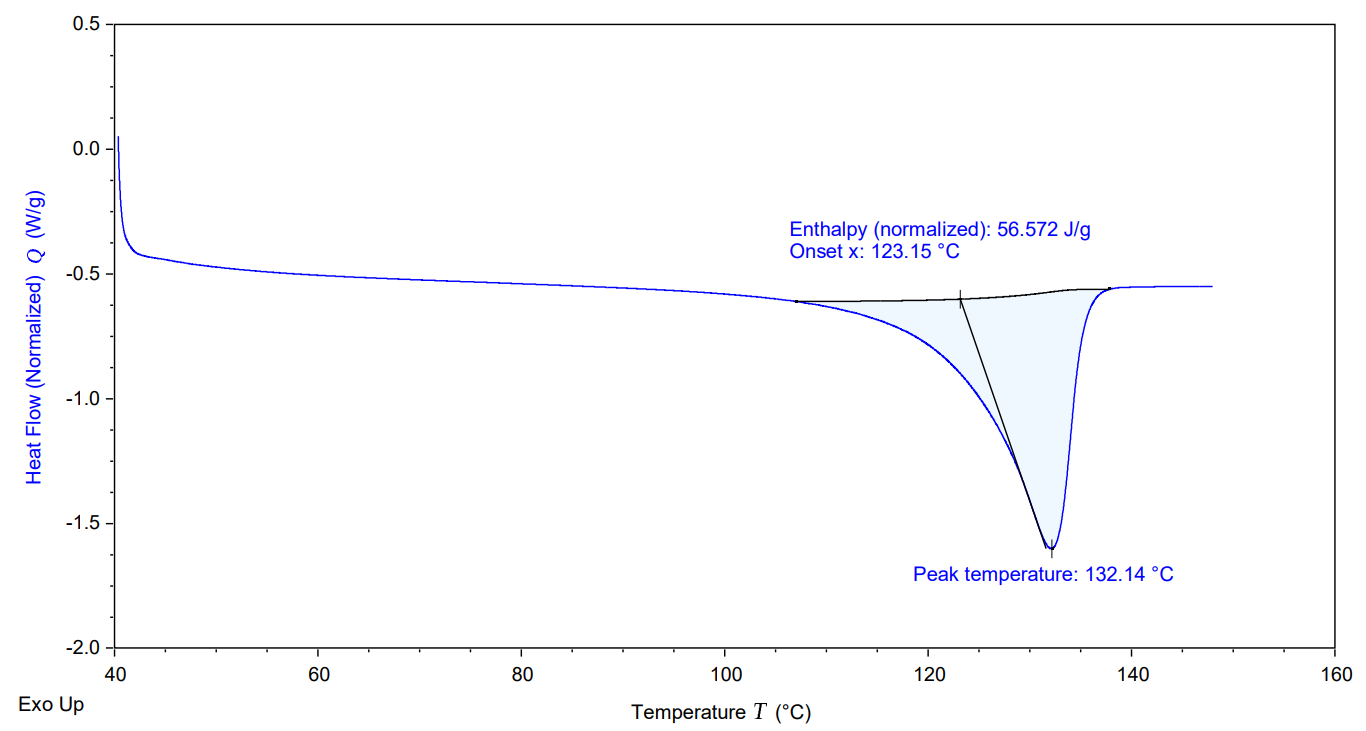


**Supplementary Figure 33.** DSC of the polymer from Supplementary Table 1, Entry 12.


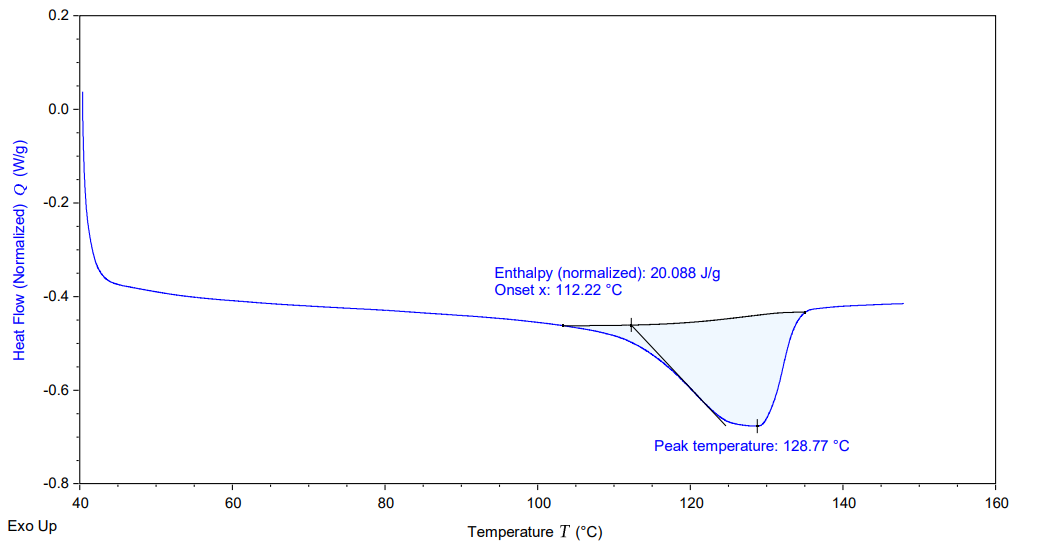


**Supplementary Figure 34.** DSC of the polymer from Supplementary Table 1, Entry 13.


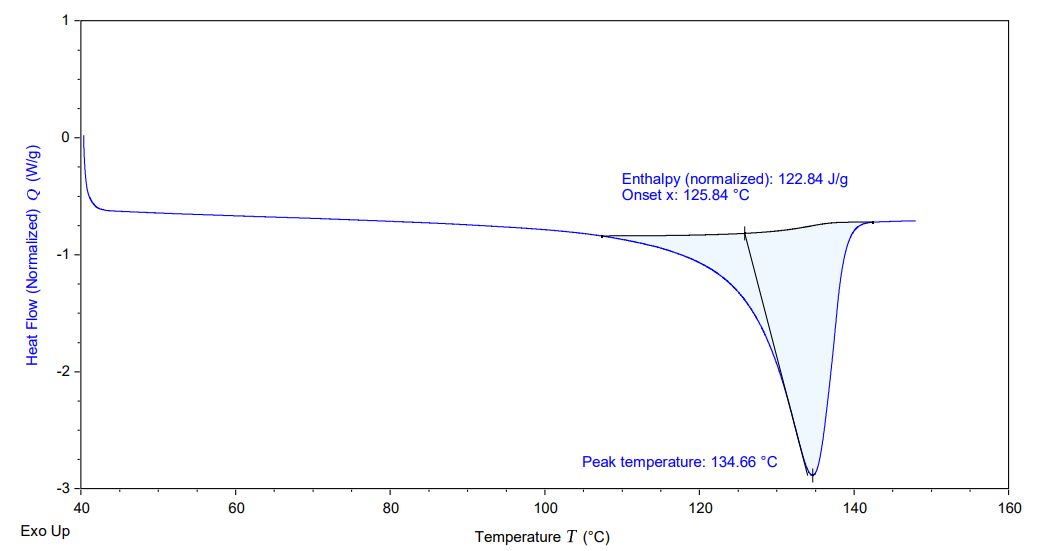


**Supplementary Figure 35.** DSC of the polymer from Supplementary Table 1, Entry 14.


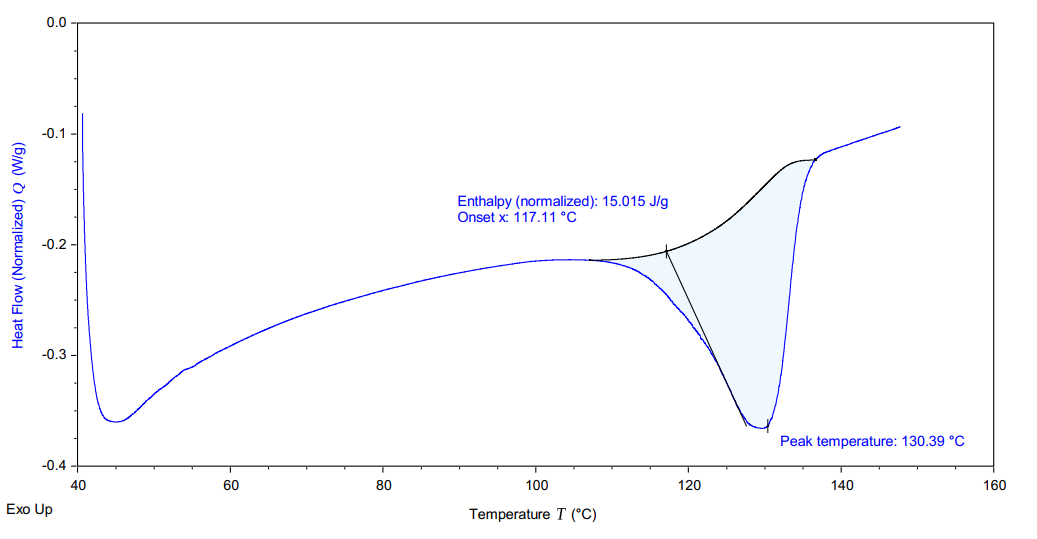


**Supplementary Figure 36.** DSC of the polymer from Supplementary Table 1, Entry 15.


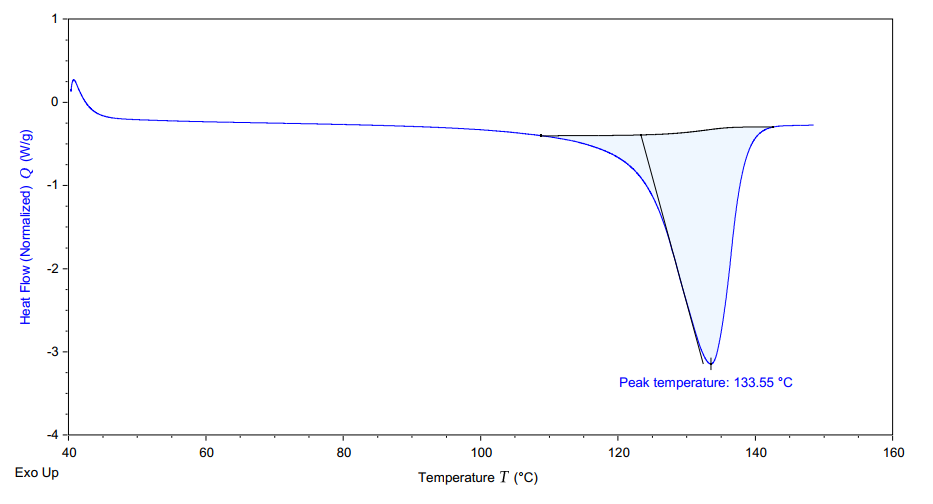


**Supplementary Figure 37.** DSC of the polymer from Supplementary Table 1, Entry 16.


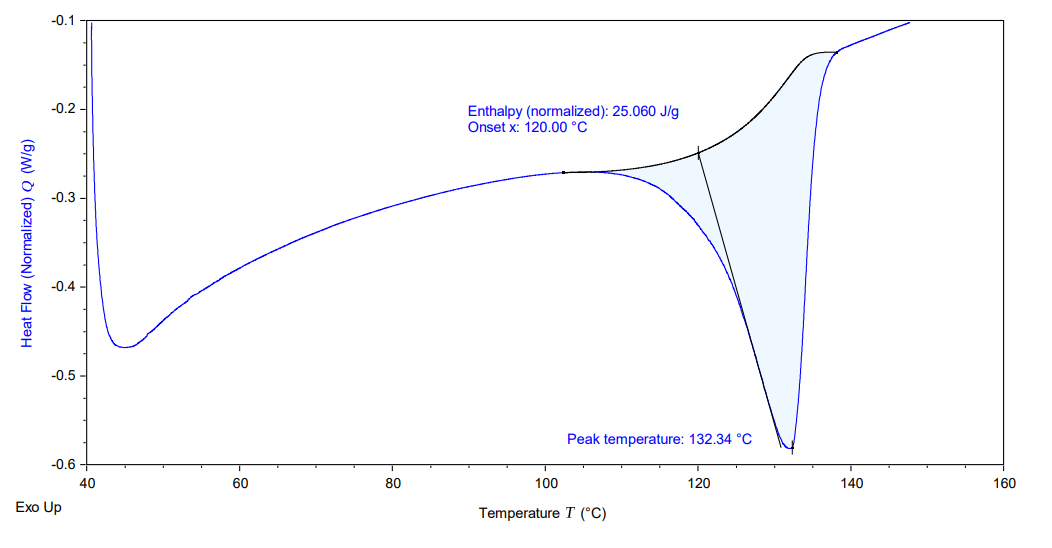


**Supplementary Figure 38.** DSC of the polymer from Supplementary Table 3, Entry 1.


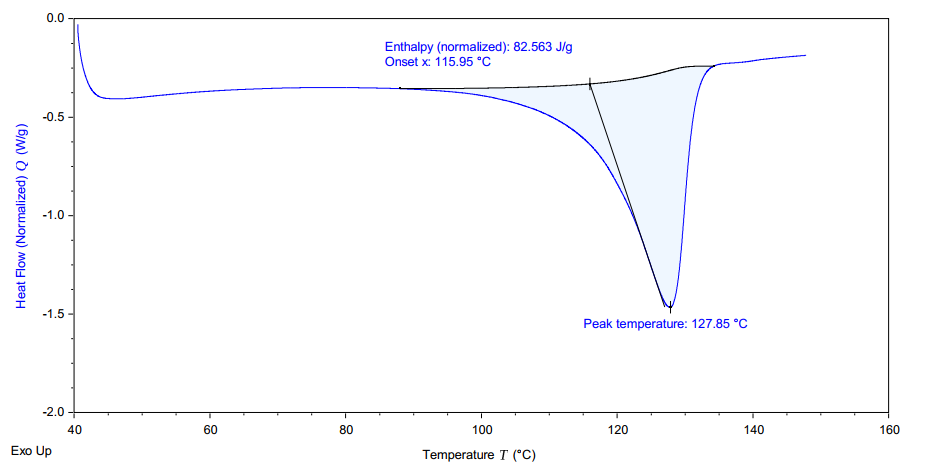


**Supplementary Figure 39.** DSC of the polymer from Supplementary Table 3, Entry 2.


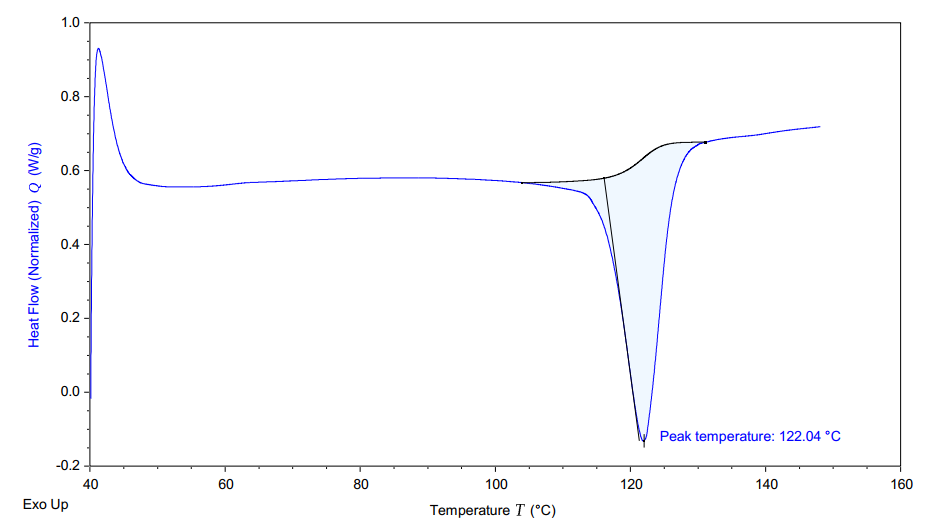


**Supplementary Figure 40.** DSC of the polymer from Supplementary Table 3, Entry 3.


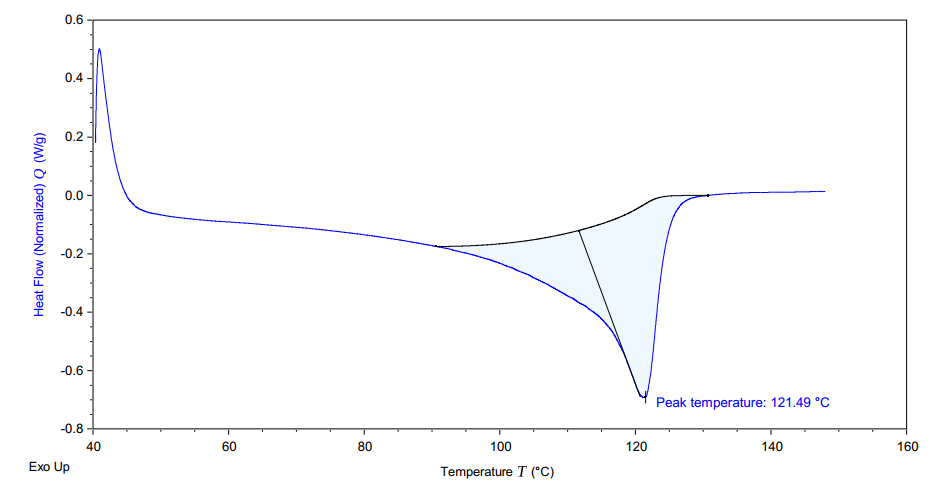


**Supplementary Figure 41.** DSC of the polymer from Supplementary Table 4, Entry 5.


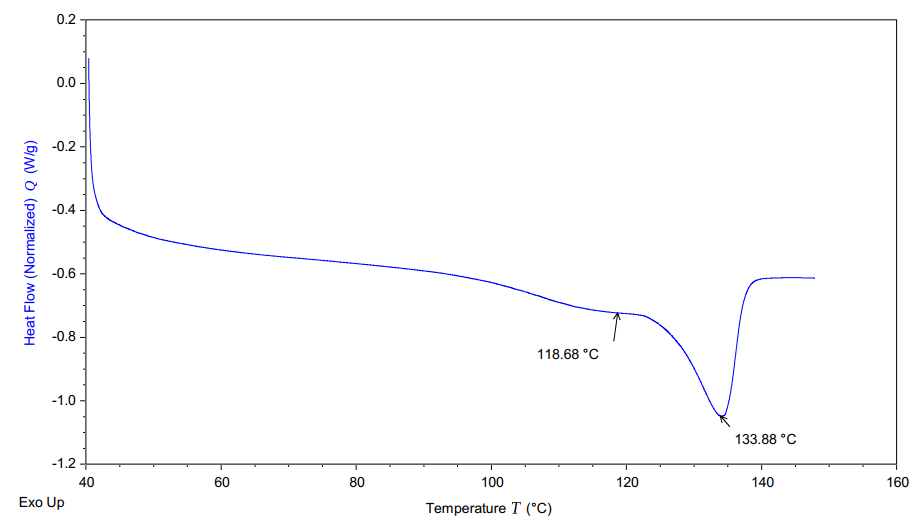


**Supplementary Figure 42.** DSC of the polymer from Table 1, Entry 21, prepared by mixed heterogeneous catalyst **Ni2-MgO/Ni3-MgO**.


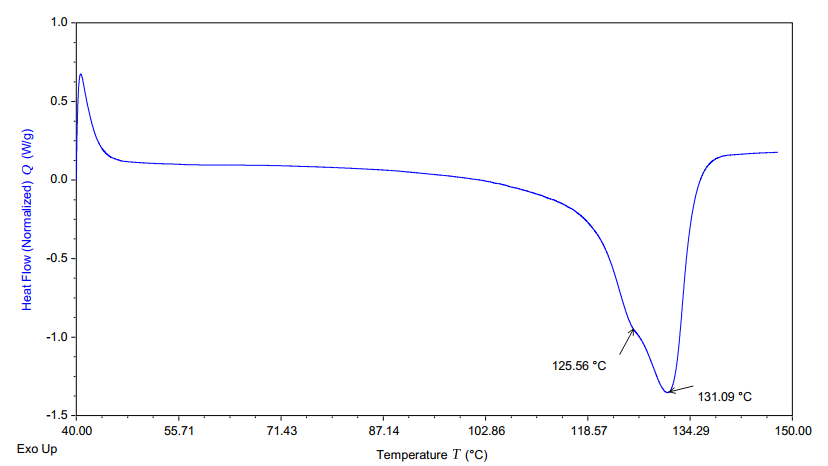


**Supplementary Figure 43.** DSC of the polymer from Table 1, Entry 22, prepared by mixed heterogeneous catalyst **Ni2-MgO/Ni3-MgO**.

**
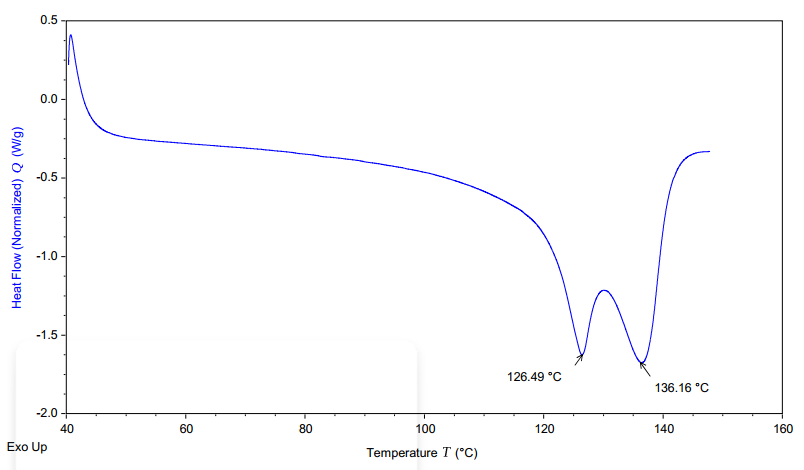
**

**Supplementary Figure 44.** DSC of the polymer from Supplementary Table 1, Entry 17, prepared by mixed homogeneous catalyst **Ni1/Ni3**.


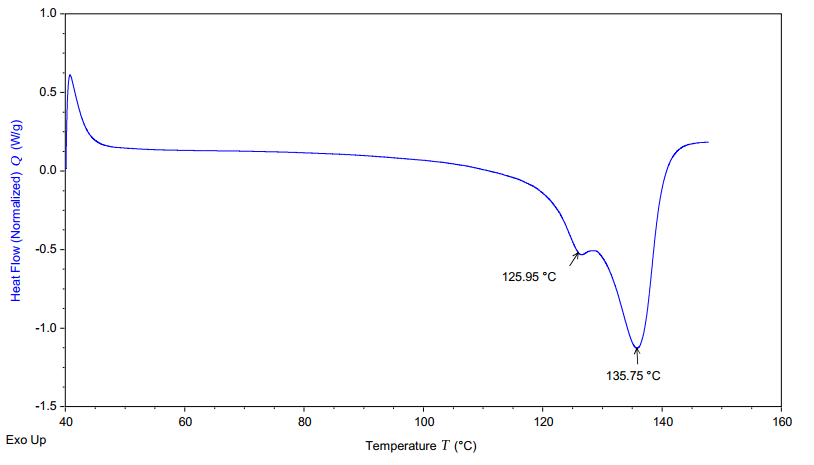


**Supplementary Figure 45.** DSC of the polymer from Supplementary Table 1, Entry 18, prepared by mixed heterogeneous catalyst **Ni2-MgO/Ni3-MgO**.


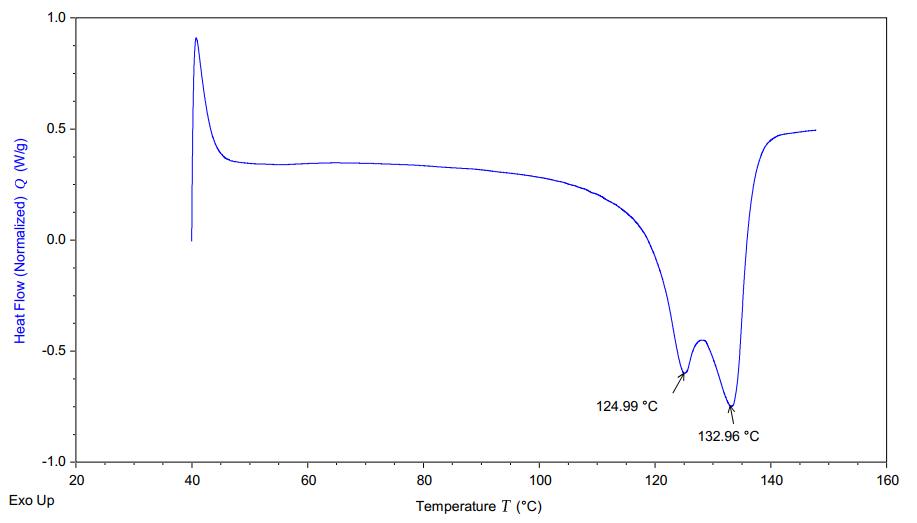


**Supplementary Figure 46.** DSC of the polymer from Supplementary Table 3, Entry 4, prepared by mixed homogeneous catalyst **Ni1/Ni3**.


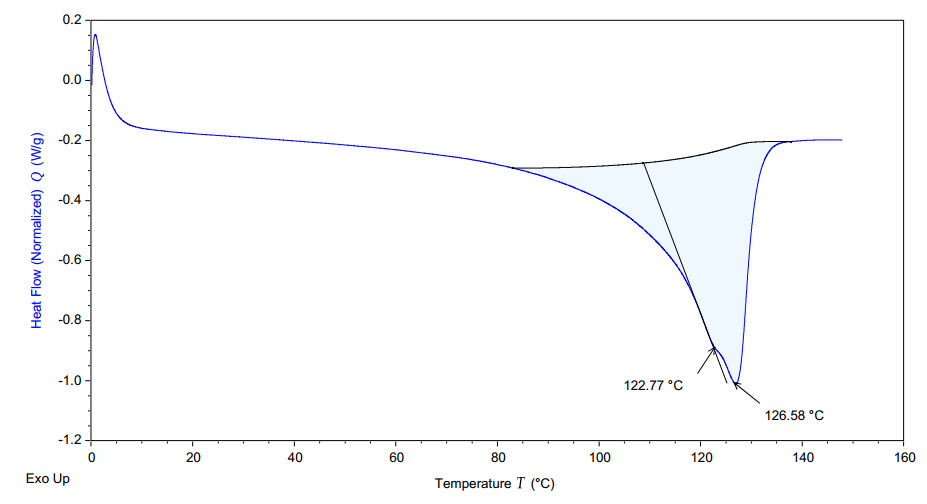


**Supplementary Figure 47.** DSC of the polymer from Supplementary Table 4, Entry 8, prepared by mixed heterogeneous catalyst **Ni2-MgO/Ni3-MgO**.


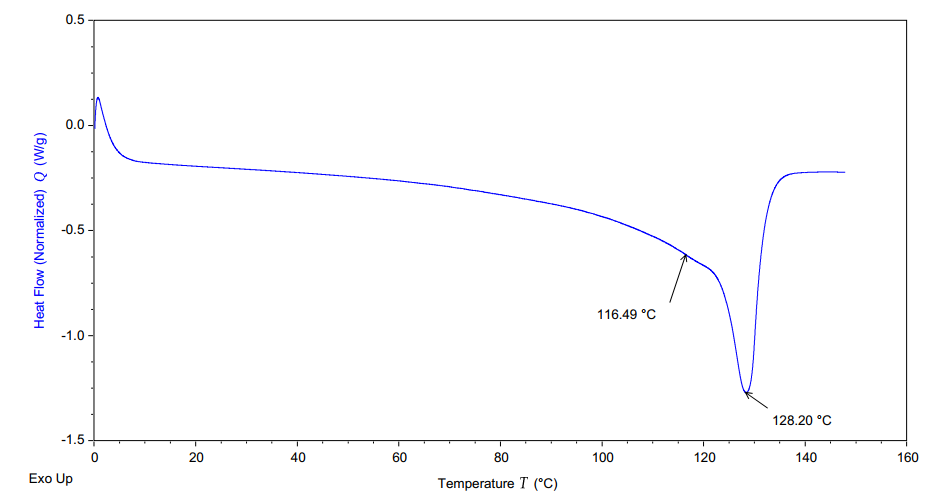


**Supplementary Figure 48.** DSC of the polymer from Supplementary Table 4, Entry 9, prepared by mixed heterogeneous catalyst **Ni2-MgO/Ni3-MgO**.


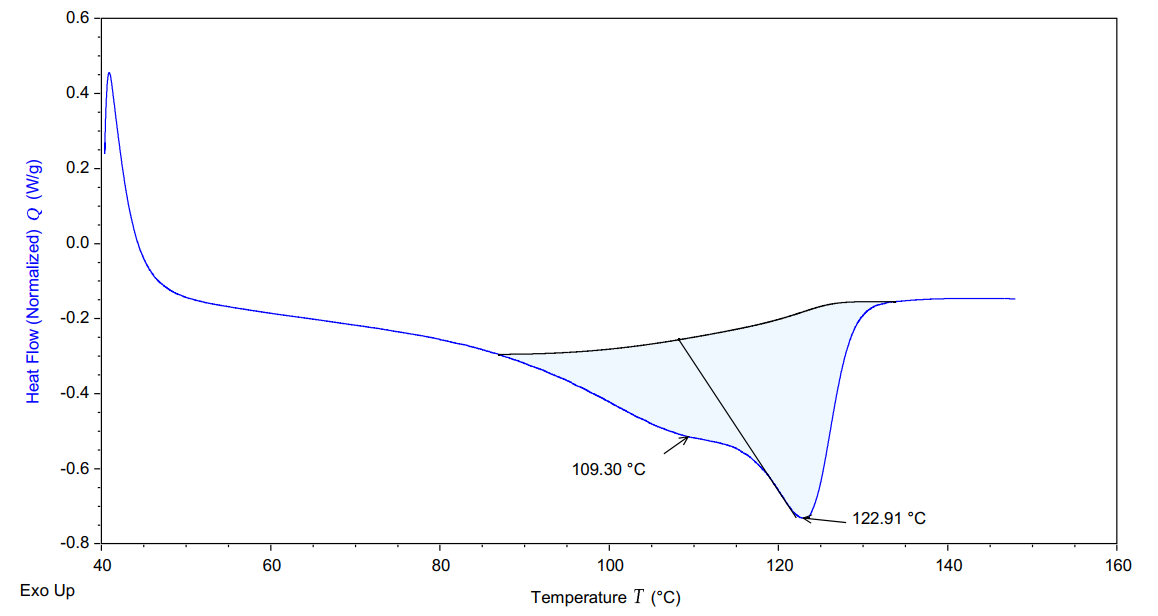


**Supplementary Figure 49.** DSC of the polymer from Supplementary Table 4, Entry 10, prepared by mixed heterogeneous catalyst **Ni2-MgO/Ni3-MgO**.

**
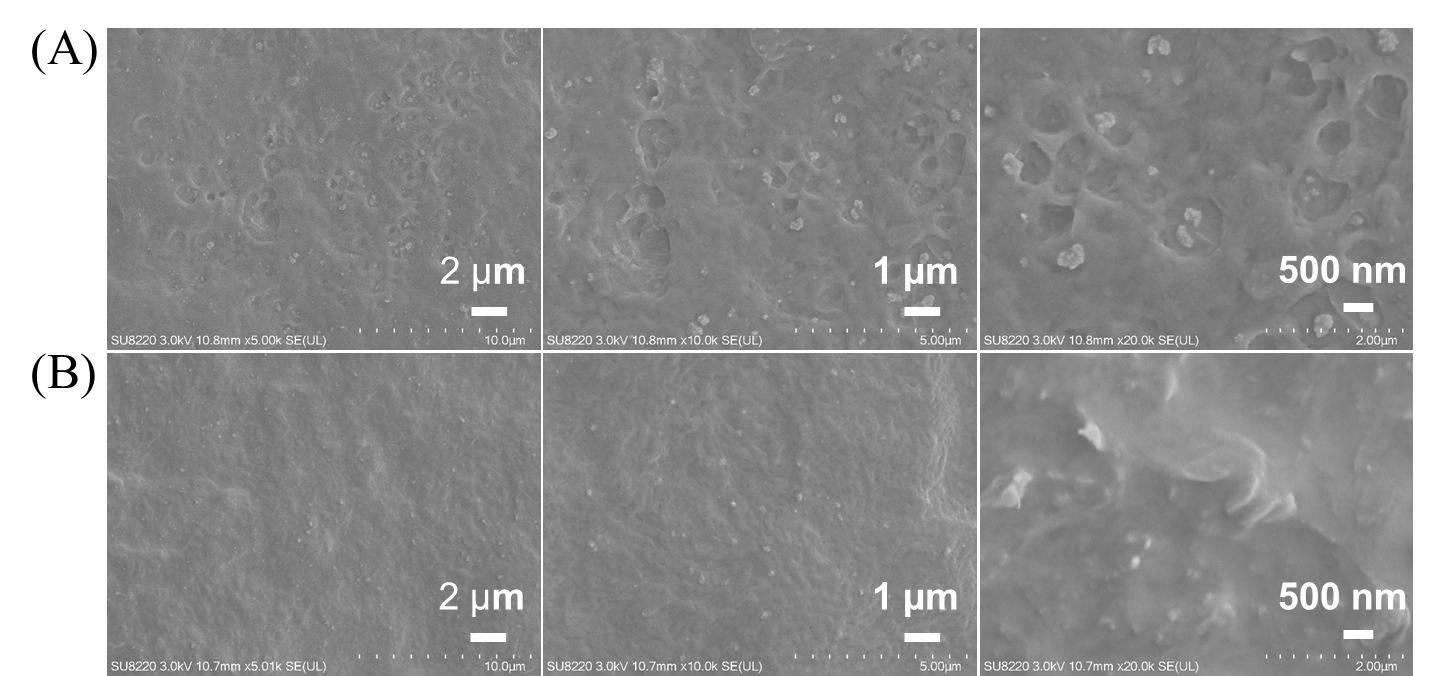
**

**Supplementary Figure 50.** (A) SEM image of PPE-**Ni2-MgO/Ni3-MgO** (prepared by mixed catalyst **Ni2-MgO** and **Ni3-MgO**). (B) SEM image of PPE-**Ni2/Ni3-MgO** (prepared by co-anchored heterogeneous catalyst **Ni2/Ni3-MgO**).


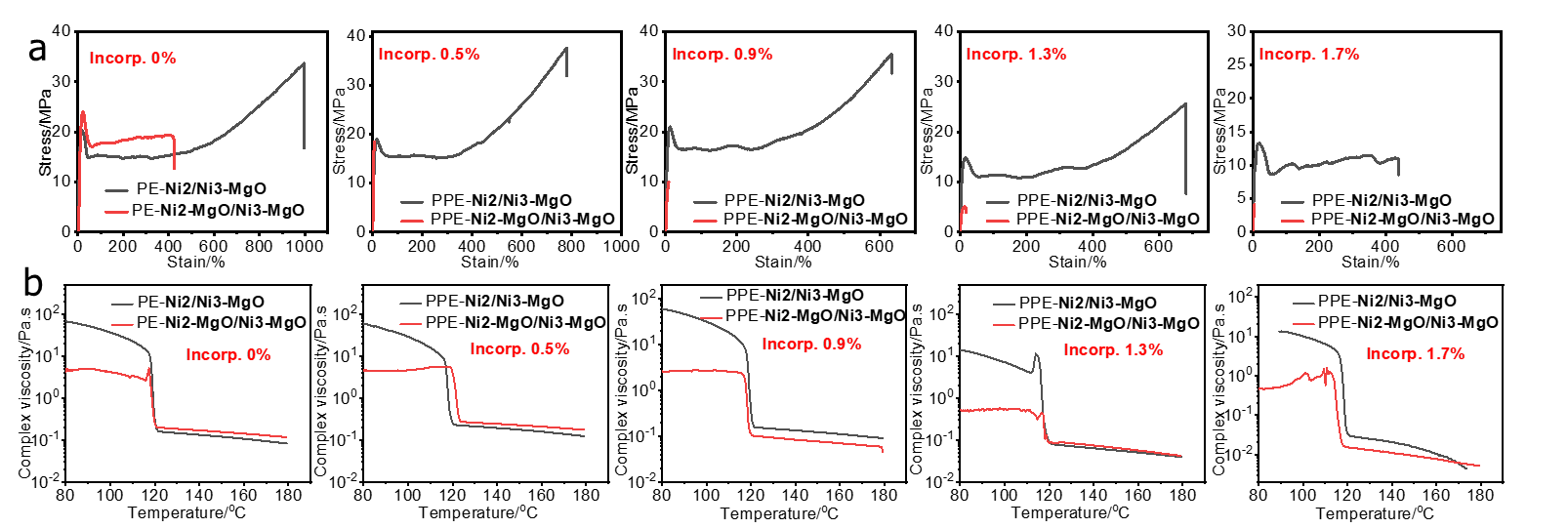


**Supplementary Figure 51.** Tensile curve (a) and complex viscosity curve (b) of a series of bimodal polyethylene.


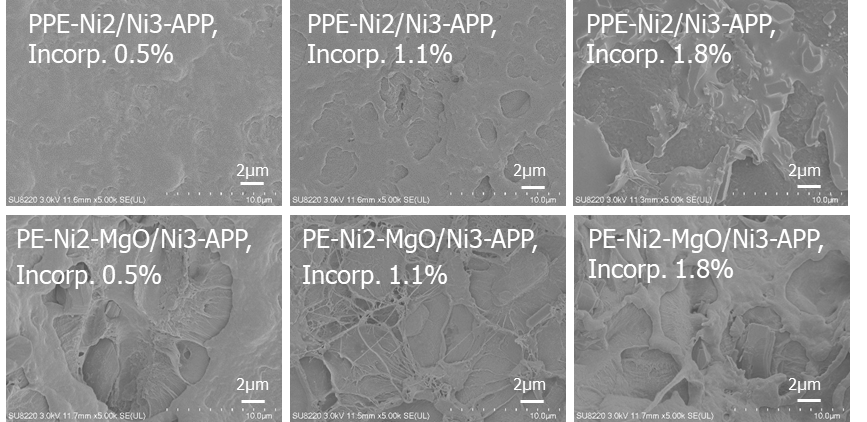


**Supplementary Figure 52.** SEM of a series of bimodal polyethylene prepared by APP supported catalyst. The upper polymers were prepared by co-anchoring strategy, and the lower polymers were prepared by mixed heterogeneous catalyst.


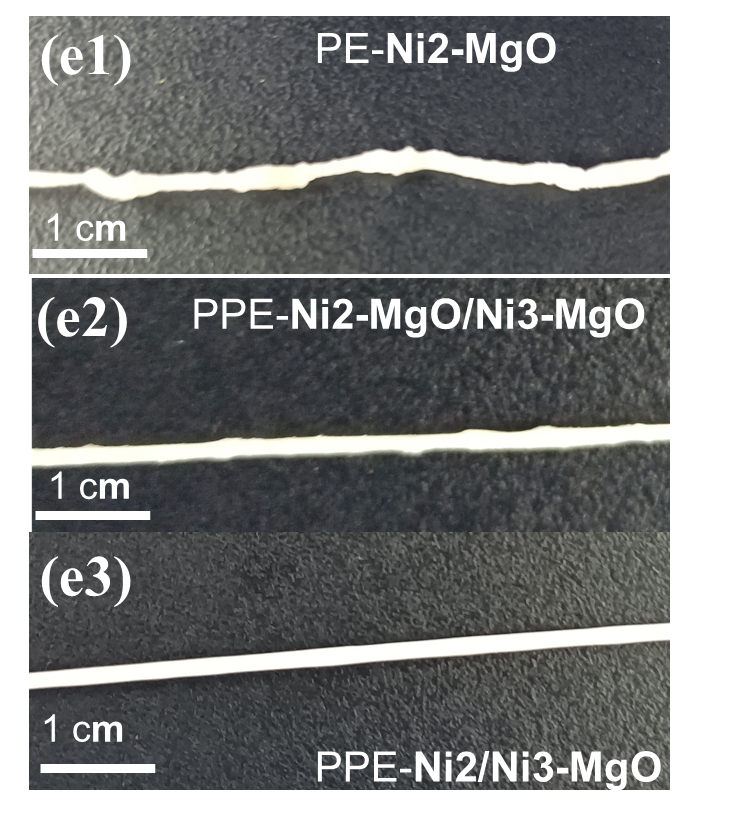


**Supplementary Figure 53.** Images of extruded samples of PE-**Ni2-MgO** (e1, Supplementary Table 1, Entry 5), PPE-**Ni2-MgO/Ni3-MgO** (e2, Table 1, Entry 22) and PPE-**Ni2/Ni3-MgO** (e3, Table 1, Entry 12).


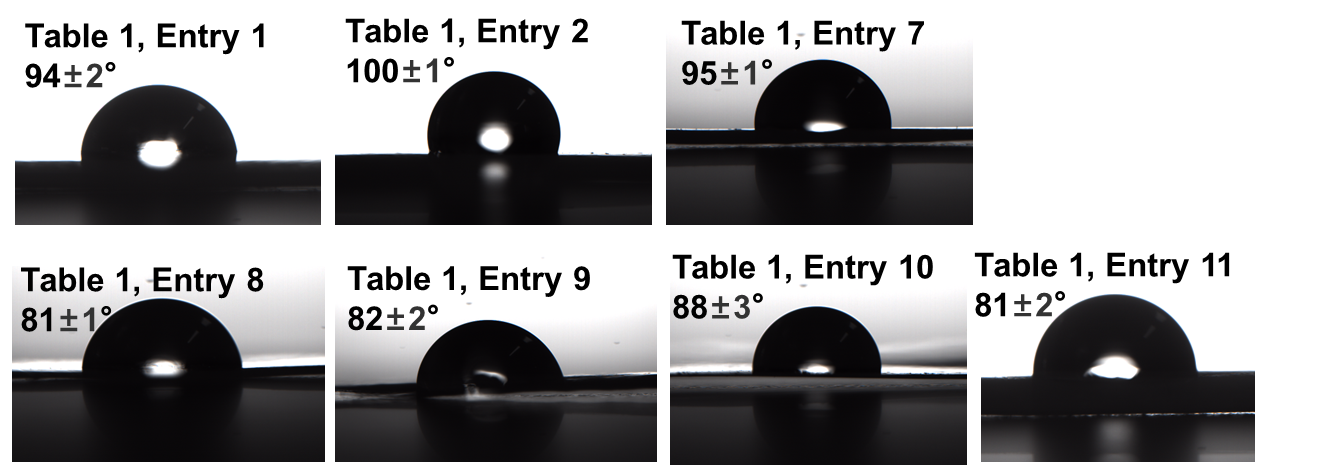


**Supplementary Figure 54.** Pictures of water contact angle measurement.


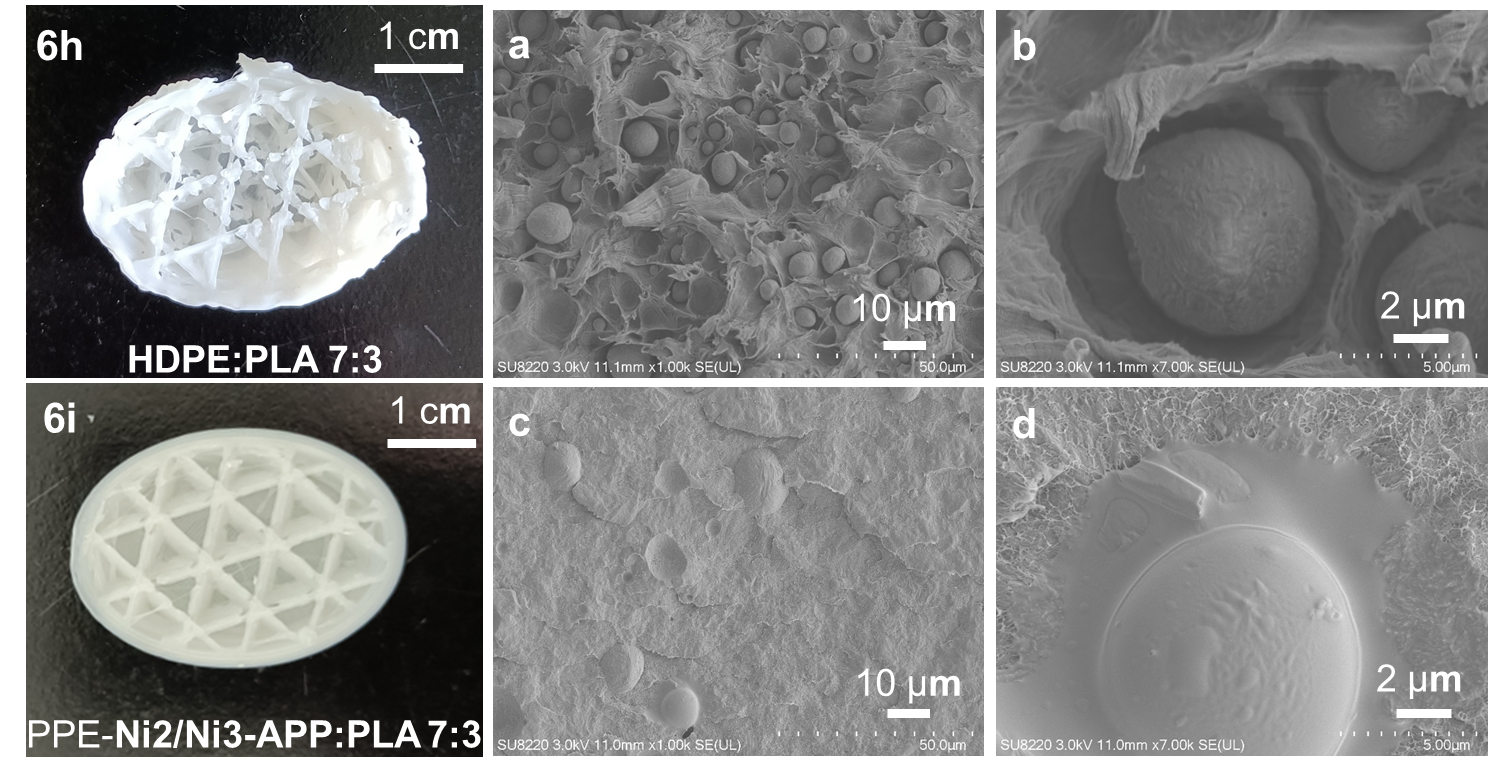


**Supplementary Figure 55.** SEM data for products in Figure 6H and 6I in the manuscript. (a) and (b), SEM for HDPE: PLA 7:3 commercial (prepared by blending HDPE and polylactic acid in a ratio of 7 to 3). (c) and (d), SEM for PPE-**Ni2/Ni3-APP**: PLA 7:3 (prepared by PPE-**Ni2/Ni3-APP** and polylactic acid in a ratio of 7 to 3).


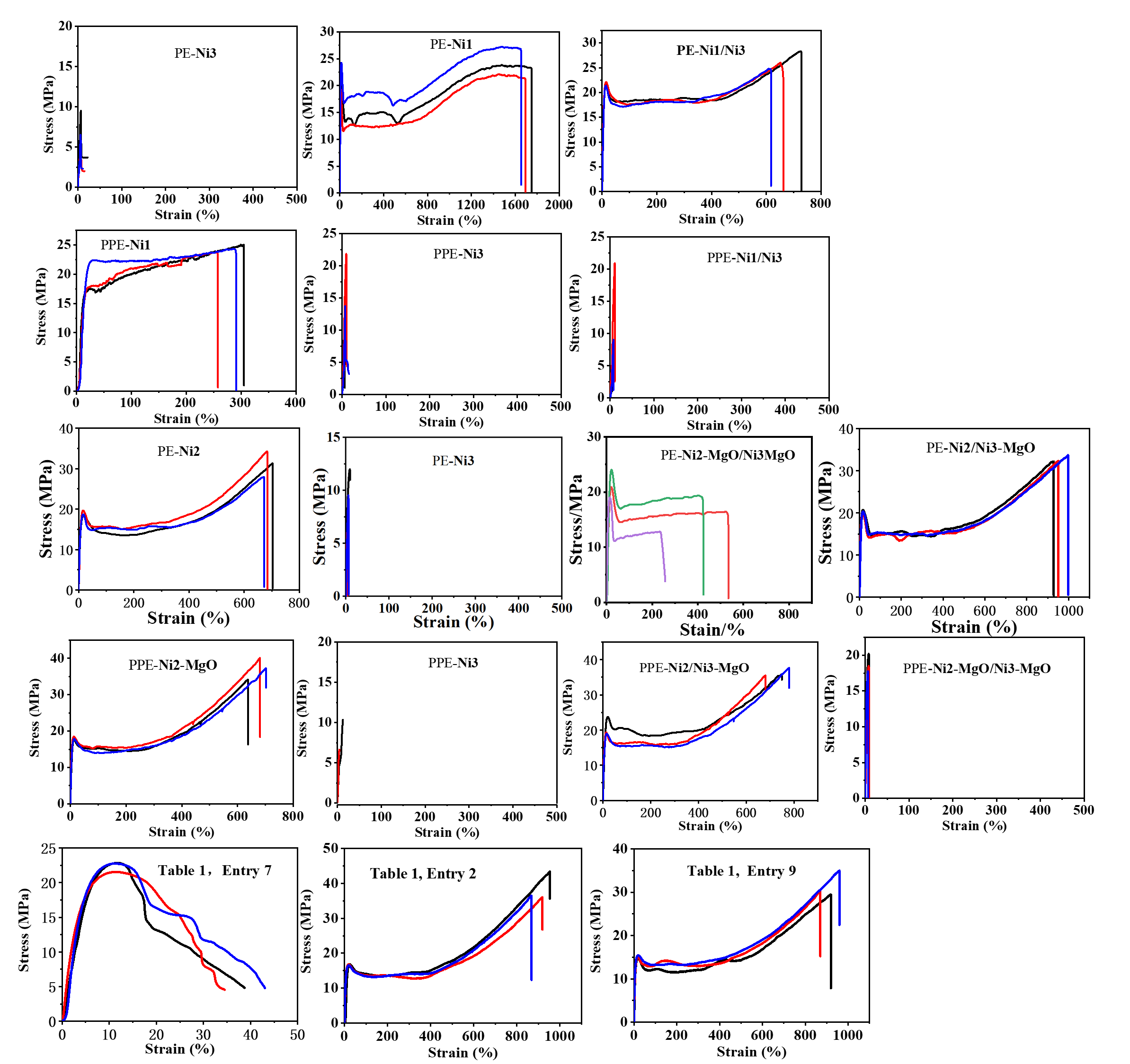


**Supplementary Figure 56.** Original data of stress-strain curve.

**2. Supplementary Methods**

**Preparation of homogeneous catalysts.**

**Ni1**: Under nitrogen, 2,2'-((2-(tert-butyl)-1,4-phenylene)bis(oxy))bis(tetrahydro-2H-pyran)(3.34g,10 mmol) was dissolved in 50 mL of dried THF. n-BuLi (4.6mL,2.4 M in hexane,11 mmol, 1.1 equiv.) was added dropwise and the reaction mixture was stirred for 2 h at 0 ^o^C. The flask was transferred to a -78 ^o^C bath, and a THF solution of chloro(2',6'-dimethoxy-[1,1'-biphenyl]-2-yl)(phenyl)phosphane (11 mmol,1.1 equiv) was added dropwise. The mixture was stirred for 1 hour, and warmed to room temperature to react for 12 h. After quenching, the suspension was transferred to a round bottom flask and the THF was evaporated on a rotary evaporator. The crude material was extracted with CH_2_Cl_2_ (3 x 200 mL), washed with H_2_O (3 x 100 mL), and the organic phase was collect and dried over Na_2_SO_4_. After filtration and concentration, the crude product of the protected ligand can be obtained, which can be directly used for the next reaction without further purification. The protected ligand was dissolved in methanol under nitrogen, 3 equiv. of p-toluenesulfonic acid was added to react for 6 hours. The reaction solution was evaporated with a rotary evaporator to evaporate methanol. The crude material was extracted with CH_2_Cl_2_ (3 x 200 mL), washed with H_2_O (3 x 100 mL), and the organic phase was collected and dried over Na_2_SO_4_. After filtration and concentration, pure **ligand L1** was obtained by column chromatography as a white solid (2.19 g, 45%). Under nitrogen, a toluene solution of 1 mmol (487 mg) **ligand L1** and 1.2 eq. Py_2_NiMe_2_ was stirred for 1 hour at room temperature. After concentrating, recrystallization with n-hexane resulted in the formation of **Ni1** (543 mg, 85%) as a yellow solid. **ligand** **L1**: ^1^H NMR (600 MHz, Chloroform-*d*) δ 7.41 (dd, *J* = 7.5, 1.5 Hz,1H), 7.29-7.24(m, 5H), 7.24-7.16(m, 4H)6.76 (d, *J* = 3.0 Hz, 1H), 6.51 (dd, *J* = 8.3, 4.3 Hz, 2H), 6.24 (dd, *J* = 4.2, 3.0 Hz, 1H), 6.16 (d, *J* = 10.5 Hz, 1H), 4.41 (s, 1H), 3.51 (s, 3H, *-MeO*), 3.45 (s, 3H, *-MeO*), 1.32 (s, 9H, *-tBu*).^31^P NMR (243 MHz, CDCl_3_) δ -37.22.^13^C NMR (151 MHz, Chloroform-*d*) δ 157.75, 157.71, 152.29, 152.15, 148.32, 141.42, 141.21, 137.48, 135.97, 133.66, 133.54, 133.49, 131.14, 131.10, 129.60, 129.55, 128.46, 128.33, 128.29, 127.65, 122.03, 118.57, 117.37, 116.43, 103.92, 103.82, 55.66, 55.46, 34.93, 29.55. ESI-MS (m/z): [M+H]^+^ Calcd for C30 H32 O4 P, 487.20327; Found: 487.20386. **Ni1**: ^1^H NMR (400 MHz, C_6_D_6_) δ 8.11 (s, 2H), 7.30 (s, 2H), 7.23-7.09 (m, 1H), 6.91 (dt, *J* = 14.1, 6.6 Hz, 2H), 6.78 - 6.61 (m, 5H), 6.53 (s, 2H), 6.42 - 6.19 (m, 4H), 6.07 (d, *J* = 8.2 Hz, 1H), 3.09 (s, 3H), 2.73 (s, 3H), 1.15 (s, 9H, *-tBu*), -1.12 (s, 3H, Ni-*Me*). ^31^P NMR (162 MHz, C_6_D_6_) δ 22.62. ^13^C NMR (101 MHz, C_6_D_6_) δ 159.51, 158.03, 150.50, 141.41, 141.30, 137.58, 136.71, 135.84, 134.82, 132.91, 132.35, 132.29, 129.48, 128.92, 128.04, 127.92, 127.76, 127.60, 126.33, 122.55, 119.90, 114.72, 103.08, 102.91, 54.66, 54.19, 34.85, 29.57, -14.72 (d, *J* = 36.6 Hz, Ni-*Me*). Anal. Calcd for C36H38NNiO4P: C, 67.73; H, 6.00; N, 2.19. Found: C, 67.52; H, 6.11; N, 2.25. The characterization results of **Ni1** are consistent with those reported in literature.^[1]^

**Ni2**: Under nitrogen, 2-(2-tert-butylphenoxy)-tetrahydro-2H-pyran (2.34 g, 10 mmol) was dissolved in 50 mL dried THF. n-BuLi (4.6 mL, 2.4 M in hexane, 11 mmol, 1.1 equiv.) was added dropwise and the reaction mixture was stirred for 2 h at 0 ^o^C. The flask was transferred to a -78 ^o^C bath, and a THF solution of chloro(2',6'-dimethoxy-[1,1'-biphenyl]-2-yl)(4-((tetrahydro-2H-pyran-2-yl)oxy)phenyl)phosphane (11 mmol,1.1 equiv) was added dropwise. The mixture was stirred for 1 hour, and warmed to room temperature to react for 12 hours. After quenching, the suspension was transferred to a round bottom flask and the THF was evaporated on a rotary evaporator. The crude product was extracted with CH_2_Cl_2_ (3 x 200 mL), washed with H_2_O (3 x 100 mL), and the organic phase was collected and dried over Na_2_SO_4_. After filtration and concentration, the crude product of the protected ligand can be obtained, which was used for the next reaction without further purification. The protected ligand was dissolved in methanol under nitrogen, and 3. eq of p-toluenesulfonic acid was added to react for 6 hours. The reaction solution was evaporated with a rotary evaporator to evaporate methanol. The crude material was extracted with CH_2_Cl_2_ (3 x 200 mL), washed with H_2_O (3 x 100 mL), and the organic phase was collected and dried over Na_2_SO_4_. After filtration and concentration, the pure **ligand L2** was obtained by column chromatography as a white solid (2.59 g, 53%). Under nitrogen, a toluene solution of 1 mmol (486 mg) **ligand L2** and 1.2 eq. Py_2_NiMe_2_ was stirred for 1 hour at room temperature. After concentration, the addition of n-hexane for recrystallization resulted in the formation of **Ni2** (510 mg, 80%) as a yellow solid. **ligand** **L2**: ^1^H NMR (400 MHz, Chloroform-*d*) δ 7.42 (ddd, *J* = 7.5, 3.7, 2.0 Hz, 1H), 7.29 (ddd, *J* = 8.2, 5.8, 2.2 Hz, 2H), 7.25-7.17 (m, 3H), 7.08 (ddd, *J* = 11.0, 7.2, 2.6 Hz, 2H), 6.89-6.80 (m, 1H), 6.82 -6.73 (m, 1H), 6.71 (ddt, *J* = 8.7, 3.1, 1.5 Hz, 2H), 6.54 (ddt, *J* = 13.6, 8.5, 2.3 Hz, 3H), 5.40 (s, 1H,-O*H*), 3.61 (s, 3H, -O*Me*), 3.40 (s, 3H,-O*Me*), 1.45 (s, 9H, *tBu*). ^13^C NMR (101 MHz, CDCl_3_) δ 157.96, 157.76, 157.67, 157.60, 156.20, 141.09, 140.78, 136.27, 135.52, 135.31, 133.23, 132.30, 130.95, 130.89, 129.44, 129.18, 127.98, 127.50, 125.54, 122.11, 119.72, 118.72, 115.54, 115.46, 103.97, 103.78, 55.69, 55.38, 34.80, 29.61. ^31^P NMR (162 MHz, CDCl_3_) δ -40.71. **Ni2**: ^1^H NMR (400 MHz, C_6_D_6_) δ 8.33 (s, 1H, *Py*), 7.57 (s, 1H), 7.31 (s, 2H), 7.05 (s, 3H), 6.86 (s, 3H), 6.57-6.70 (m, 4H), 6.41 (s, 2H), 6.31 (m, 2H), 6.22 (m, 2H), 3.25 (s, 3H, -O*Me*), 2.86 (s, 3H, -O*Me*), 1.37 (s, 9H, *tBu*), -0.80 (s, 3H, Ni*Me*). ^31^P NMR (162 MHz, C_6_D_6_) δ 20.8. ^13^C NMR (101 MHz, C_6_D_6_) δ 174.20, 174.07, 168.19, 159.17, 158.05, 150.60, 149.66, 141.09, 140.99, 136.69, 136.31, 135.75, 134.58, 134.20, 132.87, 130.52, 129.08, 128.77, 128.02, 127.90, 127.74, 127.57, 126.00, 123.78, 122.59, 121.84, 121.50, 119.94, 112.34, 102.83, 54.64, 53.87, 34.78, 29.66, 1.09, -14.22(Ni*Me*). Anal. Calcd for C36H37NNiO4P: C, 67.84; H, 5.85; N, 2.20, Found: C, 67.58; H, 5.91; N, 2.18.

**Ni3**: Our initial attempt to react ligand **L3** with (Py)_2_NiMe_2_ failed to give any isolable product. It may be due to the side reaction of this nickel precursor with the para-hydroxy group of ligand **L3** with small steric hindrance. So we employed an alternative synthetic strategy for Ni3 according to the reference (J. Am. Chem. Soc. 2017, 139, 3611-3614). Under nitrogen, 2,2'-((2-(tert-butyl)-1,4-phenylene)bis(oxy))bis(tetrahydro-2H-pyran)(3.34g, 10 mmol) was dissolved in 50 mL of dried THF. n-BuLi(4.6mL, 2.4 M in hexane, 11 mmol, 1.1 equiv) was added dropwise and the reaction mixture was stirred for 2 h at 0 ^o^C. The flask was transferred to a -78 ^o^C bath, and the THF solution of chlorodiphenylphosphane (11 mmol, 1.1 equiv) was added dropwise. The mixture was stirred for 1 hour, and warmed to room temperature to react for 12 hours. After quenching, the suspension was transferred to a round bottom flask and the THF was evaporated on a rotary evaporator. The crude material was then extracted with DCM (3 x 200 mL), washed with H_2_O (3 x 100 mL), and then collect the organic phase and dried with Na_2_SO_4_. After filtration and concentration, the crude product of the protected ligand can be obtained, which can be directly used for the next reaction without further purification. Then, the protected ligand was dissolved in methanol under nitrogen, 3. eq of p-toluenesulfonic acid was added to react for 6 hours. Then, the reaction solution was evaporated with a rotary evaporator to evaporate methanol, the crude material was then extracted with DCM (3 x 200 mL), washed with H_2_O (3 x 100 mL), and then collect the organic phase and dried with Na_2_SO_4_. After filtration and concentration, the pure **ligand L3** was obtained by column chromatography as a white solid (1.80 g, 51%). Under nitrogen, a toluene solution of 1 mmol (350 mg) of the ligand and 1.0 eq. Ni(COD)_2_ was stirred for 1 hour at room temperature , and then drain to obtain catalyst **Ni3** (470 mg, 93%) as a yellow solid. **ligand L3**: ^1^H NMR (400 MHz, Chloroform-*d*) δ 8.33 (s, 1H), 7.37-7.29 (m, 10H, -*Ph*), 7.31 (s, 1H), 6.80 (s, 1H), 6.46 (s, 1H), 6.23 (s, 1H), 4.46 (s, 1H, -*OH*), 1.38 (s, 9H, *-tBu*). ^31^P NMR (162 MHz, C_6_D_6_) δ -30.01.^13^C NMR (101 MHz, CDCl_3_) δ 152.36, 152.16, 148.59, 148.56, 138.07, 138.06, 134.84, 133.57, 133.39, 129.12, 128.80, 128.73, 121.86, 117.31, 117.29, 117.27, 117.21, 77.44, 77.33, 77.13, 76.81, 35.01, 34.99, 29.53. **Ni3**: ^1^H NMR (400 MHz, C_6_D_6_) δ 7.31 (ddd, *J* = 30.1, 10.9, 7.2 Hz, 4H), 6.91- 6.65 (m, 5H), 6.59(s, 1H), 6.45 (s, 1H), 6.20 - 6.07 (m, 1H), 5.66 -5.12 (m, 2H, *-CH=CH-*), 3.76 (s, 1H, *-OH*), 2.09 - 1.79 (m, 4H), 1.74 (q, 1H), 1.70 -1.60 (m, 1H), 1.56 (s, 1H), 1.45-1.39 (m,1H), 1.34 (s, 9H, *-tBu*), 1.19 - 1.07 (m, 2H), 0.80 - 0.69 (m, 1H); ^31^P NMR (162 MHz, C_6_D_6_) δ 16.96. ^13^C NMR (101 MHz, C_6_D6) δ 145.31, 138.47, 133.11, 132.25, 132.12, 130.17, 128.53, 127.64, 127.54, 125.23, 118.38, 113.84, 107.23, 34.49, 34.47, 29.75, 28.53, 27.21, 25.97, 22.46, 22.29. Anal. Calcd for C30H35NiO2P: C, 69.66; H, 6.82. Found: C, 69.52; H, 6.89.

**Preparation of heterogeneous catalysts Ni1-MgO**, **Ni2-MgO** and **Ni3-MgO.**

To a toluene solution of homogeneous nickel catalyst, 1.2 equiv. NaH was added and stirred for 1 hour to obtain ONa functionalized catalyst solution. To this solution, a suspended solid support in toluene was added dropwise and stirred for 1 h. The supported heterogeneous catalysts (**Ni1-MgO**, **Ni2-MgO** and **Ni3-MgO**) were filtered, dried under vacuum and re-suspended in heptane for polymerization. The loading content (1 μmol catalyst per 30 mg support) is less than maximum supporting capacity for all cases.

**Preparation of heterogeneous catalysts with the co-anchoring strategy.**

**Ni1/Ni2-MgO (1:1)**. Catalysts **Ni1** and **Ni2** (1:1) were dissolved in toluene. 1.2 equivalent NaH was added and stirred for 1 hour to obtain ONa functionalied catalyst solution. To this solution, a suspended solid support in toluene was added dropwise and stirred for 1 h. The co-anchored heterogeneous catalysts were filtered, dried under vacuum and re-suspended in heptane for polymerization. **Ni1/Ni3-MgO**, **Ni2/Ni3-MgO**, **Ni1/Ni2/Ni3-MgO**, **Ni2/Ni3-APP** (Ammonium polyphosphate), **Ni2/Ni3-GF** (Glass fiber), **Ni2/Ni3-TiO_2_**, **Ni2/Ni3-lignin** were all prepared according to the above method.

**
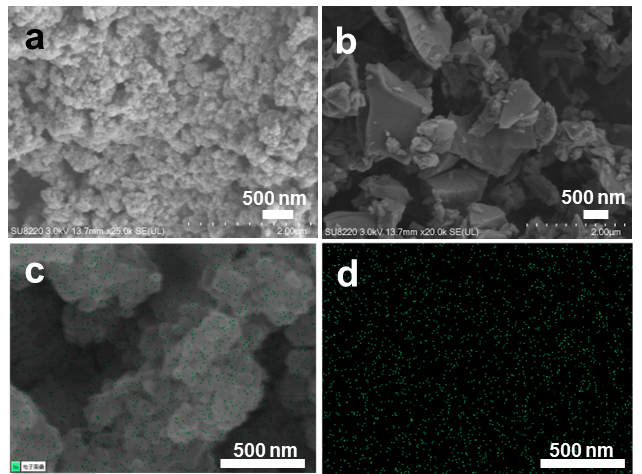
**

**Supplementary Figure 57**. (a) and (b) SEM images of heterogeneous catalysts **Ni1/Ni3-MgO (1:1)** and **Ni2/Ni3-GF (1:1)**. (c) and (d) Energy Dispersive Spectroscopy (EDS) of heterogeneous catalysts **Ni1/Ni3-MgO (1:1)**, green represents the distribution of nickel.

**3. Supplementary Figures of Characterization of ligands and homogeneous catalysts**


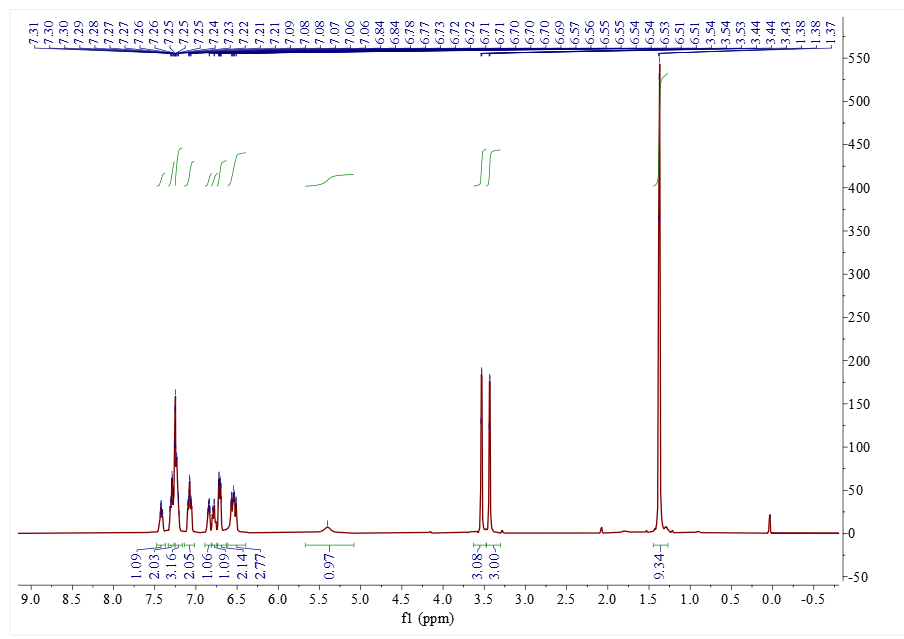


**Supplementary Figure 58**.^1^H NMR spectrum of the **ligand L2.** (CDCl_3_, 400 MHz)


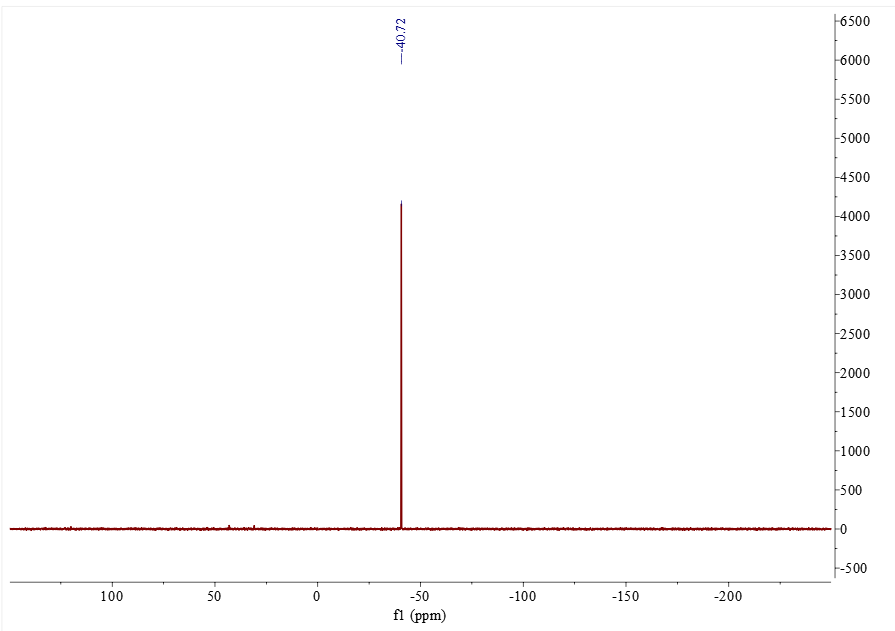


**Supplementary Figure 59**.^31^P NMR spectrum of the **ligand L2.** (CDCl_3_, 162 MHz)


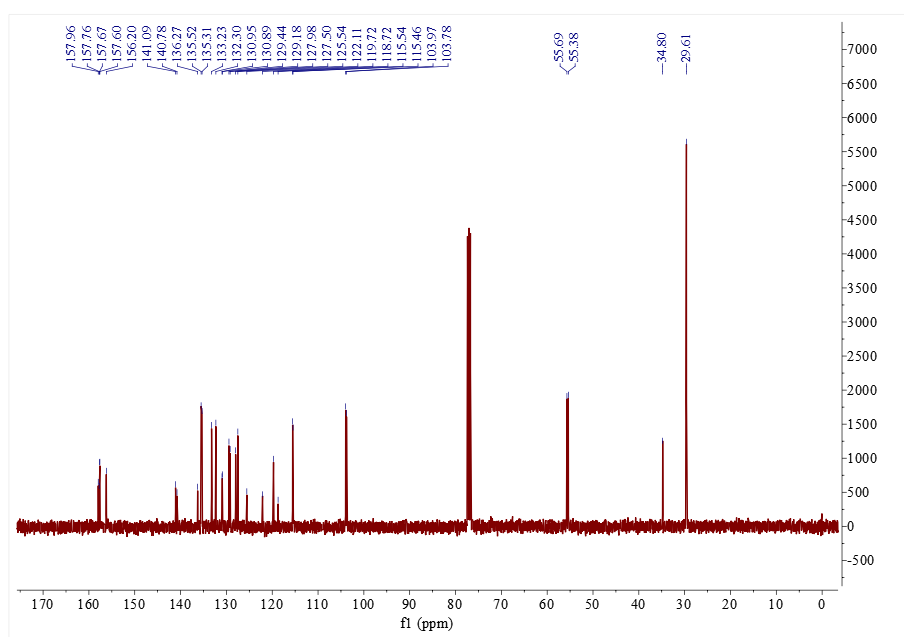


**Supplementary Figure 60**.^13^C NMR spectrum of the **ligand L2.** (CDCl_3_, 101 MHz)


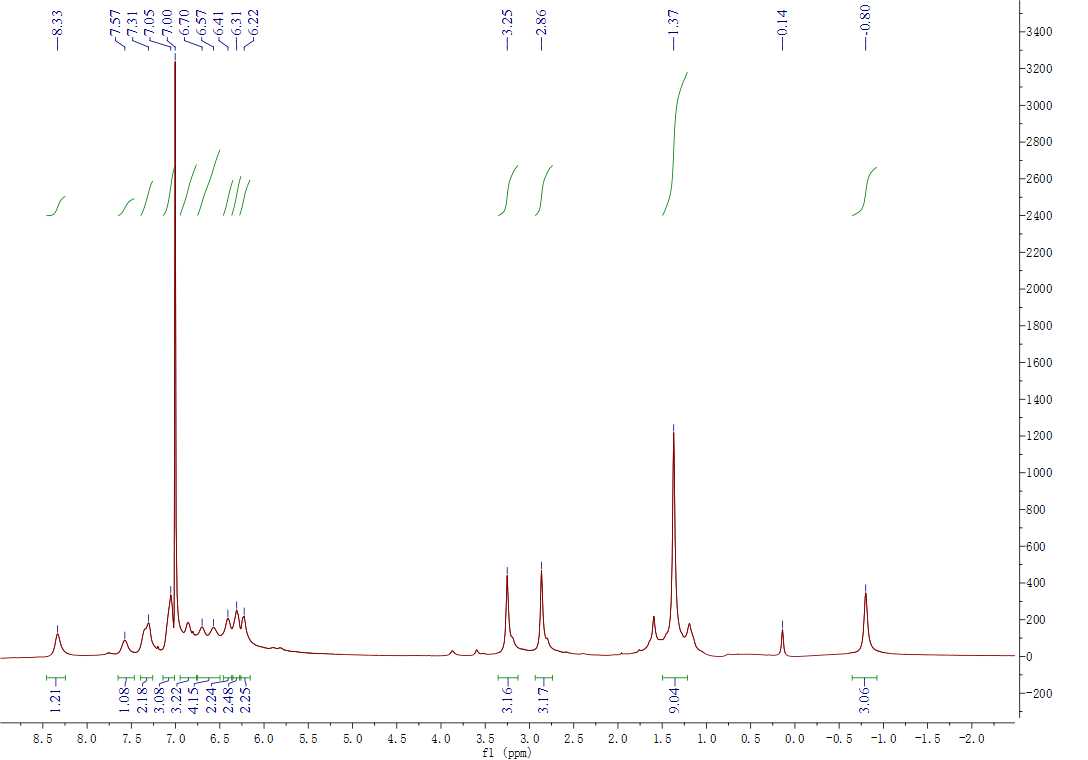


**Supplementary Figure 61**.^1^H NMR spectrum of the **Ni 2.** (C_6_D_6_, 400 MHz)


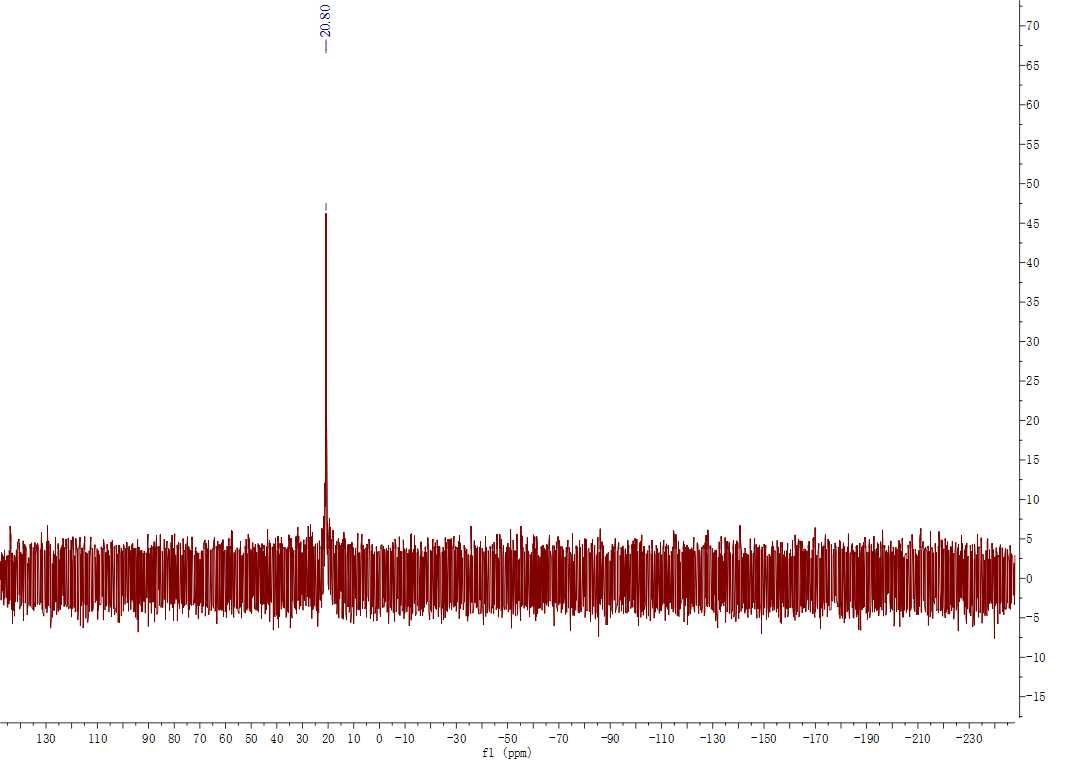


**Supplementary Figure 62**. ^31^P NMR spectrum of the **Ni 2.** (C_6_D_6_, 162 MHz)


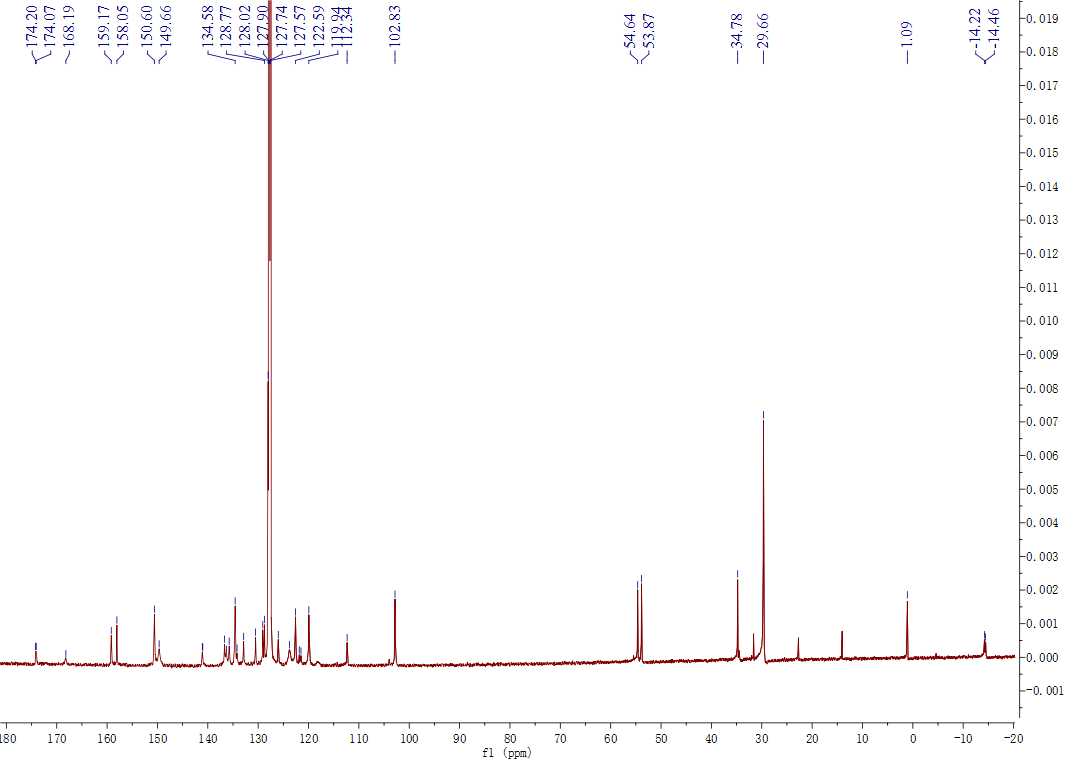


**Supplementary Figure 63**.^13^C NMR spectrum of the **Ni 2.** (C_6_D_6_, MHz)


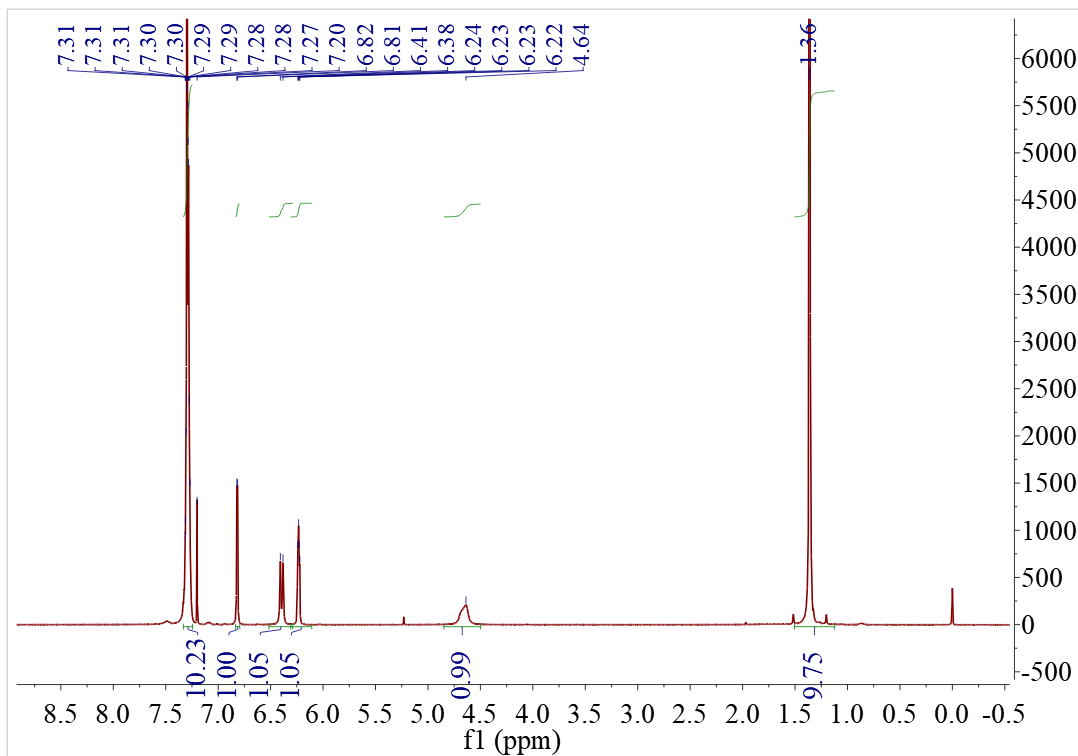


**Supplementary Figure 64**.^1^H NMR spectrum of the **ligand L3.** (CDCl_3_, 400 MHz)


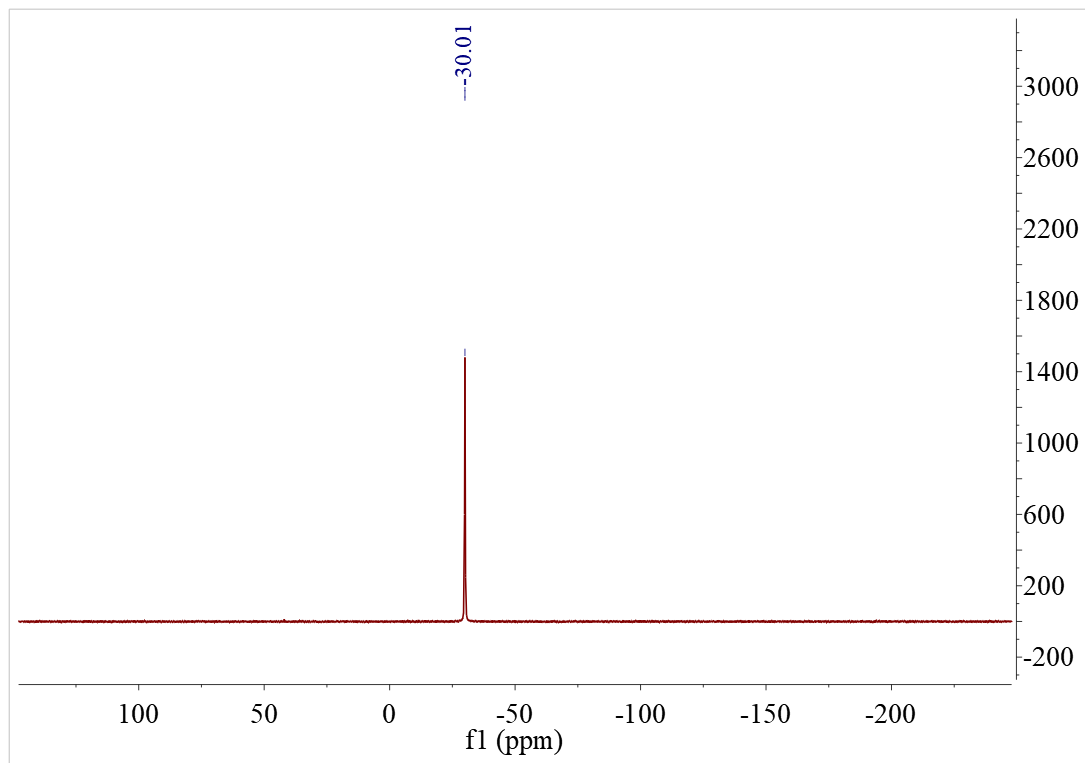


**Supplementary Figure 65**.^31^P NMR spectrum of the **ligand L3.** (CDCl_3_, 162 MHz)


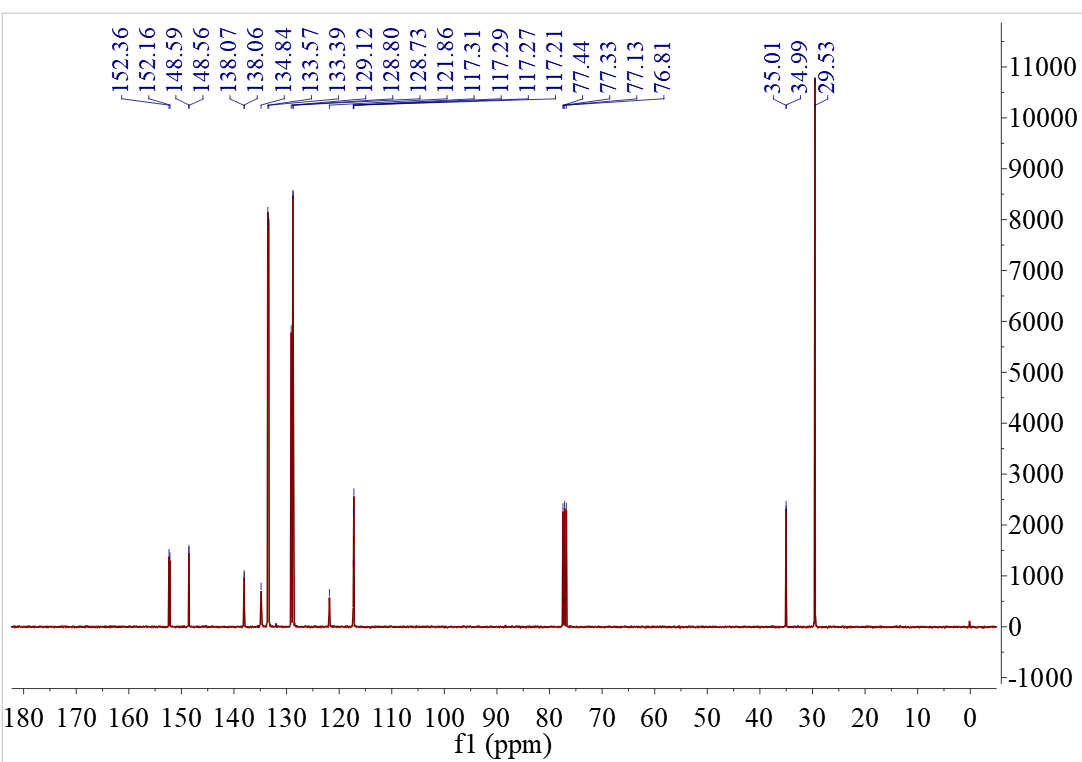


**Supplementary Figure 66**.^13^C NMR spectrum of the **ligand L3.** (CDCl_3_, 101 MHz)


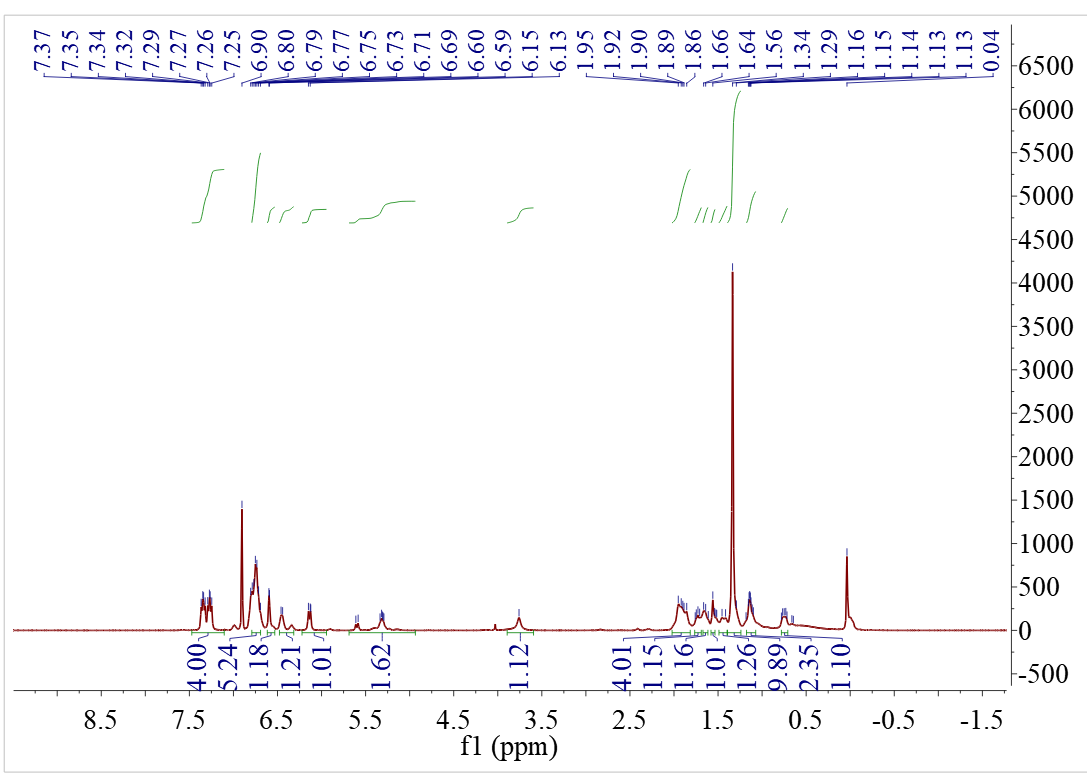


*-tBu*

*CH_2_*

*-OH*

**Supplementary Figure 67**.^1^H NMR spectrum of the **Ni 3**. (C_6_D_6_, 400 MHz)


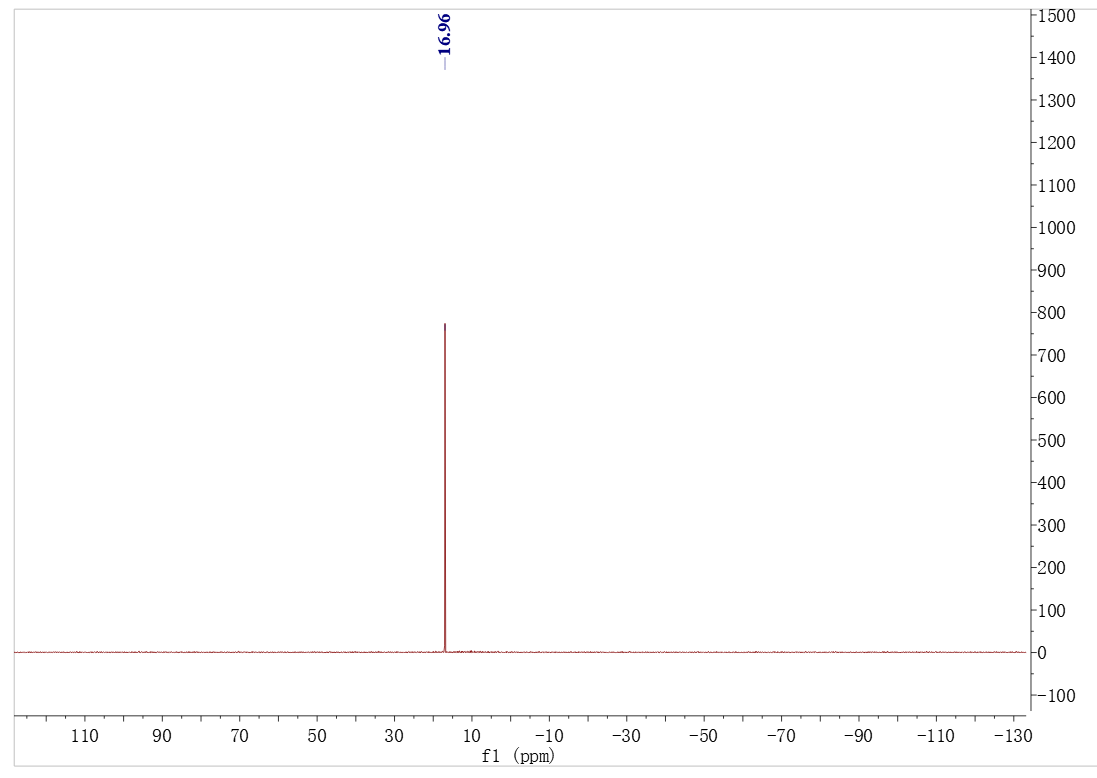


**Supplementary Figure 68**.^31^P NMR spectrum of the **Ni 3**. (C_6_D_6_, 400 162 MHz)


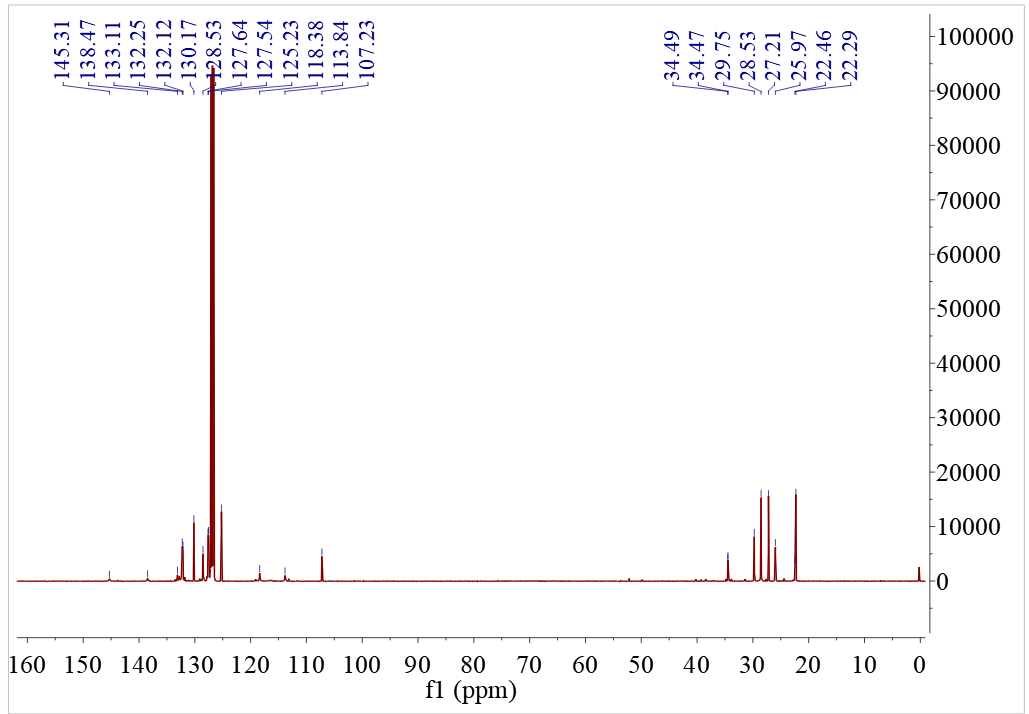


**Supplementary Figure 69**.^13^C NMR spectrum of the **Ni 3**. (C_6_D_6_, 400 101MHz)

**4 Supplementary Figures of NMR of copolymers.**


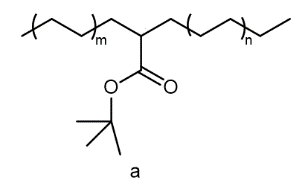

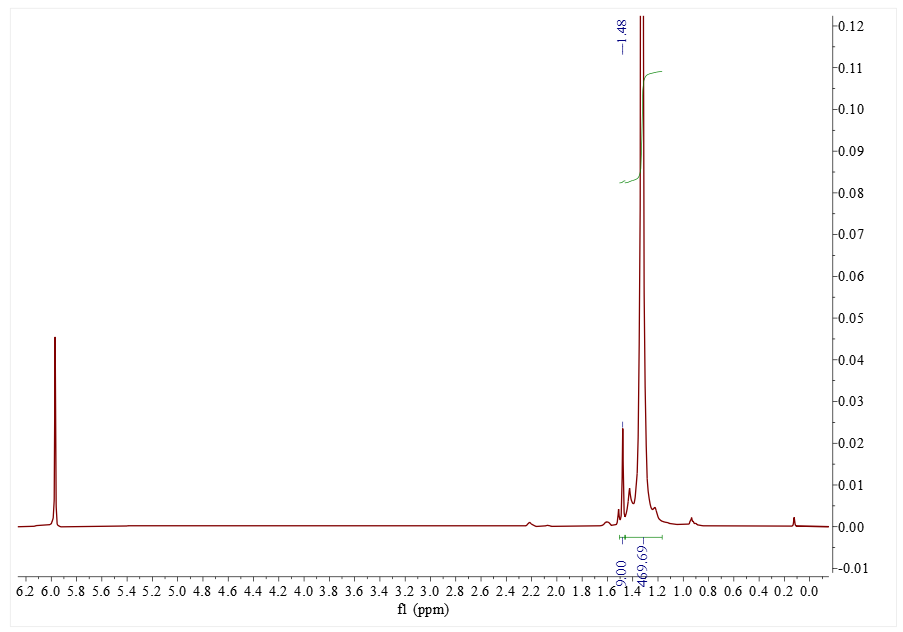


a

**Supplementary Figure 70.** ^1^H NMR spectrum of the polymer from Table 1, Entry 1 (C_2_D_2_Cl_4_, 120^o^C).


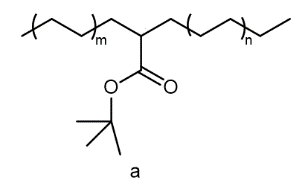

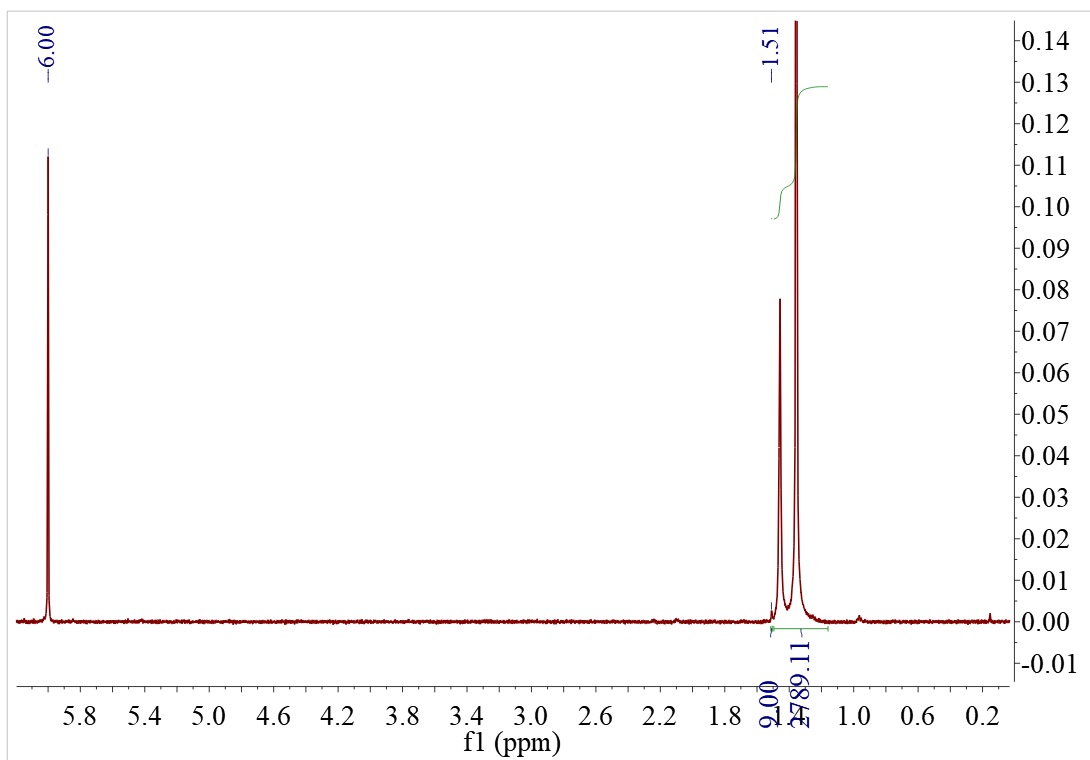


a

**Supplementary Figure 71.** ^1^H NMR spectrum of the polymer from Table 1, Entry 2(C_2_D_2_Cl_4_, 120^o^C).


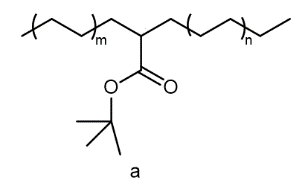

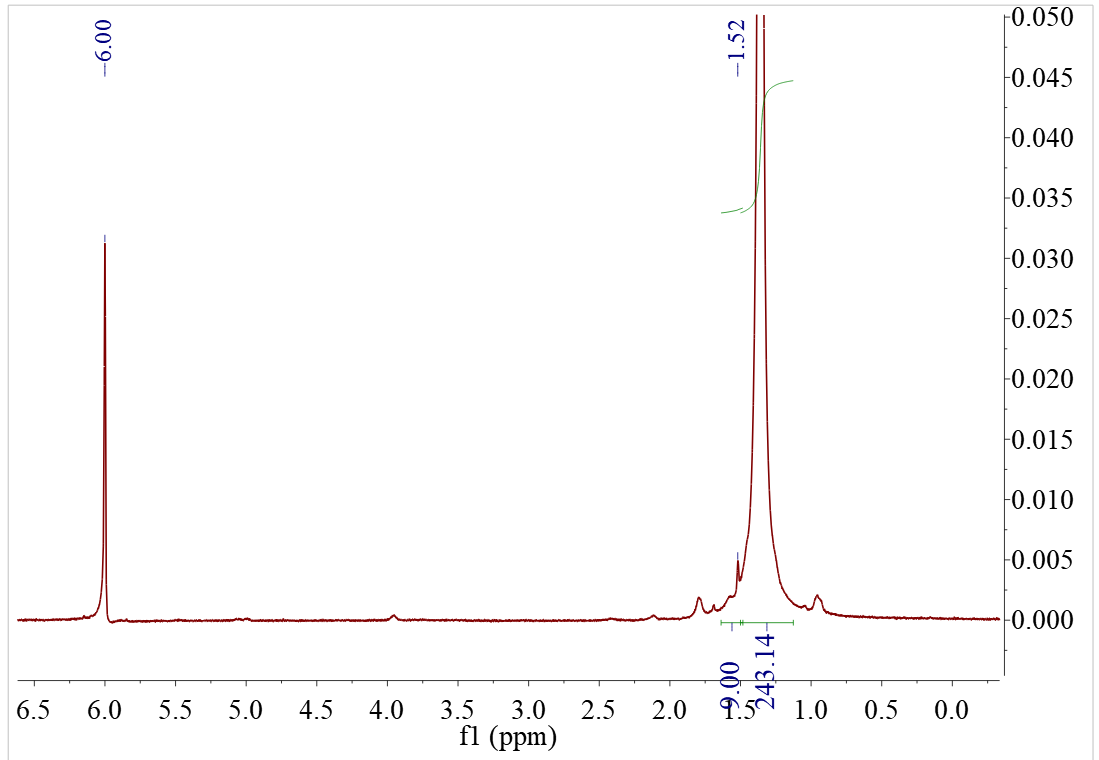


a

**Supplementary Figure 72.** ^1^H NMR spectrum of the polymer from Table 1, Entry 3 (C_2_D_2_Cl_4_, 120^o^C).


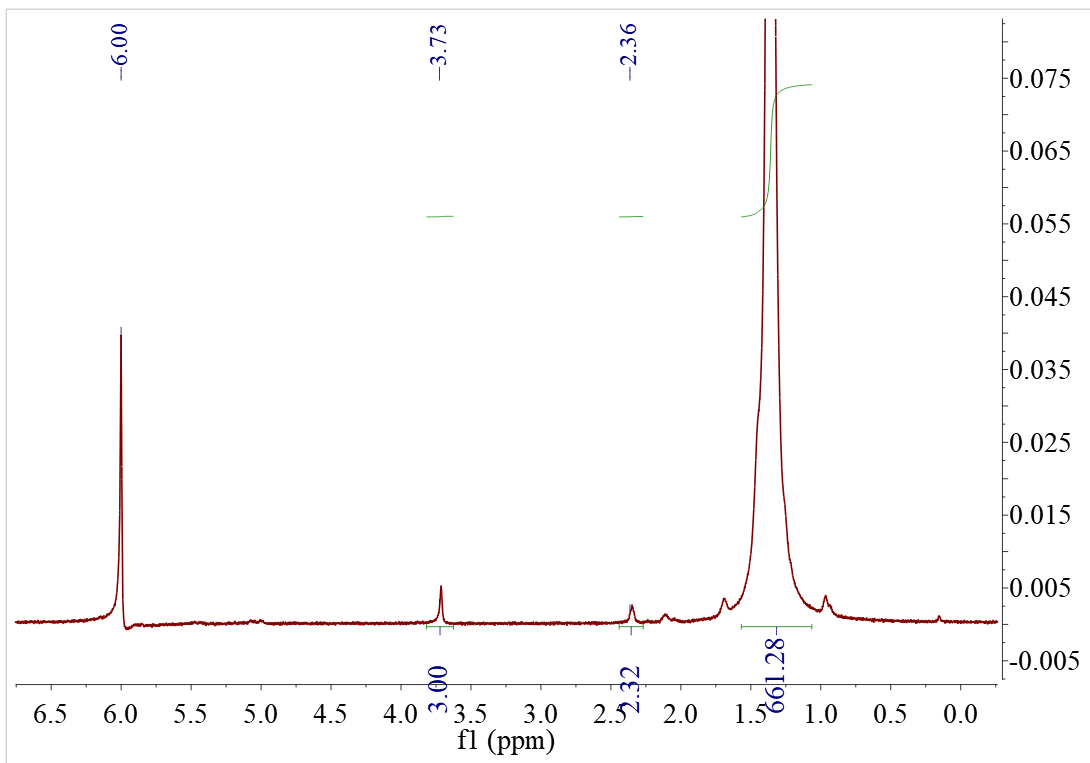


b

a

**Supplementary Figure 73.** ^1^H NMR spectrum of the polymer from Table 1, Entry 4 (C_2_D_2_Cl_4_, 120^o^C).


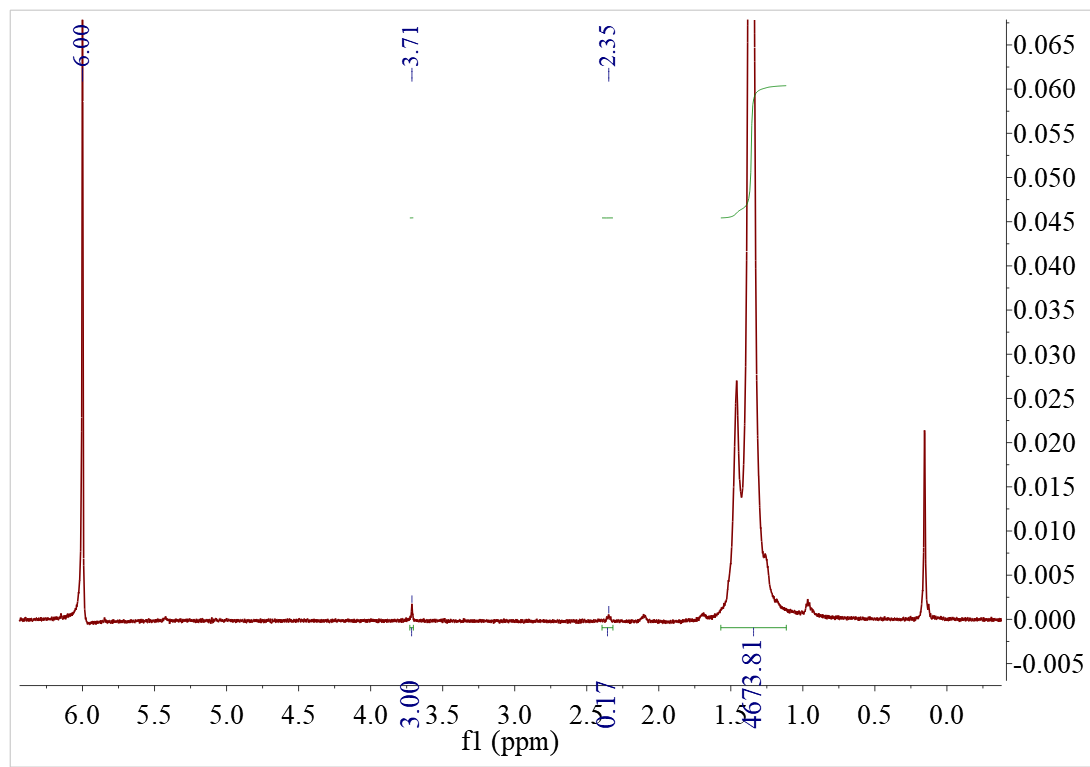


b

a

**Supplementary Figure 74.** ^1^H NMR spectrum of the polymer from Table 1, Entry 5 (C_2_D_2_Cl_4_, 120^o^C).


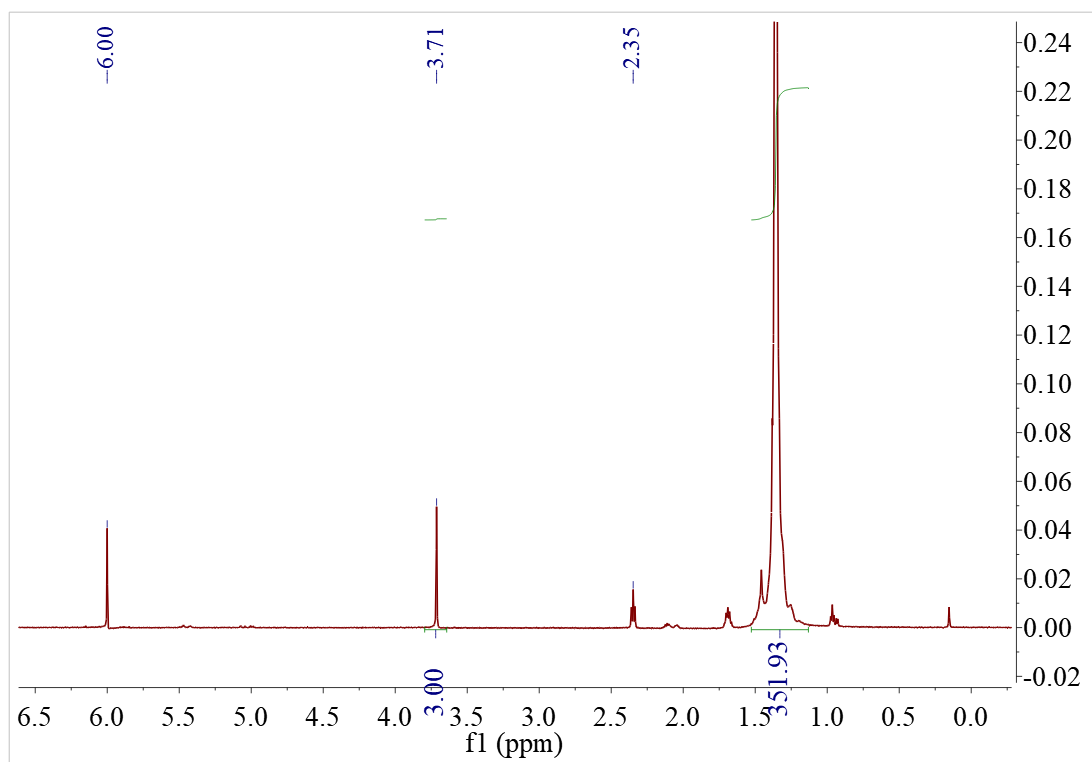


a

b

**Supplementary Figure 75.** ^1^H NMR spectrum of the polymer from Table 1, Entry 6 (C_2_D_2_Cl_4_, 120^o^C).


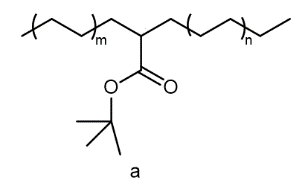

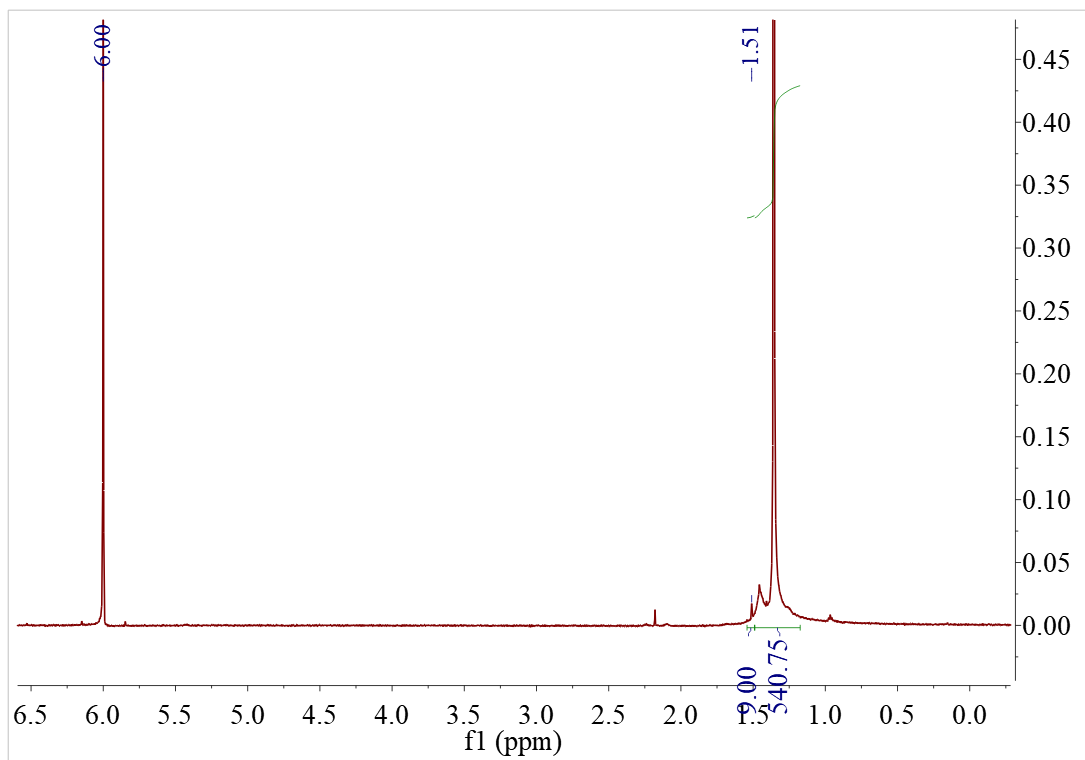


a

**Supplementary Figure 76.** ^1^H NMR spectrum of the polymer from Table 1, Entry 7 (C_2_D_2_Cl_4_, 120^o^C).


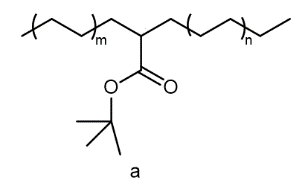

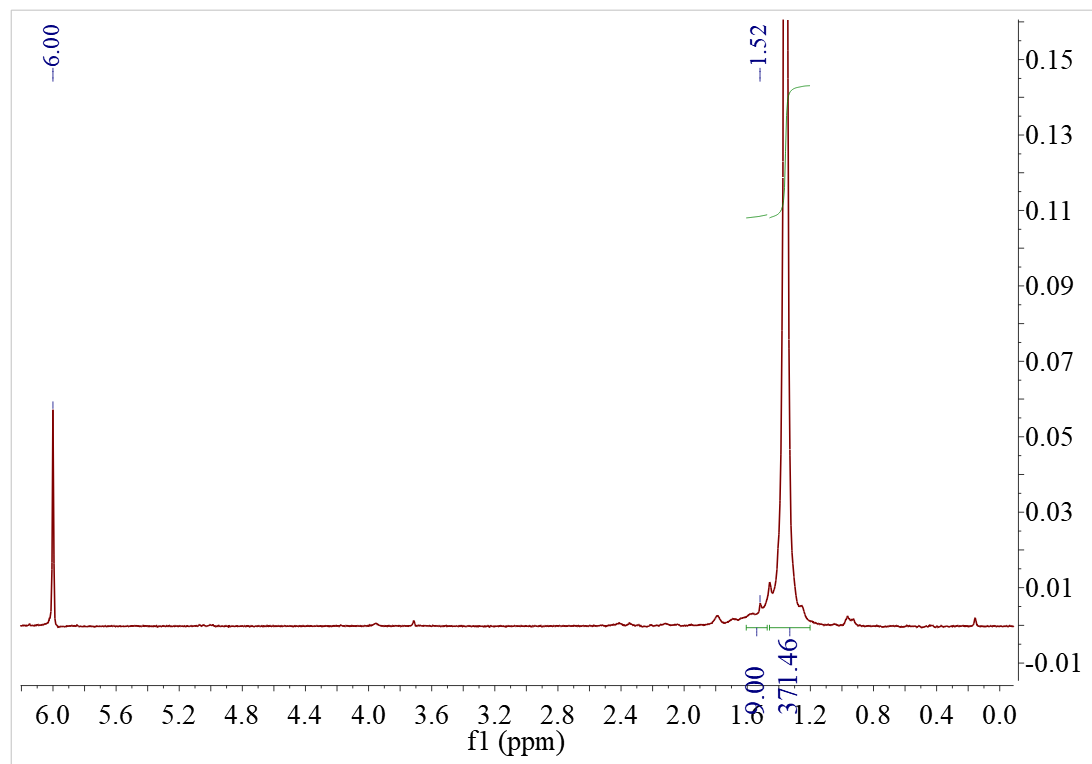


a

**Supplementary Figure 77.** ^1^H NMR spectrum of the polymer from Table 1, Entry 8 (C_2_D_2_Cl_4_, 120^o^C).


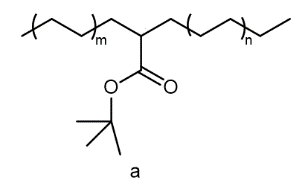

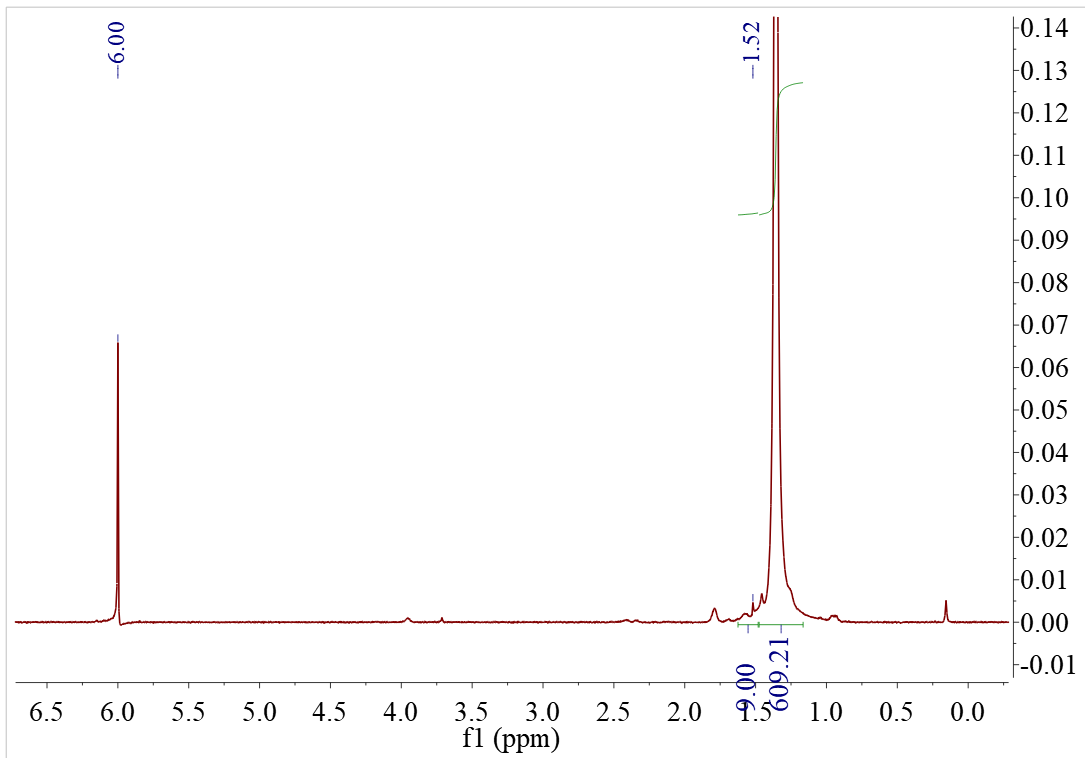


a

**Supplementary Figure 78.** ^1^H NMR spectrum of the polymer from Table 1, Entry 9(C_2_D_2_Cl_4_, 120^o^C).


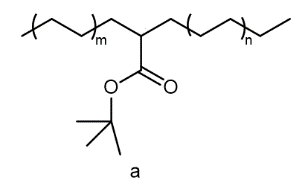

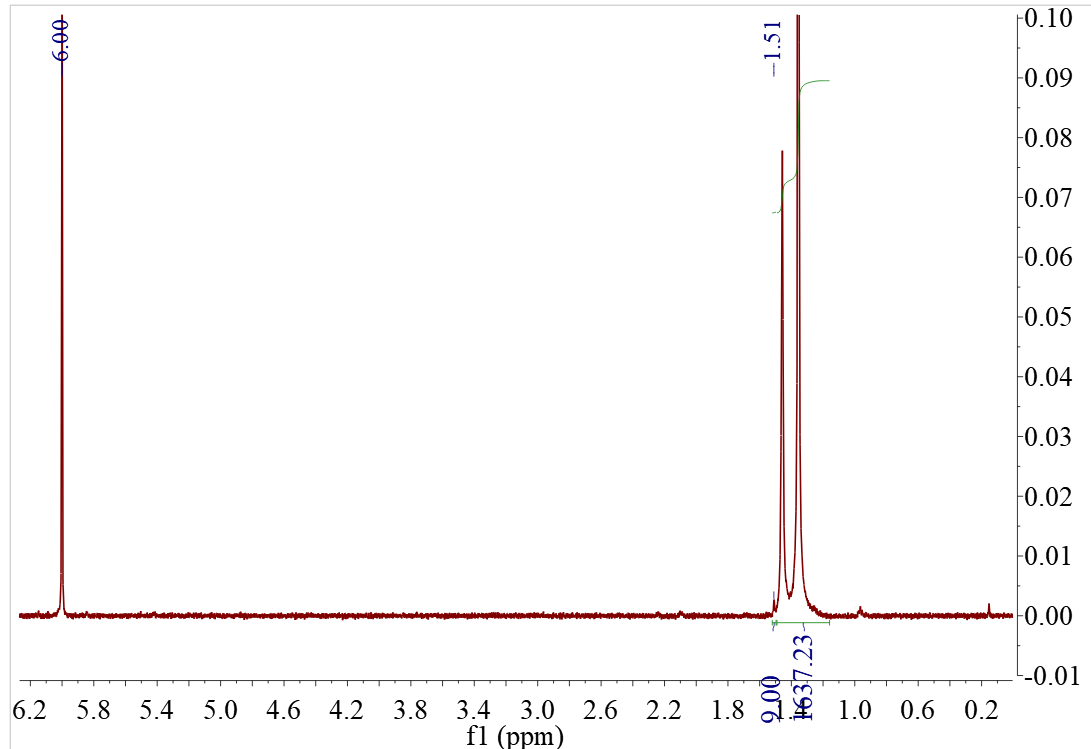


a

**Supplementary Figure 79.** ^1^H NMR spectrum of the polymer from Table 1, Entry 10 (C_2_D_2_Cl_4_, 120^o^C).


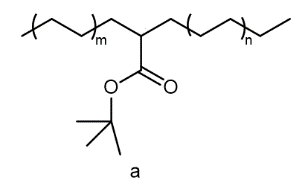

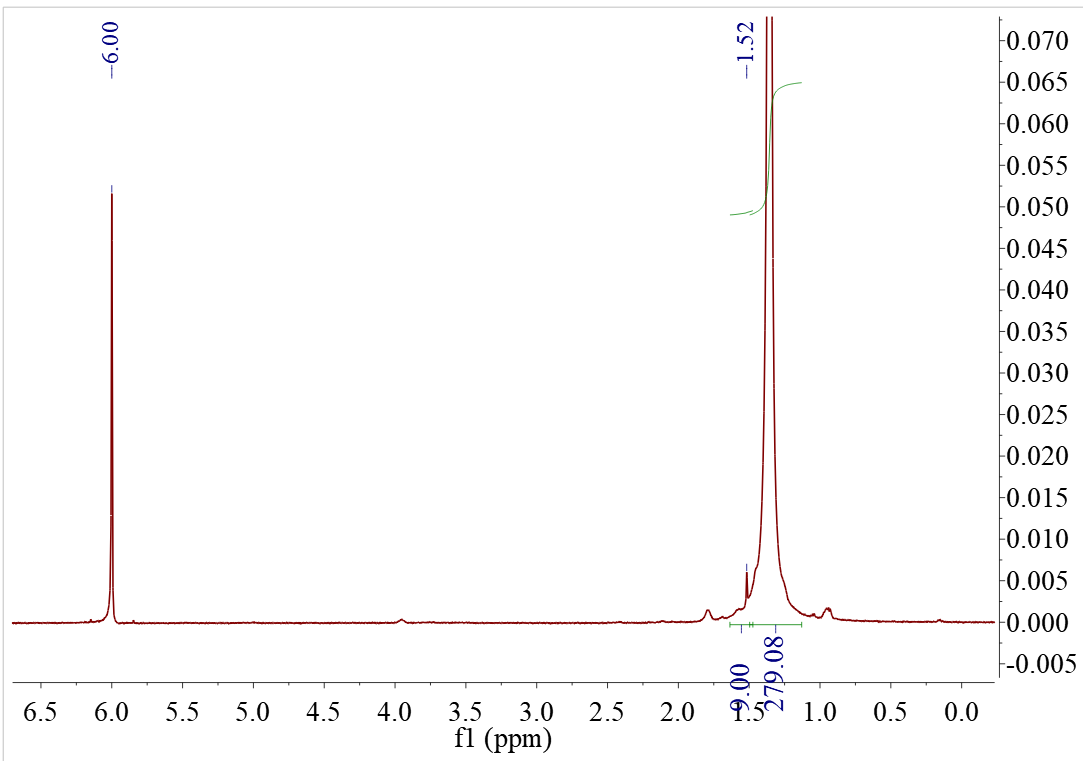


a

**Supplementary Figure 80.** ^1^H NMR spectrum of the polymer from Table 1, Entry 11 (C_2_D_2_Cl_4_, 120^o^C).


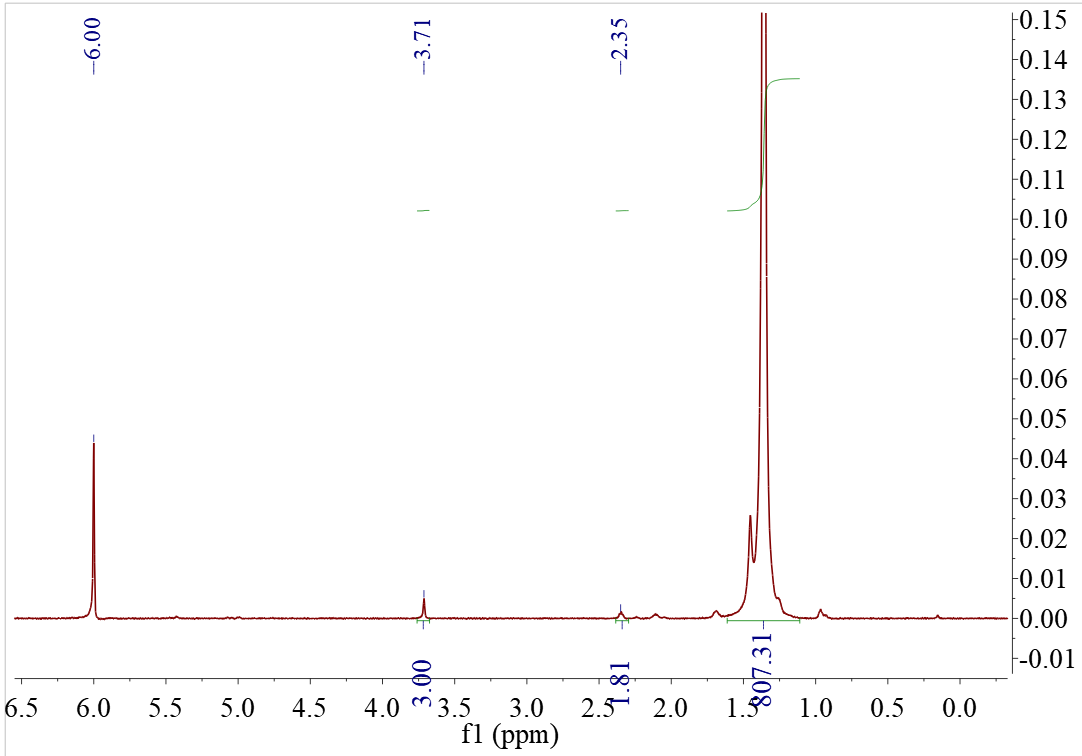


b

a

**Supplementary Figure 81.** ^1^H NMR spectrum of the polymer from Table 2, Entry 12 (C_2_D_2_Cl_4_, 120^o^C).


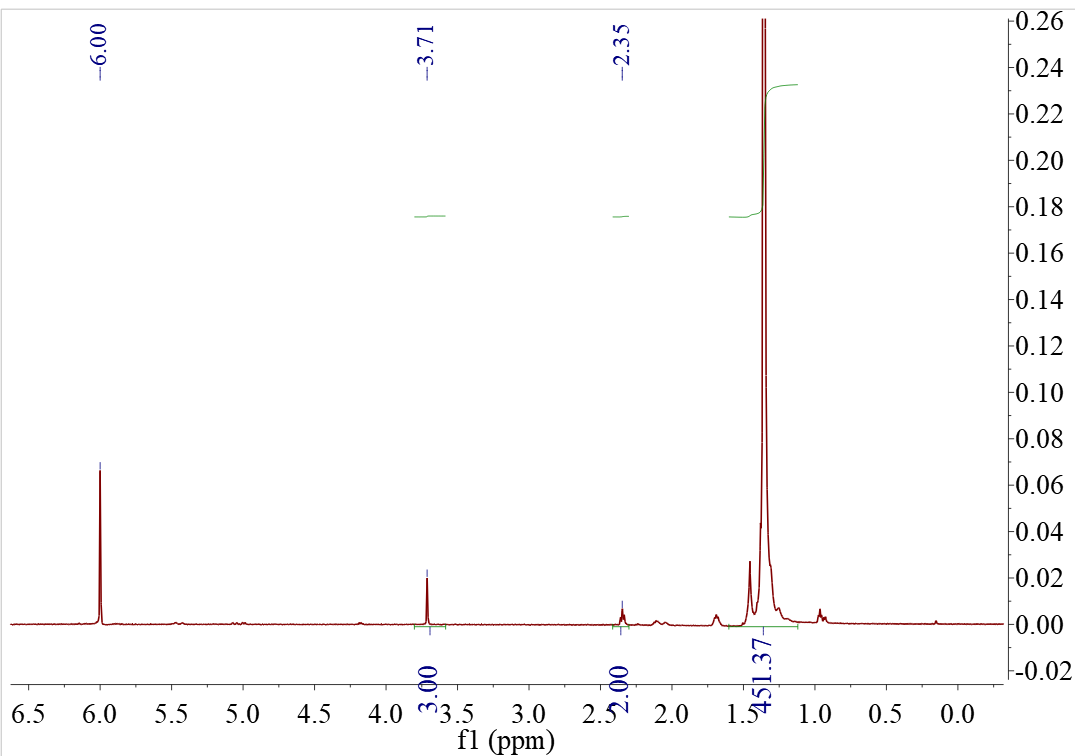


b

a

**Supplementary Figure 82.** ^1^H NMR spectrum of the polymer from Table 1, Entry 13 (C_2_D_2_Cl_4_, 120^o^C).


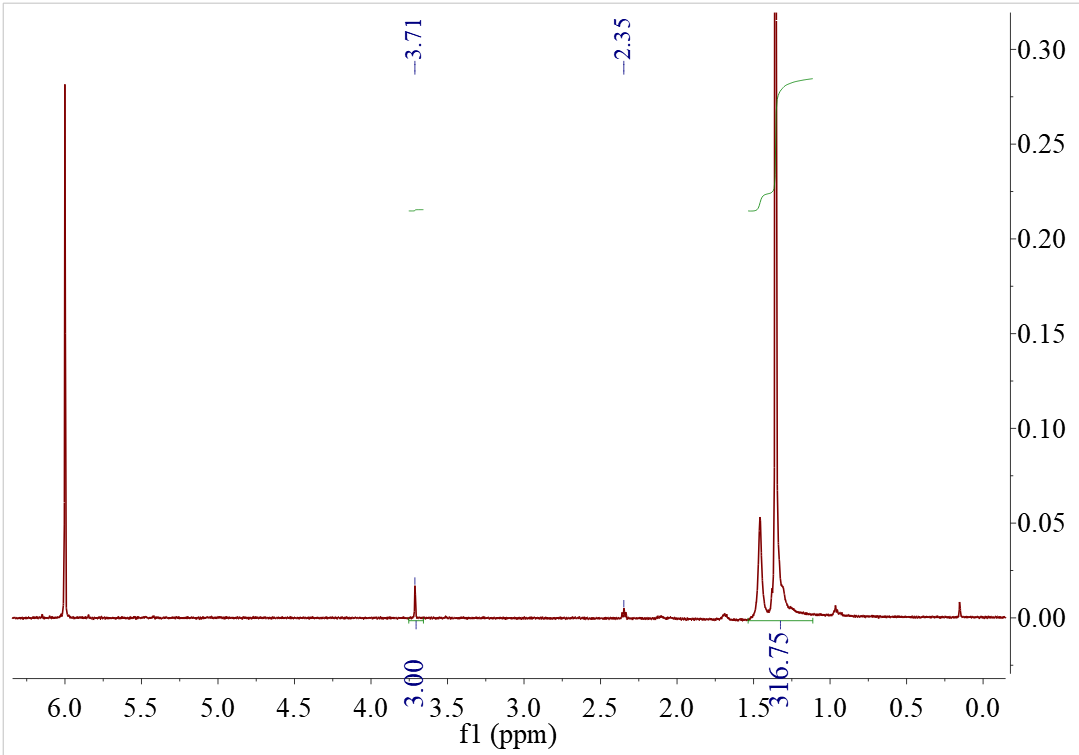


b

a

**Supplementary Figure 83.** ^1^H NMR spectrum of the polymer from Table 1, Entry 14 (C_2_D_2_Cl_4_, 120^o^C).


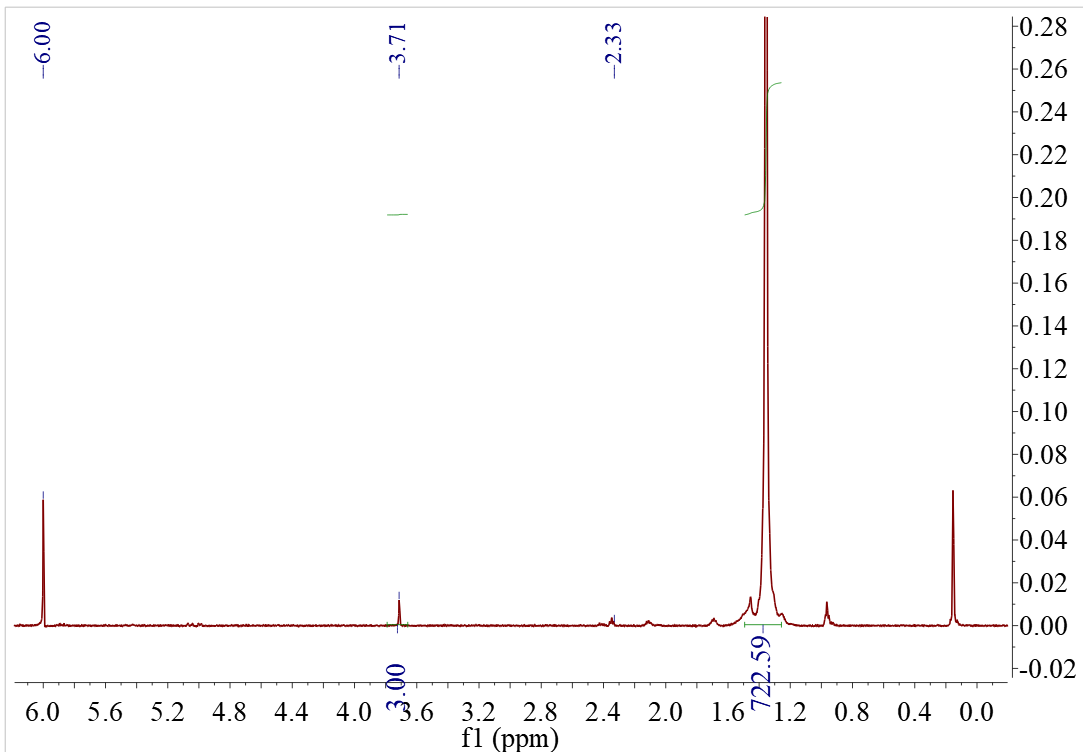


b

a

**Supplementary Figure 84.** ^1^H NMR spectrum of the polymer from Table 1, Entry 15 (C_2_D_2_Cl_4_, 120^o^C).

**
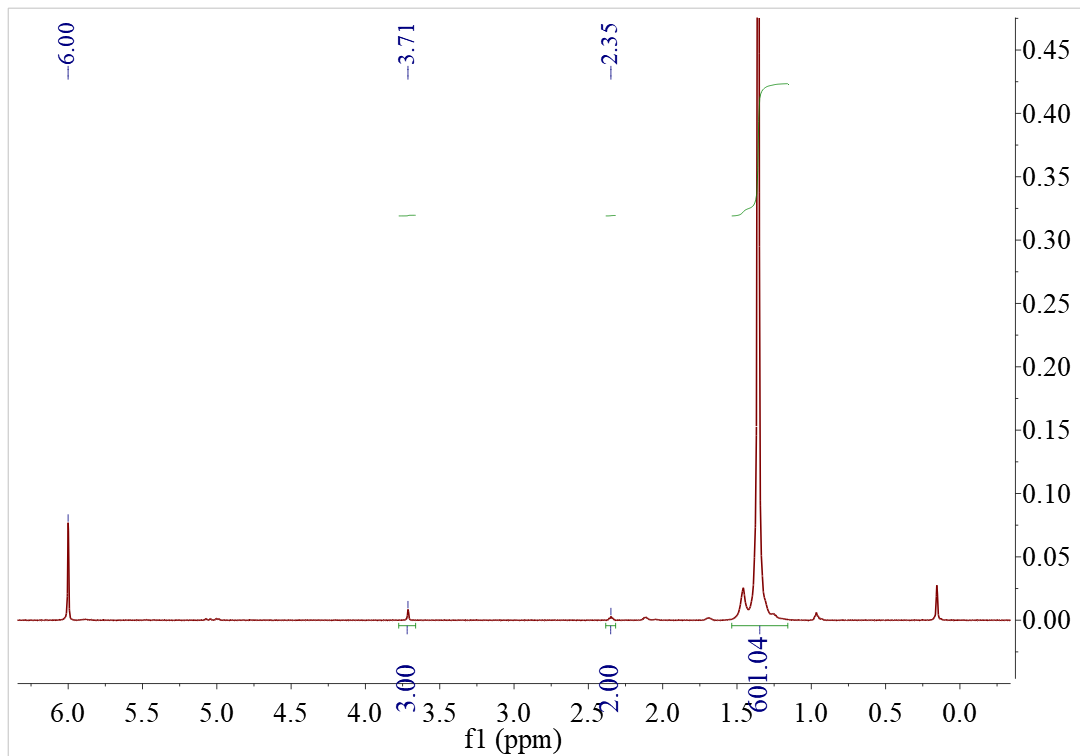
**

b

a

**Supplementary Figure 85.** ^1^H NMR spectrum of the polymer from Table 1, Entry 16 (C_2_D_2_Cl_4_, 120^o^C).


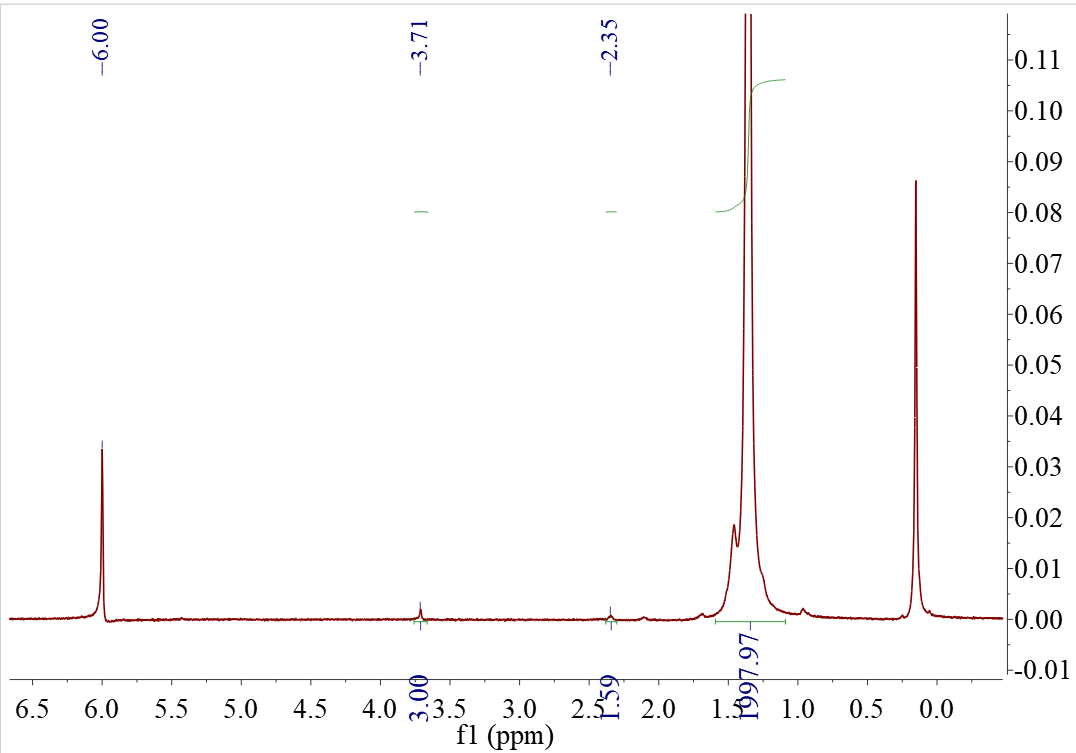


b

a

**Supplementary Figure 86.** ^1^H NMR spectrum of the polymer from Table 1, Entry 17(C_2_D_2_Cl_4_, 120^o^C).


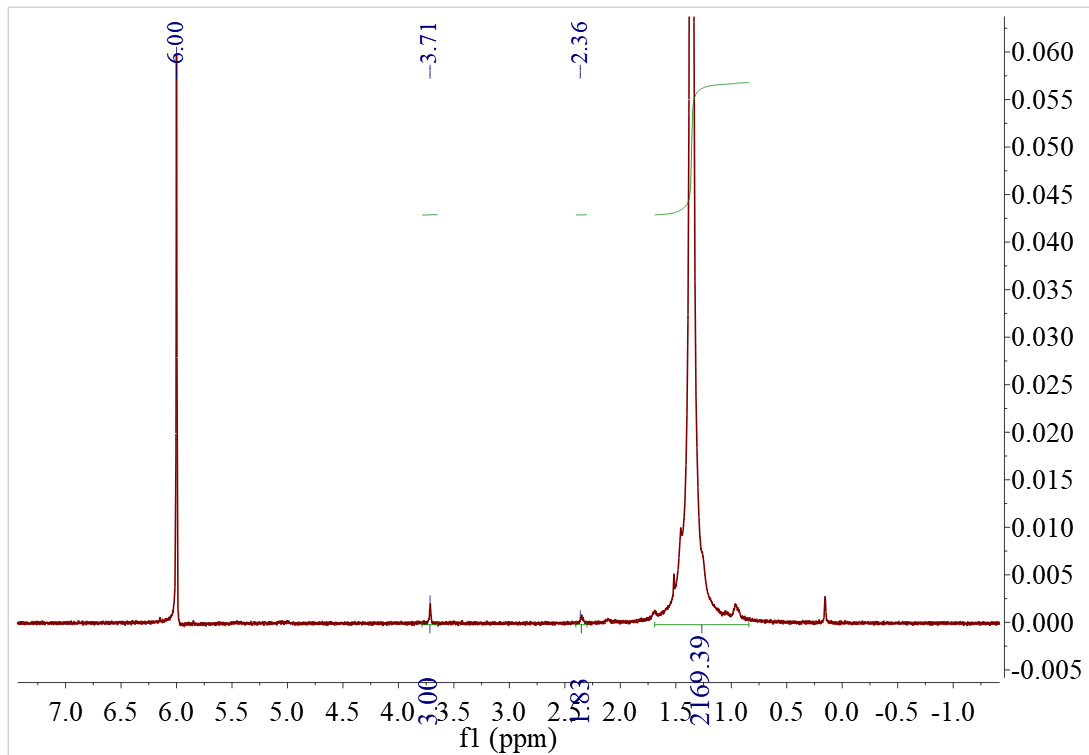


b

a

**Supplementary Figure 87.** ^1^H NMR spectrum of the polymer from Table 1, Entry 18(C_2_D_2_Cl_4_, 120^o^C).


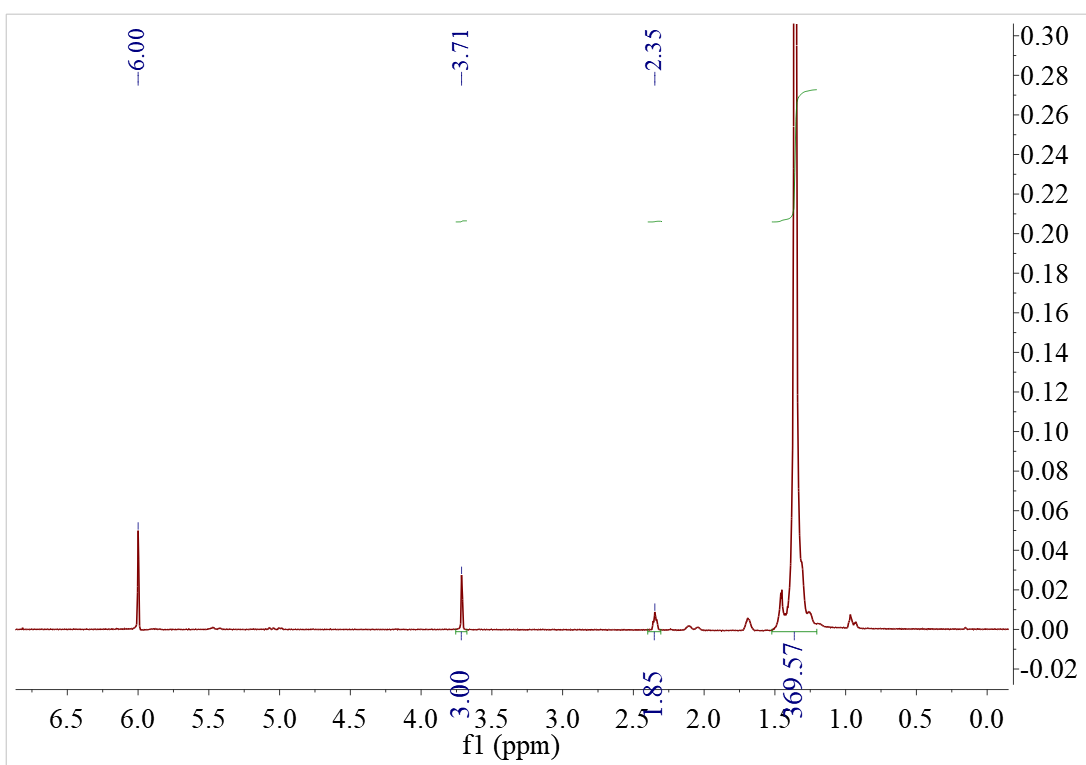


b

a

**Supplementary Figure 88.** ^1^H NMR spectrum of the polymer from Table 1, Entry 19(C_2_D_2_Cl_4_, 120^o^C).


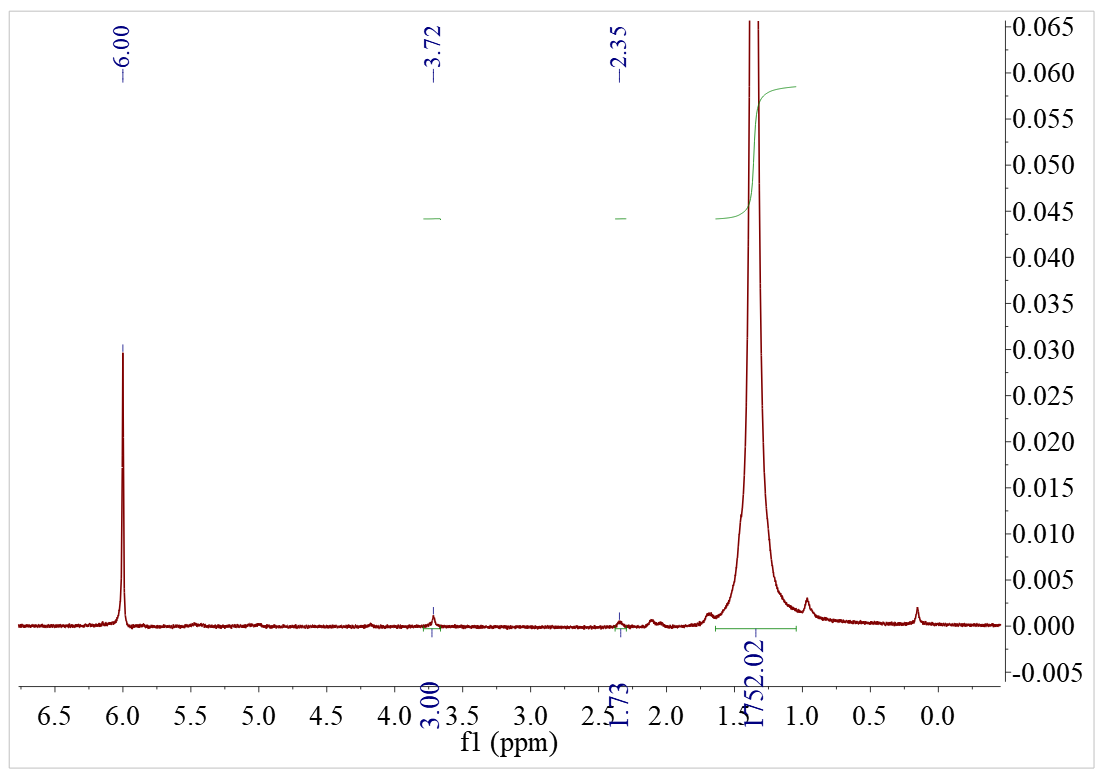


a

b

**Supplementary Figure 89.** ^1^H NMR spectrum of the polymer from Table 1, Entry 20(C_2_D_2_Cl_4_, 120^o^C).


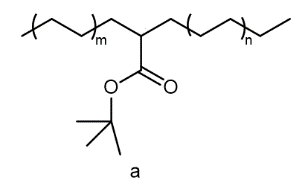


a

**Supplementary Figure 90.** ^1^H NMR spectrum of the polymer from Table 1, Entry 21 (C_2_D_2_Cl_4_, 120^o^C).

b

a

**Supplementary Figure 91.** ^1^H NMR spectrum of the polymer from Table 1, Entry 22(C_2_D_2_Cl_4_, 120^o^C).

b

a

**Supplementary Figure 92.** ^1^H NMR spectrum of the polymer from Supplementary Table 4, Entry 7(C_2_D_2_Cl_4_, 120^o^C).

b

a

**Supplementary Figure 93.** ^1^H NMR spectrum of the polymer from Supplementary Table 4, Entry 8(C_2_D_2_Cl_4_, 120^o^C).

b

a

**Supplementary Figure 94.** ^1^H NMR spectrum of the polymer from Supplementary Table 4, Entry 9(C_2_D_2_Cl_4_, 120^o^C).

b

a

**Supplementary Figure 95.** ^1^H NMR spectrum of the polymer from Supplementary Table 4, Entry 10(C_2_D_2_Cl_4_, 120^o^C).

**5 Supplementary Figures of GPC of polymers.**

**Supplementary Figure 96.** GPC of the polymer from Table 1, Entry 1.

**Supplementary Figure 97.** GPC of the polymer from Table 1, Entry 2.

**Supplementary Figure 98.** GPC of the polymer from Table 1, Entry 3.

**Supplementary Figure 99.** GPC of the polymer from Table 1, Entry 4.

**Supplementary Figure 100.** GPC of the polymer from Table 1, Entry 5.

**Supplementary Figure 101.** GPC of the polymer from Table 1, Entry 6.

**Supplementary Figure 102.** GPC of the polymer from Table 1, Entry 7.

**Supplementary Figure 103.** GPC of the polymer from Table 1, Entry 8

**Supplementary Figure 104.** GPC of the polymer from Table 1, Entry 9.

**Supplementary Figure 105.** GPC of the polymer from Table 1, Entry 10.

**Supplementary Figure 106.** GPC of the polymer from Table 1, Entry 11.

**Supplementary Figure 107.** GPC of the polymer from Table 1, Entry 12.

**Supplementary Figure 108.** GPC of the polymer from Table 1, Entry 13.

**Supplementary Figure 109.** GPC of the polymer from Table 1, Entry 14.

**Supplementary Figure 110.** GPC of the polymer from Table 1, Entry 15.

**Supplementary Figure 111.** GPC of the polymer from Table 1, Entry 16.

**Supplementary Figure 112.** GPC of the polymer from Table 1, Entry 17.

**Supplementary Figure 113.** GPC of the polymer from Table 1, Entry 18.

**Supplementary Figure 114.** GPC of the polymer from Table 1, Entry 19.

**Supplementary Figure 115.** GPC of the polymer from Table 1, Entry 20.

**Supplementary Figure 116.** GPC of the polymer from Table 1, Entry 21.

**Supplementary Figure 117.** GPC of the polymer from Table 1, Entry 22.

**Supplementary Figure 118.** GPC of the polymer from Supplementary Table 1, Entry 1.

**Supplementary Figure 119.** GPC of the polymer from Supplementary Table 1, Entry 2.

**Supplementary Figure 120.** GPC of the polymer from Supplementary Table 1, Entry 3.

**Supplementary Figure 121.** GPC of the polymer from Supplementary Table 1, Entry 4.

**Supplementary Figure 122.** GPC of the polymer from Supplementary Table 1, Entry 5.

**Supplementary Figure 123.** GPC of the polymer from Supplementary Table 1, Entry 6.

**Supplementary Figure 124.** GPC of the polymer from Supplementary Table 1, Entry 7.

**Supplementary Figure 125.** GPC of the polymer from Supplementary Table 1, Entry 8.

**Supplementary Figure 126.** GPC of the polymer from Supplementary Table 1, Entry 9.

**Supplementary Figure 127.** GPC of the polymer from Supplementary Table 1, Entry 10.

**Supplementary Figure 128.** GPC of the polymer from Supplementary Table 1, Entry 11.

**Supplementary Figure 129.** GPC of the polymer from Supplementary Table 1, Entry 12.

**Supplementary Figure 130.** GPC of the polymer from Supplementary Table 1, Entry 13.

**Supplementary Figure 131.** GPC of the polymer from Supplementary Table 1, Entry 14.

**Supplementary Figure 132.** GPC of the polymer from Supplementary Table 1, Entry 15.

**Supplementary Figure 133.** GPC of the polymer from Supplementary Table 1, Entry 16.

**Supplementary Figure 134.** GPC of the polymer from Supplementary Table 1, Entry 17.

**Supplementary Figure 135.** GPC of the polymer from Supplementary Table 1, Entry 18.

**Supplementary Figure 136.** GPC of the polymer from Supplementary Table 3, Entry 1.

**Supplementary Figure 137.** GPC of the polymer from Supplementary Table 3, Entry 2.

**Supplementary Figure 138.** GPC of the polymer from Supplementary Table 3, Entry 3.

**Supplementary Figure 139.** GPC of the polymer from Supplementary Table 3, Entry 4.

**Supplementary Figure 140.** GPC of the polymer from Supplementary Table 4, Entry 5.

**Supplementary Figure 141.** GPC of the polymer from Supplementary Table 4, Entry 8.

**Supplementary Figure 142.** GPC of the polymer from Supplementary Table 4, Entry 9.

**Supplementary Figure 143.** GPC of the polymer from Supplementary Table 4, Entry 10.

**Supplementary Reference**

1. Zou, C.; Si, G. F.;**Chen, C. L.** [A general strategy for heterogenizing olefin polymerization catalysts and the synthesis of polyolefins and composites.](https://www.nature.com/articles/s41467-022-29533-9) Nat. Commun. **2022**, 13, 1954.
